# The relationship between body mass index and neurologic outcomes in survivors of out-of-hospital cardiac arrest treated with targeted temperature management

**DOI:** 10.1371/journal.pone.0265656

**Published:** 2022-03-29

**Authors:** Hyo Jin Bang, Kyu Nam Park, Chun Song Youn, Han Joon Kim, Sang Hoon Oh, Jee Yong Lim, Hwan Song, Soo Hyun Kim, Won Jung Jeong, Hyo Joon Kim, on behalf of the Korean Hypothermia Network Investigator

**Affiliations:** 1 Department of Emergency Medicine, Seoul St. Mary Hospital, College of Medicine, College of Medicine, The Catholic University of Korea, Seoul, South Korea; 2 Department of Emergency Medicine, Eunpyeong St. Mary Hospital, College of Medicine, The Catholic University of Korea, Seoul, South Korea; 3 Department of Emergency Medicine, St. Vincent’s Hospital, The Catholic University of Korea College of Medicine, Suwon, South Korea; Stony Brook University Renaissance School of Medicine, UNITED STATES

## Abstract

**Background:**

The association of body mass index with outcome in patients treated with targeted temperature management (TTM) after out-of-hospital cardiac arrest (OHCA) is unclear. The purpose of this study was to examine the effect of body mass index (BMI) on neurological outcomes and mortality in resuscitated patients treated with TTM after OHCA.

**Methods:**

This multicenter, prospective, observational study was performed with data from 22 hospitals included in the Korean Hypothermia Network KORHN-PRO registry. Comatose adult patients treated with TTM after OHCA between October 2015 and December 2018 were enrolled. The BMI of each patient was calculated and classified according to the criteria of the World Health Organization (WHO). Each group was analyzed in terms of demographic characteristics and associations with six-month neurologic outcomes and mortality after cardiac arrest (CA).

**Results:**

Of 1,373 patients treated with TTM identified in the registry, 1,315 were included in this study. One hundred two patients were underweight (BMI <18.5 kg/m^2^), 798 were normal weight (BMI 18.5–24.9 kg/m^2^), 332 were overweight (BMI 25–29.9 kg/m^2^), and 73 were obese (BMI ≥ 30 kg/m^2^). The higher BMI group had younger patients and a greater incidence of diabetes and hypertension. Six-month neurologic outcomes and mortality were not different among the BMI groups (p = 0.111, p = 0.234). Univariate and multivariate analyses showed that BMI classification was not associated with six-month neurologic outcomes or mortality. In the subgroup analysis, the underweight group treated with TTM at 33°C was associated with poor neurologic outcomes six months after CA (OR 2.090, 95% CI 1.010–4.325, p = 0.047), whereas the TTM at 36°C group was not (OR 0.88, 95% CI 0.249–3.112, p = 0.843).

**Conclusions:**

BMI was not associated with six-month neurologic outcomes or mortality in patients surviving OHCA. However, in the subgroup analysis, underweight patients were associated with poor neurologic outcomes when treated with TTM at 33°C.

## Introduction

The number of obese patients has been steadily increasing worldwide, and in the United States, the proportion increased from 30.5% in 1999–2000 to 42.4% in 2017–2018 [1]. Body mass index (BMI) is a simple tool that uses height and weight to measure obesity. As the number of obese patients increases, an increasing number of studies on the association between body mass index and various diseases has been performed, and the results are controversial. Obesity is known to be a major risk factor for chronic diseases, such as hypertension, type 2 diabetes mellitus, metabolic syndrome, myocardial infarction, and heart failure, which may increase the risk of cardiac arrest [2, 3]. However, the paradox of obesity is that obese patients with sepsis, which has high mortality rates, have better outcomes and survival [4, 5]. Another study found that overweight patients had the lowest mortality rate, and a lower BMI tended to increase all-cause mortality rates [6, 7].

Previous studies examining the relationship between out-of-hospital cardiac arrest (OHCA) outcomes and BMI had controversial results. One single-center, retrospective study showed that an overweight BMI was associated with lower six-month mortality and poor neurologic outcomes in OHCA patients treated with targeted temperature management (TTM) [8]. Another study showed that obesity is a risk factor for mortality in OHCA patients treated with therapeutic hypothermia [9, 10], and other studies showed that BMI was not associated with survival or good neurologic outcomes at hospital discharge [11, 12]. However, in all of the above studies, patients were treated at a target temperature of 33°C, and neurologic outcomes were evaluated at hospital discharge.

In the current study, we aimed to evaluate whether BMI influences six-month neurologic outcomes in patients treated with TTM after cardiac arrest using a multicenter, prospective registry. Through subgroup analysis, we aimed to investigate differences in neurological outcomes according to BMI in the TTM 33 and TTM 36 groups.

## Methods

### Study setting and participation

This registry-based study was conducted prospectively with data from 22 centers included in the Korean Hypothermia Network, KORHN-PRO registry (NCT02827422). The study included an informed consent form approved by all participating hospitals, including the institutional review board (IRB) of Seoul St. Mary’s Hospital (XC150IMI0081K). Between October 2015 and December 2018, comatose adult patients (≥ 18 years old) who were resuscitated following OHCA and treated with TTM were registered in the KORHN-PRO registry. The registry excluded prearrest CPC 3 or 4; known disease resulting in death at 180 days; and cardiac arrest caused by trauma, stroke, or intracranial bleeding. The web-based registry consisted of patient information, resuscitation variables, TTM information, in-hospital treatment, outcomes and outcome prediction modalities. Each center received approval to participate by its Institutional Review Board. Written informed consent was obtained from all patients.

The TTM protocol of all centers included the same TTM parameters. The TTM regimen involved cooling from 32°C to 36°C for 12 to 24 h and controlled normothermia for 72 h after the return of spontaneous circulation. The participating hospitals treated the patients according to their own treatment protocols, and the TTM device and target temperature selection were based on individual preferences. For subgroup analysis, the patients were divided into two groups according to their intended regimen. Patients planning to be treated with a low temperature setting of 33–34°C were considered the TTM 33 group, and those planning to be treated with a high temperature setting of 35–36°C were considered the TTM 36 group. Neurologic outcomes were investigated by researchers at each hospital using multimodal tests.

### Data collection and definitions

We obtained data from the KORHN-PRO registry in the Utstein style. We included adult OHCA survivors who underwent TTM with a target temperature of 32–36°C for 24 h and excluded patients 1) whose weight or height were missed and 2) who had no data on 6-month neurologic outcomes. The following information was obtained: 1) patients characteristics: sex, age, comorbidities (coronary heart disease, hypertension, diabetes mellitus, cardiovascular accident, lung disease and renal disease), height and weight; 2) resuscitation variables: arrest location, initial arrest rhythm, witness of collapse, bystander cardiopulmonary resuscitation (CPR), arrest etiology (cardiac or noncardiac) and anoxic time (collapse to ROSC time); 3) TTM: TTM methods, target temperature, induction time (time from start of TTM to the time of achieving the target temperature), rewarming time, time from ROSC to start of TTM, and the time from ROSC to target temperature achievement; and 4) in-hospital data: shock after ROSC (systolic blood pressure < 90 mmHg for > 30 minutes, or the need for supportive measures to maintain a blood pressure of 90 mmHg), advanced cardiac treatment (coronary angiography, percutaneous coronary intervention, use of thrombolytics, extracorporeal bypass) and adverse events within 7 days to maintain a ROSC (seizure), and advanced cardiac treatment (coronary angiography).

According to the criteria of the World Health Organization (WHO), BMI was calculated as the weight in kilograms (kg) divided by height in meters squared. Based on the weight and height from the registry data, the calculated BMI was categorized as underweight (< 18.5 kg/m^2^), normal weight (18.5–24.9 kg/m^2^), overweight (BMI 25–29.9 kg/m^2^) or obese (≥ 30 kg/m^2^).

### Primary outcome and subgroup analysis

The primary outcome was the neurologic outcome 6 months after resuscitation following OHCA. The outcomes were assessed by investigators at each center through face-to-face or telephone interviews. The neurologic outcome was expressed as good or poor; good performance (CPC 1) or moderate disability (CPC 2) were categorized as a good outcome; and severe disability (CPC 3), vegetative state (CPC 4) or death/brain death (CPC 5) were classified as a poor outcome. Subgroup analysis was performed to assess the effect of BMI on neurologic outcome in terms of patient characteristics and TTM variables.

### Statistical analysis

Recorded data were analyzed using IBM SPSS 24.0 for Windows (IBM corp., Armonk, NY, USA). Continuous variables are presented as the mean ± SD or median and interquartile range (IQR). Categorical variables are presented as counts (percentage). For comparisons among BMI groups, continuous variables were compared using the Kruskal-Wallis test when they did not follow a normal distribution according to the Kolmogorov-Smirnov test. The χ^2^ test was used for comparisons of categorical data. To determine the relationship between BMI and six-month neurologic outcome, univariate and multivariate analyses were performed with logistic regression using normal BMI as the reference category. The Hosmer-Lemeshow test was employed to examine goodness of fit, and odds ratios (ORs) and 95% confidence intervals (CIs) were computed. Subgroup analysis was conducted using forest plots and p-values to investigate the effect of various variables on the association between neurologic outcome and BMI. All statistical tests were two-tailed, and p-values < 0.05 indicated significant differences.

## Results

**Fig 1:**
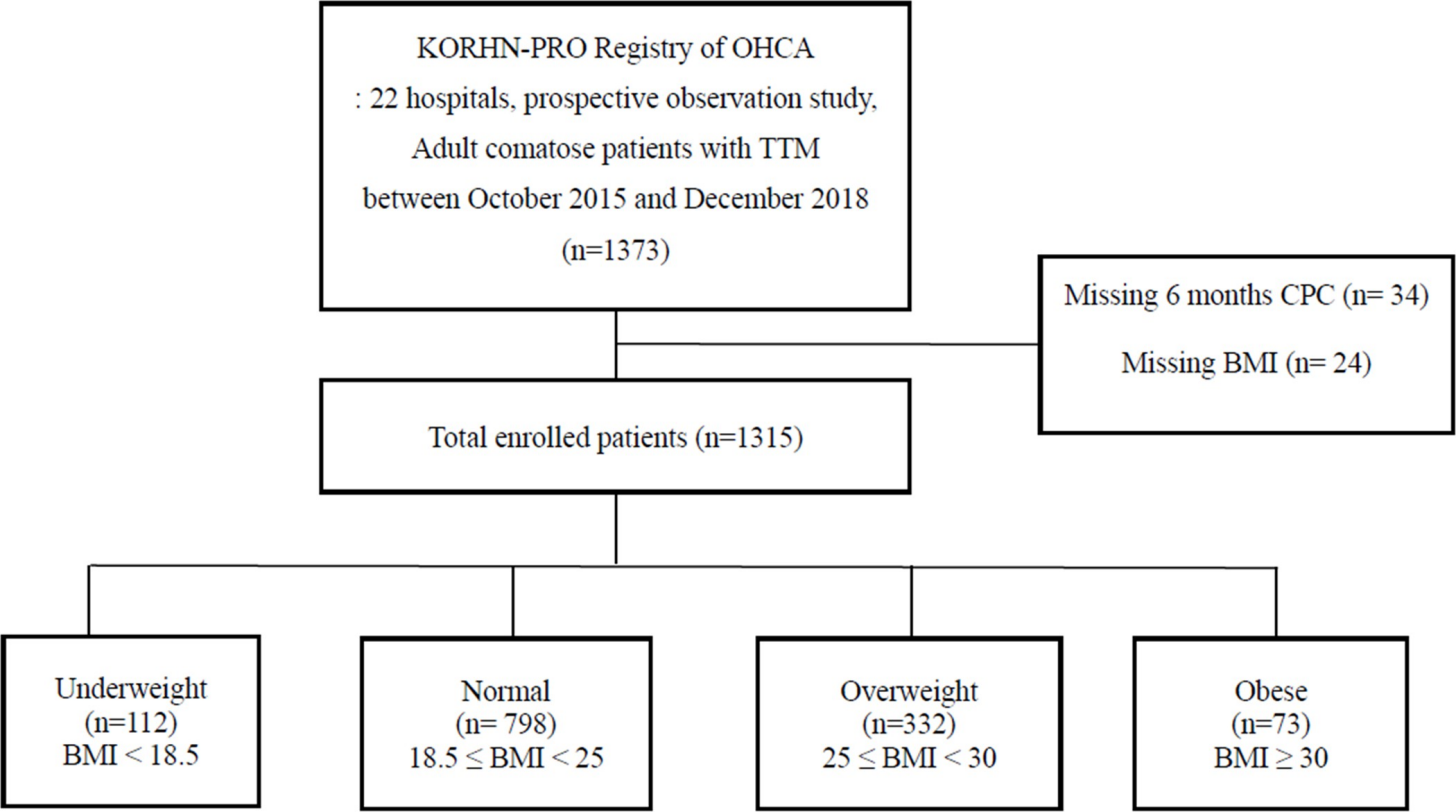
Flow chart of the study. *Abbreviation*: *OHCA* out-of-hospital cardiac arrest, *TTM* targeted temperature management, *BMI* body mass index.

Of 1,373 patients identified in the KORHN-PRO registry, 24 with missing height and weight data and 34 without six-month neurologic outcomes in the database were excluded. Finally, 1,315 patients were enrolled and analyzed in this study. BMI was calculated and categorized into underweight, normal weight, overweight and obese groups according to the WHO criteria (Fig 1).

The baseline characteristics of the patients were analyzed by BMI category (Table 1). The majority of all BMI groups consisted of males (p < 0.001). Significant differences among the groups existed in age, hypertension, diabetes mellitus, lung disease, initial rhythm, arrest etiology, total anoxic time, induction time, ROSC to target temperature and reperfusion treatment. In particular, older patients had lower BMIs (p < 0.001), and patients with higher BMIs had greater incidences of hypertension (p < 0.001), diabetes mellitus (p = 0.003) and witnessed collapse (p = 0.047) and a longer induction time (p < 0.001) and time from ROSC to the target temperature (p < 0.001).

**Table 1:** Baseline characteristics of patients stratified according to body mass index classification.

	Total	Underweight	Normal	Overweight	Obese	p-value
	(n = 1315)	(n = 112)	(n = 798)	(n = 332)	(n = 73)	
Male sex	933 (71.0%)	65 (58.0%)	579 (72.6%)	249 (75.0%)	40 (54.8%)	< 0.001
Age, years, median (IQR)	53.0 (48.0–70.0)	60.0 (47.5–72.0)	59.0 (48.0–70.0)	57.0 (47.0–69.0)	54.0 (44.0–63.0)	0.042
Comorbidities						
CAD	194 (14.8%)	7 (6.3%)	120 (15.0%)	56 (16.9%)	11 (15.1%)	0.053
Hypertension	473 (36.0%)	19 (17.0%)	281 (35.2%)	134 (40.4%)	39 (53.4%)	< 0.001
Diabetes mellitus	318 (24.2%)	15 (13.4%)	192 (24.1%)	84 (25.3%)	27 (37.0%)	0.003
CVA	73 (5.6%)	8 (7.1%)	47 (5.9%)	15 (4.5%)	3 (4.1%)	0.641
Lung disease	84 (6.4%)	22 (19.6%)	41 (5.1%)	17 (5.1%)	4 (5.5%)	< 0.001
Renal disease	100 (7.6%)	6 (5.4%)	57 (7.1%)	28 (8.4%)	9 (12.3%)	0.297
BMI, kg/m^2^, median (IQR)	23.36 (20.96–25.71)	17.30 (16.26–17.96)	22.48 (20.76–23.76)	26.49 (25.71–27.72)	32.67 (31.06–35.23)	< 0.001
Arrest location						0.366
Home/residence	674 (51.3%)	64 (57.1%)	410 (51.4%)	160 (48.2%)	40 (54.8%)	
Other	641 (48.7%)	48 (42.9%)	388 (48.6%)	172 (51.8%)	33 (45.2%)	
Initial arrest rhythm						0.012
Shockable	443 (33.7%)	26 (23.2%)	270 (33.8%)	128 (38.6%)	19 (26.0%)	
Non-shockable	872 (66.3%)	86 (76.8%)	528 (66.2%)	204 (61.4%)	54 (74.0%)	
Witnessed collapse	910 (69.2%)	74 (66.1%)	534 (66.9%)	247 (74.4%)	55 (75.3%)	0.047
Bystander CPR	810 (61.6%)	66 (58.9%)	486 (60.9%)	210 (63.3%)	48 (65.8%)	0.701
Arrest etiology						0.001
Cardiac etiology	812 (61.7%)	52 (46.4%)	492 (61.7%)	226 (68.1%)	42 (57.5%)	
Other etiology	503 (38.3%)	60 (53.6%)	308 (38.3%)	106 (31.9%)	31 (42.5%)	
Anoxic time, minutes, median (IQR)	3.0 (17.0–43.0)	24.0 (13.0–38.0)	28.0 (17.0–40.0)	28.0 (17.0–43.0)	32.0 (15.0–45.5)	0.047
TTM methods						0.756
Surface cooling	1178 (89.6%)	99 (88.4%)	717 (89.8%)	296 (89.2%)	66 (90.4%)	
Intravascular cooling	122 (9.3%)	13 (11.6%)	71 (8.9%)	31 (9.3%)	7 (9.6%)	
Other	15 (1.1%)	0 (0.0%)	10 (1.3%)	5 (1.5%)	0 (0.0%)	
TTM target temperature						**0.275**
33°C	1035 (78.7%)	85 (75.9%)	618 (77.4%)	272 (81.9%)	60 (82.2%)	
36°C	280 (21.3%)	27 (24.1%)	180 (22.6%)	60 (18.1%)	13 (17.8%)	
Induction time, hours, median (IQR)	2.33 (1.08–4.25)	1.42 (1.00–3.50)	2.25 (1.07–4.19)	3.00 (1.83–4.92)	3.75 (1.58–5.88)	< 0.001
Rewarming time, hours, median (IQR)	13.67 (9.27–16.00)	13.00 (8.63–17.80)	13.5 (9.00–16.00)	14.00 (10.00–16.00)	14.00 (8.50–16.00)	0.226
ROSC to TTM, hours, median (IQR)	3.42 (2.17–4.87)	3.62 (2.18–7.21)	3.45 (2.17–5.06)	3.33 (2.20–4.73)	4.00 (2.83–5.13)	0.466
ROSC to target, hours, median (IQR)	6.20 (4.28–9.00)	5.42 (3.89–7.38)	6.22 (4.26–9.00)	6.83 (4.88–9.72)	7.13 (4.93–10.10)	< 0.001
Shock after ROSC	638 (48.5%)	41 (36.6%)	396 (49.8%)	164 (49.5%)	37 (50.7%)	0.067
Reperfusion						< 0.001
None	831 (63.2%)	91 (81.3%)	495 (62.0%)	204 (61.4%)	41 (56.2%)	
CAG only	285 (21.7%)	13 (11.6%)	191 (23.9%)	62 (18.7%)	19 (26.0%)	
CAG with PCI	199 (15.1%)	8 (7.1%)	112 (14.0%)	66 (19.9%)	13 (17.8%)	
tPA	40 (3.0%)	3 (2.7%)	24 (3.0%)	12 (3.6%)	1 (1.4%)	0.774
ECMO	59 (4.5%)	2 (1.8%)	35 (4.4%)	18 (5.4%)	4 (5.5%)	0.428
Adverse events						
Seizure	316 (24.0%)	39 (34.8%)	194 (24.3%)	66 (19.9%)	17 (23.3%)	0.016
Bleeding	64 (4.9%)	5 (4.5%)	40 (5.0%)	17 (5.1%)	2 (2.7%)	0.96
Pneumonia	512 (38.9%)	55 (49.1%)	304 (38.1%)	127 (38.3%)	26 (35.6%)	0.138
Sepsis	153 (11.6%)	15 (13.4%)	89 (11.2%)	39 (11.7%)	10 (13.7%)	0.844
Rearrest	248 (18.9%)	15 (13.4%)	140 (17.5%)	74 (22.3%)	19 (26.0%)	0.044
Six-month neurologic outcome						0.111
Good outcome	410 (31.2%)	25 (22.3%)	262 (32.8%)	104 (31.3%)	19 (26.0%)	
Poor outcome	905 (68.8%)	87 (77.7%)	536 (67.2%)	228 (68.7%)	54 (74.0%)	
Six-month mortality	541 (41.1%)	39 (34.8%)	342 (42.9%)	135 (40.8%)	25 (34.2%)	0.234

*Abbreviations*: *IQR* interquartile range, *CAD* coronary artery disease, *CVA* cerebrovascular accident, *BMI* body mass index, *CPR* cardiopulmonary resuscitation, *TTM* targeted temperature management, ROSC return of spontaneous circulation, CAG coronary angiography, PCI percutaneous coronary intervention, tPA tissue plasminogen activator, *ECMO* extracorporeal membrane oxygenation.

Continuous variables are presented as the median (Q1-Q3) and tested by using the Kruskal-Wallis test, and categorical variables are presented as N (%) and tested by using the chi-squared test.

*p < 0.05 was significant.

The primary neurologic outcomes were compared by BMI classification. Of the 1,315 included patients, 6 months after cardiac arrest, 905 patients had poor neurologic outcomes, and 541 patients had not survived. The number and frequency of patients in each BMI group are presented in Table 1. The results showed no significant difference among the groups.

Univariate and multivariate logistic regression analyses were performed to identify the relationship between BMI and the primary outcome (Table 2). In the univariate analysis, underweight patients were more frequently associated with poor neurologic outcomes (odds ratio [OR], 1.701; 95% confidence interval [CI], 1.065–2.718, p = 0.026). Both univariate and multivariate analyses revealed significant associations with age, initial arrest rhythm, arrest etiology, anoxic time and shock after ROSC. However, in the multivariate logistic regression analysis, we found that BMI was not associated with poor neurologic outcome after adjusting for sex, age, history of hypertension, diabetes mellitus, lung disease, renal disease, arrest location, initial arrest rhythm, witnessed collapse, bystander CPR, arrest etiology, anoxic time, shock after ROSC.

**Table 2:** Univariate and multivariate logistic regression analysis for poor neurologic outcome at 6 months.

Variables	Univariate analysis			Multivariate analysis		
	Unadjusted OR	95% CI for the OR	p-value	Adjusted OR	95% CI for the OR	p-value
Sex (Male/Female)	0.602	0.459–0.790	< 0.001	0.778	0.535–1.131	0.189
Age (per years)	1.032	1.024–1.039	< 0.001	1.044	1.031–1.057	< 0.001
Comorbidities						
Coronary heart disease	1.263	0.917–1.739	0.154			
Hypertension	1.545	1.202–1.987	0.001	1.037	0.705–1.524	0.853
Diabetes mellitus	2.109	1.557–2.857	< 0.001	1.299	0.838–2.013	0.241
CVA	1.409	0.817–2.431	0.218			
Lung disease	4.026	1.996–8.121	< 0.001	0.971	0.425–2.218	0.945
Renal disease	2.334	1.367–3.987	0.002	1.242	0.625–2.466	0.536
Arrest location (Home/Others)	1.674	1.323–2.119	< 0.001	1.092	0.784–1.520	0.603
Initial arrest rhythm (Shockable/Non-shockable)	0.089	0.068–0.117	< 0.001	0.183	0.126–0.264	< 0.001
Witnessed collapse	0.32	0.238–0.431	< 0.001	0.687	0.460–1.026	0.066
Bystander CPR	0.677	0.530–0.866	0.002	1.099	0.780–1.550	0.589
Cardiac arrest etiology	0.135	0.097–0.187	< 0.001	0.232	0.148–0.364	< 0.001
Time from arrest to ROSC (per minutes)	1.061	1.051–1.071	< 0.001	1.064	1.052–1.076	< 0.001
TTM target temperature (33°C/36°C)	0.965	0.726–1.282	0.805			
ROSC to TTM start time (per hours)	0.98	0.940–1.021	0.332			
ROSC to target temperature time (per hours)	1	0.999–1.001	0.513			
Shock after ROSC (Yes/No)	2.819	2.209–3.597	< 0.001	1.888	1.362–2.618	< 0.001
BMI, kg/m^2^						
Normal (15.5–24.9)	Reference	-	-			
Underweight (< 18.5)	1.701	1.065–2.718	0.026	1.668	0.903–3.080	0.102
Overweight (25.0–29.9)	1.072	0.814–1.411	0.622	1.444	0.981–2.125	0.062
Obese (> 30.0)	1.389	0.807–2.392	0.236	1.421	0.684–2.951	0.347

*Abbreviations*: *OR* odds ratio, *CI* confidence interval, *CVA* Cerebrovascular accident, *CPR* cardiopulmonary resuscitation, *TTM* targeted temperature management, ROSC return of spontaneous circulation, *BMI* body mass index.

*p<0.05 was significant.

**Fig 2:**
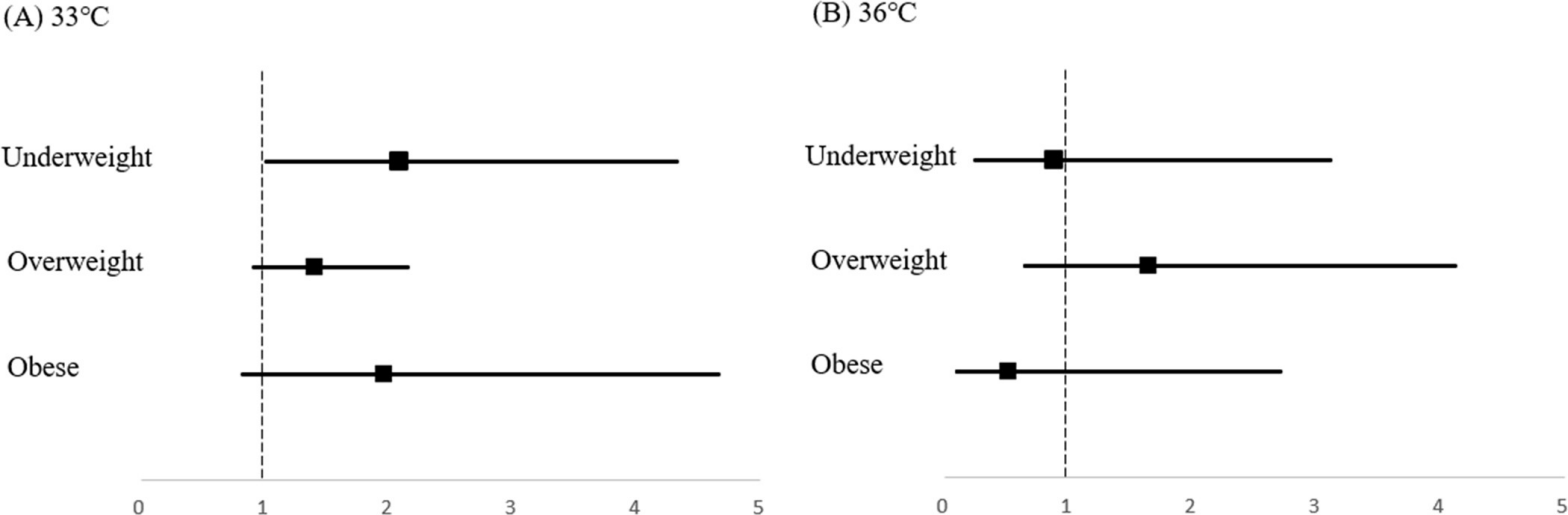
Forest plots of the odds ratios among the BMI groups according to the target temperature. (A) The odds ratio of 33°C targeted temperature management (B) The odds ratio of 36°C targeted temperature management.

Subgroup analysis showed that BMI had no effect on neurologic outcomes except that underweight patients treated with a target temperature of 33°C tended to have poor neurologic outcomes (odds ratio [OR] 2.090; 95% confidence interval [CI] 1.010–4.325; p = 0.047) (Table 3, Fig 2).

**Table 3:** Comparison of odds ratios by body mass index group for poor neurologic outcomes based on the target temperature.

		OR	95% CI for the OR	*p*-value
33°C	Underweight	2.09	1.010–4.325	0.047
	Overweight	1.399	0.908–2.156	0.128
	Obese	1.964	0.827–4.663	0.126
36°C	Underweight	0.88	0.249–3.112	0.843
	Overweight	1.641	0.653–4.123	0.292
	Obese	0.519	0.100–2.704	0.436

*Abbreviations*: *OR* odds ratio, *CI* confidence interval.

*Adjusted sex, age, HTN, DM, lung disease, renal disease, arrest location, initial rhythm, witnessed arrest, bystander CPR, cardiac etiology, TTM shock.

*p<0.05 was significant.

## Discussion

The purpose of this study was to investigate the influence of BMI on the neurologic outcome of OHCA survivors treated with TTM. Because obesity is a risk factor for cardiovascular disease and death [2, 3], obese and overweight patients who survive CA are assumed to have a worse prognosis. When comparing characteristics among the BMI groups, our data showed that patients with higher BMIs had a greater incidence of hypertension and diabetes mellitus. Additionally, obese and overweight patients required more time to reach the target temperature. Although these differences existed, neurologic outcome was not associated with BMI in the univariate and multivariate logistic regression analyses. Only in the subgroup analysis between those treated with target temperatures of 33°C and 36°C was underweight status and treatment at 33°C associated with poor neurologic outcomes.

The association between BMI and OHCA outcomes has been the subject of several studies. Our study found that BMI was not associated with poor neurologic outcomes, although higher BMIs increased the incidence of underlying disease and cardiovascular death. More than half of the enrolled patients were initially found to have a nonshockable rhythm, asystole in particular, which may cause poor outcomes in CA [13]. Moreover, the anoxic time from collapse to ROSC was a significant factor in the neurologic outcome of patients who experienced CA [14]. In the KORHN-PRO registry, the median anoxic time was 30 minutes, which contributed considerably to unfavorable neurologic outcomes. In particular, patients in the obese group had more nonshockable rhythms at arrest and much longer total anoxic times than those in the other groups, while witnessed CA was significantly more common in the obese group. Since the relative importance of BMI and other cardiac arrest factors could not be weighted, the effect of BMI on neurologic outcomes might be affected. One possible explanation for this result is that the ischemia-reperfusion process after ROSC leads to systemic inflammatory injury, called sepsis-like syndrome, and affects patient outcomes [15]. In a previous study on sepsis and BMI, adipose tissue was shown to regulate immunity by secreting anti-inflammatory adipokines and providing fuel in acute life-threatening illness [16, 17]. In this registry, there were some demographic factors that could adversely affect the prognosis of obese patients, but for this reason, the neurological prognosis after 6 months may not be significantly different from that of other groups.

Previous studies demonstrated that a higher BMI was related to a delay in induction time for TTM. Yong Hun Jung et al. showed a prolonged induction duration and slower cooling rate in the obese group [18]. These findings were supported by adipose tissue functioning as an insulator and heat trap [19]. Therefore, obese and overweight patients require a relatively extended time to reach the target temperature, and more aggressive and complex cooling methods might be necessary.

In the subgroup analysis, TTM at 33°C in underweight patients with OHCA resulted in unfavorable neurologic outcomes. This might be explained by underweight patients being sensitive to low temperature and in turn achieving poor outcomes. A prior study highlighted that patients with lower BMIs suffered from more cold injury and peripheral vascular disease than patients with higher BMIs due to a shortage of fat tissue [20]. Furthermore, the results were correlated with the obesity paradox and better outcomes with higher BMIs [21]. In a meta-analysis by Ma, Y., 24,822 patients from 7 studies were analyzed to clarify the relationship between BMI and the clinical outcomes of patients who experienced CA. The study found that overweight patients had a favorable neurological prognosis after CA [22]. Gupta stated that obese patients had higher risk-adjusted odds of survival not only in OCHA but also in IHCA (in-hospital cardiac arrest) [23]. These phenomena resulted from adipose tissue providing a metabolic reserve and lipid soluble nutrients during a highly active metabolic state, such as CA and low temperature during TTM [24]. Consequently, when considering TTM at 33°C for underweight patients resuscitated from OHCA, clinicians should pay special attention to their treatment approach to achieve favorable outcomes.

One of the strengths of this study was that the KORHN-PRO registry contained a large number of OHCA patients from multiple centers. In addition, few patients decided to withdraw life support treatment, and the risk of self-fulfilling prophecy could be avoided. Another strength of this study was that all enrolled patients were provided with well-organized TTM and intensive care. This alleviated treatment gaps among hospitals. Consequently, the body temperature of patients was maintained at a constant temperature, and fluctuation was minimized during TTM. To the best of our knowledge, the current study is the first registry study comparing TTM 33°C and 36°C treatments among BMI groups.

This study had several limitations. First, our study is an observational prospective study, and there is a risk of selection bias and residual confounding. Second, even though the TTM protocol was uniform, clinicians set the target temperature of TTM based on individual preferences, which may influence outcomes. Third, only a few obese patients were included, so the effect of the obese group might be underestimated because the number of Asians with a BMI above 30 was small. Fourth, we used BMI as an indicator of underweight and obesity. Because BMI does not consider body fat distribution or distinguish lean body mass from fat mass, supplementary values could have been applied [25].

## Conclusions

By examining the KORHN-PRO registry, we found that BMI was not an independent risk factor for poor neurologic outcomes at 6 months in CA survivors treated with TTM. In addition, aggressive and additional cooling methods should be considered for patients with higher BMIs, and special attention might be needed for underweight patients in the TTM 33 group.

## Supporting information

S1 Data (PDF)

## References

[1] Hales CM, Carroll MD, Fryar CD, Ogden CL. Prevalence of Obesity and Severe Obesity Among Adults: United States, 2017–2018. NCHS Data Brief. 2020:1–8. 32487284

[2] Lavie CJ, Alpert MA, Arena R, Mehra MR, Milani RV, Ventura HO. Impact of obesity and the obesity paradox on prevalence and prognosis in heart failure. JACC Heart Fail. 2013;1:93–102. doi: 10.1016/j.jchf.2013.01.006 24621833

[3] Ortega-Loubon C, Fernandez-Molina M, Singh G, Correa R. Obesity and its cardiovascular effects. Diabetes Metab Res Rev. 2019;35:e3135. doi: 10.1002/dmrr.3135 30715772

[4] Pepper DJ, Demirkale CY, Sun JF, Rhee C, Fram D, Eichacker P, et al. Does Obesity Protect Against Death in Sepsis? A Retrospective Cohort Study of 55,038 Adult Patients. Critical Care Medicine. 2019;47:643–50. doi: 10.1097/CCM.0000000000003692 30789403 PMC6465121

[5] Mewes C, Bohnke C, Alexander T, Buttner B, Hinz J, Popov AF, et al. Favorable 90-Day Mortality in Obese Caucasian Patients with Septic Shock According to the Sepsis-3 Definition. Journal of Clinical Medicine. 2020;9. 10.3390/jcm9010046 PMC7019854 31878238

[6] Flegal KM, Kit BK, Orpana H, Graubard BI. Association of All-Cause Mortality With Overweight and Obesity Using Standard Body Mass Index Categories A Systematic Review and Meta-analysis. Jama-J Am Med Assoc. 2013;309:71–82. doi: 10.1001/jama.2012.113905 23280227 PMC4855514

[7] Veronese N, Cereda E, Solmi M, Fowler SA, Manzato E, Maggi S, et al. Inverse relationship between body mass index and mortality in older nursing home residents: a meta-analysis of 19,538 elderly subjects. Obes Rev. 2015;16:1001–15. doi: 10.1111/obr.12309 26252230

[8] Jung YH, Lee BK, Lee DH, Lee SM, Cho YS, Jeung KW. The association of body mass index with outcomes and targeted temperature management practice in cardiac arrest survivors. American Journal of Emergency Medicine. 2017;35:268–73. doi: 10.1016/j.ajem.2016.10.070 27836317

[9] Breathett K, Mehta N, Yildiz V, Abel E, Husa R. The impact of body mass index on patient survival after therapeutic hypothermia after resuscitation. American Journal of Emergency Medicine. 2016;34:722–5. doi: 10.1016/j.ajem.2015.12.077 26806177 PMC4801750

[10] Geri G, Savary G, Legriel S, Dumas F, Merceron S, Varenne O, et al. Influence of body mass index on the prognosis of patients successfully resuscitated from out-of-hospital cardiac arrest treated by therapeutic hypothermia. Resuscitation. 2016;109:49–55. doi: 10.1016/j.resuscitation.2016.09.011 27743918

[11] Lee H, Oh J, Kang H, Lim TH, Ko BS, Choi HJ, et al. Association between the body mass index and outcomes of patients resuscitated from out-of-hospital cardiac arrest: a prospective multicentre registry study. Scand J Trauma Resus. 2021;29. doi: 10.1186/s13049-021-00837-x 33509251 PMC7842019

[12] Leary M, Cinousis MJ, Mikkelsen ME, Gaieski DF, Abella BS, Fuchs BD. The association of body mass index with time to target temperature and outcomes following post-arrest targeted temperature management. Resuscitation. 2014;85:244–7. doi: 10.1016/j.resuscitation.2013.10.027 24231571

[13] Lemiale V, Dumas F, Mongardon N, Giovanetti O, Charpentier J, Chiche JD, et al. Intensive care unit mortality after cardiac arrest: the relative contribution of shock and brain injury in a large cohort. Intensive Care Med. 2013;39:1972–80. doi: 10.1007/s00134-013-3043-4 23942856

[14] Grimaldi D, Dumas F, Perier MC, Charpentier J, Varenne O, Zuber B, et al. Short- and long-term outcome in elderly patients after out-of-hospital cardiac arrest: a cohort study. Crit Care Med. 2014;42:2350–7. doi: 10.1097/CCM.0000000000000512 25054671

[15] Adrie C, Adib-Conquy M, Laurent I, Monchi M, Vinsonneau C, Fitting C, et al. Successful cardiopulmonary resuscitation after cardiac arrest as a "sepsis-Like" syndrome. Circulation. 2002;106:562–8. doi: 10.1161/01.cir.0000023891.80661.ad 12147537

[16] Ahima RS. Adipose tissue as an endocrine organ. Obesity. 2006;14:242–9. doi: 10.1038/oby.2006.317 17021375

[17] Zeve D, Tang W, Graff J. Fighting Fat with Fat: The Expanding Field of Adipose Stem Cells. Cell Stem Cell. 2009;5:472–81. doi: 10.1016/j.stem.2009.10.014 19896439 PMC2876189

[18] Jung YH, Lee BK, Lee DH, Lee SM, Cho YS, Jeung KW. The association of body mass index with outcomes and targeted temperature management practice in cardiac arrest survivors. Am J Emerg Med. 2017;35:268–73. doi: 10.1016/j.ajem.2016.10.070 27836317

[19] Speakman JR. Obesity and thermoregulation. Handb Clin Neurol. 2018;156:431–43. doi: 10.1016/B978-0-444-63912-7.00026-6 30454605

[20] Min JY, Choi YS, Lee HS, Lee S, Min KB. Increased cold injuries and the effect of body mass index in patients with peripheral vascular disease. BMC Public Health. 2021;21:294. doi: 10.1186/s12889-020-09789-w 33579232 PMC7881551

[21] Lavie CJ, De Schutter A, Parto P, Jahangir E, Kokkinos P, Ortega FB, et al. Obesity and Prevalence of Cardiovascular Diseases and Prognosis-The Obesity Paradox Updated. Prog Cardiovasc Dis. 2016;58:537–47. doi: 10.1016/j.pcad.2016.01.008 26826295

[22] Ma Y, Huang L, Zhang L, Yu H, Liu B. Association between body mass index and clinical outcomes of patients after cardiac arrest and resuscitation: A meta-analysis. Am J Emerg Med. 2018;36:1270–9. doi: 10.1016/j.ajem.2018.03.079 29678294

[23] Gupta T, Kolte D, Mohananey D, Khera S, Goel K, Mondal P, et al. Relation of Obesity to Survival After In-Hospital Cardiac Arrest. Am J Cardiol. 2016;118:662–7. doi: 10.1016/j.amjcard.2016.06.019 27381664

[24] Sakr Y, Elia C, Mascia L, Barberis B, Cardellino S, Livigni S, et al. Being overweight or obese is associated with decreased mortality in critically ill patients: a retrospective analysis of a large regional Italian multicenter cohort. J Crit Care. 2012;27:714–21. doi: 10.1016/j.jcrc.2012.08.013 23102526

[25] Antonopoulos AS, Tousoulis D. The molecular mechanisms of obesity paradox. Cardiovasc Res. 2017;113:1074–86. doi: 10.1093/cvr/cvx106 28549096

