# Supplementary material for: The relationship between body mass index and neurologic outcomes in survivors of out-of-hospital cardiac arrest treated with targeted temperature management
Source: PLoS One. 2022 Mar 29;17(3):e0265656. doi: 10.1371/journal.pone.0265656 (PMC8963585; doi:10.1371/journal.pone.0265656)
Supplement: S1 Data — (PDF) [file pone.0265656.s001.pdf]

1: Male<br>2: Female		1: home/residence<br>2: others	1: Y<br>2: N	1: Y<br>2: N	1: shockable<br>2: non-shockable	0: NO<br>1: OBESE	1:Y<br>2:N	1:Y<br>2:N	1:Y<br>2:N	1:Y<br>2:N	1:Y<br>2:N	0: NO<br>1: PCI		1: CARDIAC<br>2: OTHER	0: ≥90<br>1: <90 (SHOCK)	0: 33<br>1: 36	1: surface cooling<br>2: endovascular cooling<br>3: ETC	1: feedback device-surface-hydrogel pad<br>2: feedback device-surface-water circulating blanket or garment<br>3: feedback device-surface-immersion<br>4: feedback device-endovascular<br>5: ice pack<br>6: cold saline<br>7: cold gastric lavage<br>8: cold bladder irrigation<br>9: extracorporeal cooling<br>10: others	1: feedback device-surface-hydrogel pad<br>2: feedback device-surface-water circulating blanket or garment<br>3: feedback device-surface-immersion<br>4: feedback device-endovascular<br>5: ice pack<br>6: cold saline<br>7: cold gastric lavage<br>8: cold bladder irrigation<br>9: extracorporeal cooling<br>10: others	1: Y<br>2: N<br>3: unknown		1: Y<br>2: N	1: Y<br>2: N	1: Y<br>2: N	1: Y<br>2: N	1: CAG without PCI<br>2: CAG with PCI<br>3: N	0: NO<br>1: ABN	1: Y<br>2: N	1: Y<br>2: N	1: Y<br>2: N	1: Y<br>2: N		mg/dl	1: Y<br>2: N	1: Y<br>2: N	1: Y<br>2: N	1: Cardiovascular<br>2: cerebral<br>3: MOF<br>4: other	0: GOOD<br>1: POOR	1: Y<br>2: N	0: GOOD<br>1: POOR
SEX	AGE	ARREST_LOCATION	WINTESSED_YN	BYSTANDER_YN	Shockable_YN	OBESITY	HX_CAD	HX_HTN	HX_DM	HX_CVA	HX_KIDNEY	HX_PCI	Ischemic_TIME_MIN	CARDIAC_ETIOLOGY	ROSC_SBP90	TARGET_TMPERATURE_33_36	TTM_METHOD	TTM_METHODOLOGY_FROM	TTM_METHODOLOGY_AFTER	ICU_ADMISSION	ICU_ADMISSION_DATE	ECMO	tPA	CAG only	PCI	CAG_CATE	RWMA	BLEEDING	INFECTION	PNEUMONIA	SEPSIS	CRP	PEAK_CRP	RRT	REARREST	SURVIVAL_DC	IN_HOSPITAL_DEATH	OUTCOME_DC	SURVIVAL_6M	OUTCOME_6M
2	49	2	1	1	2	0	2	2	2	2	2		11	2	0	1	1	2,6	2	1	2016-07-19 20:33	2	2	2	2	3		2	1	1	2		23.3	2	2	1		1	2	1
2	83	1	2	2	2	0	2	2	2	2	2		55	2	1	0	1	1	1	1	2016-09-18 19:15	2	2	2	2	3	1	1	2	2	2		11.7	2	2	2	2	1	2	1
1	61	1	1	2	2	0	2	1	2	2	2		23	1		0	1	1	1	1	2016-09-16 19:30	2	2	2	2	3		2	2	2	2		3.4	1	2	2	1	1	2	1
2	31	1	2	1	2	0	2	2	2	2	2		31	2		0	1	1	1	1	2016-09-19 06:27	2	2	2	2	3	0	1	1	1	2		9	2	1	2	2	1	2	1
1	38	2	1	1	1	0	2	2	2	2	2		27	1	0	0	1	1	1	1	2016-10-11 13:23	2	2	1	2	1	0	2	1	1	2		10.2	2	2	1		0	1	0
2	33	1	2	2	2	0	2	2	2	2	2		46	2		0	2	4	4	1	2016-09-03 12:51	2	2	2	2	3	0	1	1	1	2		27.8	2	2	2	2	1	2	1
1	38	2	2	1	2	0	2	2	2	2	2		20	2	1	0	1	1	1	1	2016-09-10 17:25	2	2	2	2	3	1	1	1	2	1		32	2	2	1		1	2	1
1	57	2	2	2	2		2	2	2	2	2		73	2	0	0	1	1	1	1	2016-09-26 19:58	2	2	2	2	3	0	2	2	2	2		15.9	2	2	1		1	1	
2	37	2	2	2	2	0	2	1	2	2	2		58	1	1	0	1	1	1	1	2016-11-24 00:45	2	2	1	2	1	0	2	1	1	2		42.6	2	2	1		1	2	1
2	64	2	1	1	1	0	1	1	1	1	2	1	48	1	0	0	1	1	1	1	2017-05-28 16:00	2	2	1	2	1	1	2	2	2	2		20	2	2	1		0	1	0
2	41	1	1	1	2	0	2	2	2	2	2		65	2	0	1	1	1	1	1	2018-05-13 06:45	2	2	2	2	3		1	1	2	1		6.9	1	1	2	3	1	2	1
2	71	2	1	2	2	0	1	1	1	1	1		19	1	0	0	1	1	1	1	2018-01-02 02:02	2	1	2	1	2	1	2	1	1	2		24.1	1	2	1		0	1	0
2	67	1	2	1	2	0	1	1	1	2	2		73	2		0	1	1	1	1	2018-07-05 02:36	2	2	2	2	3	1	2	2	2	2		34	1	2	2	3	1	2	1
1	59	1	2	2	2	0	2	1	2	2	2		61	2	0	0	1	2	2	1	2018-12-11 19:13	2	2	2	2	3		1	1	1	2		19.1	2	2	1		1	2	1
1	63	1	1	1	1	0	2	2	2	2	2		73	1	1	0	1	1,2,6	1	1	2015-10-05 07:05	1	2	1	2	1	1	2	1	1	2		9.3	1	2	1		0	1	0
1	50	2	1	1	2	0	2	2	2	2	2		20	2	0	0	1	1	1	1	2015-10-02 10:00	2	2	2	2	3		2	2	2	2		19.8	2	1	1		1	2	1
1	77	2	1	1	2	0	2	2	2	2	2		17	2	0	0	1	1	1	1	2015-11-08 01:15	2	2	2	2	3		2	1	1	2		13.6	2	2	1	4	1	2	1
2	72	1	1	2	2	0	1	1	1	2	1	1	22	1	0	0	1	1,5,6	1	1	2015-11-14 22:00	2	2	2	2	3		2	1	1	2		15.5	1	1	1		1	1	1
1	52	2	1	1	1	0	2	1	2	2	2		98	1	0	0	1	1,9	1,9	1	2015-11-06 21:00	1	2	1	2	1	0	1	1	1	2		34.8	1	2	1		0	1	0
1	82	2	1	2	2	0	2	1	1	2	2		11	1	0	0	1	1,6	1	1	2015-11-21 18:23	2	2	2	2	3	0	2	1	1	2		14.2	1	2	1		1	2	1
2	23	2	1	1	1	0	2	2	2	2	2		19	1	0	0	1	1,6	1	1	2015-12-08 13:35	2	2	2	2	3	0	2	1	1	2		24	2	2	1		0	1	0
2	59	1	1	1	2	0	1	1	1	2	1	1	53	2	0	0	1	1,9	1,9	1	2015-12-15 10:00	1	2	2	2	3		2	1	1	2		27.1	1	2	2	4	1	2	1
2	73	1	1	2	2	0	1	1	1	2	1	1	56	2	0	0	1	1	1	1	2015-12-17 15:00	2	2	2	2	3		2	1	1	2		38.6	1	2	1		1	2	1
1	72	2	1	1	2	0	2	1	2	2	2		5	2		0	1	2	2	1	2016-05-07 15:00	2	2	2	2	3	0	2	1	1	2		10.4	2	2	1		1	2	1
1	30	2	1	2	2	0	2	2	2	2	2		22	1	0	0	1	2	2	1	2016-05-07 21:00	2	2	2	2	3	1	2	1	1	2		16.2	2	2	1		0	1	0
1	35	1	1	1	2	0	2	2	2	2	2		5	2	1	0	1	1	1	1	2016-05-23 12:20	2	2	2	2	3		2	1	1	2		21.4	2	2	1		0	1	0
1	78	1	1	2	2	0	2	2	2	2	2		54	1	0	0	1	1		1	2016-04-26 14:20	2	2	2	2	3		2	2	2	2		0.2	2	1	2	1	1	2	1
2	31	1	1	1	2	0	2	2	2	2	2		9	2	0	0	1	2	2	1	2016-05-28 05:00	2	2	2	2	3		2	1	1	2		6.4	2	2	1		0	1	0
2	62	2	1	1	2	0	2	2	2	2	2		11	2	0	0	1	2	2	1	2016-06-24 12:00	2	2	2	2	3		2	1	1	2		13.1	2	2	2	4	1	2	1
1	67	2	2	2	1	0	2	2	2	2	2		51	1	1	0	1	1	1	1	2016-09-23 14:15	1	2	2	1	2	0	2	2	2	2		26.5	1	2	2	2	1	2	1
1	58	1	1	1	1	0	1	2	2	2	2		25	1	1	0	1	2	1	1	2016-10-01 07:00	1	2	1	2	1	0	2	1	1	2			2	2	1		0	1	0
1	70	2	1	2	2	0	1	1	1	2	2		39	2	0	0	1	1	1	1	2016-12-10 19:00	2	2	2	2	3	0	2	1	1	2		26.1	2	2	1		1	2	1
1	71	2	1	1	1	0	2	1	1	2	2		46	1	1	0	1	1	1	1	2017-01-27 12:00	1	2	2	1	2	1	2	1	2	2		6.5	1	2	2	1	1	2	1
1	67	1	2	1	2	0	2	2	2	1	2		26	2	1	0	1	1	1	1	2017-02-10 13:20	2	2	2	2	3		2	2	2	2		16	2	2	1		1	2	1
1	63	1	1	1	2	0	2	1	2	2	2		30	1	0	0	1	1	1	1	2016-11-21 08:21	2	2	2	2	3	1	2	1	1	2		11.5	2	2	1		1	2	1
2	34	2	1	1	1	1	2	1	2	2	2		59	1	1	1	1	1	1	1	2016-12-15 20:00	2	2	2	2	3		2	2	2	2		25.7	1	2	2	2	1	2	1
1	61	2	1	2	2	1	2	2	1	2	2		9	1	0	0	1	1,6	1	1	2017-02-20 17:00	1	2	2	1	2	0	2	2	2	2			1	2	1		1	1	0
2	45	1	2	2	2	0	2	2	2	2	2		33	2	0	0	1	1	1	1	2017-02-21 16:00	2	2	2	2	3		2	2	2	2		13.4	2	2	1		1	2	1
2	70	1	2	1	2	0	2	1	1	2	2		59	1	0	0	1	1	1	1	2017-03-02 16:45	2	2	2	2	3		1	2	2	2		12.3	1	2	2	2	1	2	1
2	77	1	1	1	2	0	2	2	2	2	2		76	2	0	0	1	1	1	1	2017-03-26 12:00	2	2	1	2	1	0	1	2	2	2		28.9	2	2	2	4	1	2	1
1	53	2	1	1	1	0	2	2	2	2	2		6	1	0	0	1	2	2	1	2017-03-31 19:30	2	2	2	2	3	0	2	2	2	2			2	2	1		0	1	0
1	36	2	1	1	1	0	2	2	2	2	2		5	1	0	1	1	1	1	1	2017-04-16 20:40	2	2	2	2	3	0	1	1	2	1		29.7	2	2	1		0	1	0
1	61	2	1	1	2	0	2	2	2	1	2		0	2		0	1	1	1	1	2017-04-16 05:00	2	2	2	2	3		2	1	1	2		33.9	2	2	1		1	2	1
1	69	2	2	1	1	1	2	1	1	1	2		75	1	1	0	1	1	1	1	2017-05-02 18:50	1	2	2	1	2	0	2	2	2	2		29.7	2	2	2	1	1	2	1
1	57	2	1	1	1	0	2	2	2	2	2		42	1	0	0	1	1	1	1	2017-05-03 19:40	2	2	2	2	3	0	2	1	1	2		15.5	2	2	1		1	1	1
1	54	2	1	1	2	0	2	2	1	2	2		4	1	0	0	1	2	1	1	2017-05-22 18:50	2	2	2	2	3	0	1	1	1	2		33.1	2	2	1		1	2	1
1	27	1	1	2	2	1	2	2	2	2	2		74	2		0	1	1	1	1	2017-05-22 16:45	2	2	2	2	3		2	1	1	2		70.9	1	2	2	2	1	2	1
2	63	2	2	1	2	0	1	1	2	2	2		34	1	0	0	1	1	1	1	2017-06-10 14:15	2	2	2	2	3		2	1	1	2		21.5	2	2	2	2	1	2	1
2	83	1	1	2	2	1	2	2	2	2	2		31	2	0	0	1	1	1	1	2017-06-12 20:10	2	2	2	2	3		2	1	1	2		23	2	2	2	2	1	2	1
2	63	1	1	2	2	0	2	2	2	2	2		44	1	0	0	1	1	1	1	2017-06-07 18:25	2	2	2	2	3	0	2	1	1	2		42.6	2	2	1		1	2	1
1	33	2	1	1	2	1	2	1	2	2	2		4	2	0	0	1	2	2	1	2017-06-20 17:48	2	2	2	2	3		2	1	1	2		8.7	2	2	1		0	1	0
2	70	2	1	1	2	0	2	1	2	2	2		23	2	0	0	1	1	1	1	2017-06-21 15:00	2	2	2	2	3		2	1	1	2		13.2	2	2	1		1	1	1
1	55	2	1	1	2	0	2	2	1	2	2		35	2	0	0	1	2,6	1	1	2017-06-23 11:10	2	2	2	2	3		2	1	2	1		3.8	1	2	2	3	1	2	1
1	39	2	1	1	2	0	2	2	2	2	1		21	2	0	0	1	2	2	1	2017-07-10 13:25	2	2	2	2	3		2	1	1	2		29	1	2	1		1	2	1
2	74	1	1	1	2	1	2	2	2	2	2		33	2	0	0	1	1	1	1	2017-07-13 08:30	2	2	2	2	3		1	1	2	1		4	1	2	1		1	1	1
2	36	1	2	1	2	0	2	2	2	2	2		51	2		1	1	2,5	2,5	1	2017-08-14 08:15	2	2	2	2	3		2	1	1	2		21.8	2	2	2	2	1	2	1
1	61	2	1	1	1	0	2	2	2	2	2		37	1	0	0	1	2	2	1	2017-09-05 13:30	2	2	1	2	1	0	2	1	1	2		15.9	2	2	1	4	0	1	0
1	75	2	1	1	1	0	2	1	2	2	2		27	1	0	0	1	1	1	1	2017-09-11 17:00	2	2	1	2	1		2	2	2	2		15.5	2	2	2	1	1	2	1
2	58	1	2	2	2	0	2	2	2	2	2		17	1	1	0	1	1	1	1	2017-09-19 03:27	2	2	2	2	3		2	2	2	2		36.9	2	2	1		1	2	1
1	48	2	1	2	1	0	2	2	2	2	2		40	1	1	0	1	1	1	1	2017-09-21 01:25	2	2	2	1	2	1	2	2	2	2		23.4	2	2	1		0	1	0
1	37	2	1	1	1	0	2	2	2	2	2		5	1	0	0	1	1	1	1	2017-09-28 19:00	2	2	2	2	3	0	2	1	1	2		10	2	2	1		0	1	0
1	83	2	2	1	1	0	2	1	2	2	2		45	1	0	0	1	1	1	1	2017-10-10 21:00	2	2	2	1	2		2	2	2	2		0.2	2	1	2	1	1	2	1
2	59	2	1	1	2	0	2	2	2	2	2		1	1	0	0	1	1,6	1	1	2017-10-07 17:48	1	1	1	2	1	1	2	1	1	2		19	1	1	1		0	1	0
2	52	1	1	1	1	1	2	2	2	2	2		2	1	0	1	1	1	1	1	2017-10-21 10:39	2	2	2	1	2		2	1	1	2		17.7	2	2	1		0	1	0
1	64	2	1	2	2	0	2	1	2	2	2		6	2	0	0	1	1	1	1	2017-10-22 16:00	2	2	2	2	3		2	2	2	2		15.2	2	2	1		0	1	0
1	62	1	1	1	2	0	2	1	2	2	2		55	2	0	1	1	1	1	1	2017-11-12 19:00	2	2	2	2	3		1	2	2	2		16.7	2	2	2	4	1	2	1
1	60	2	2	2	1	0	2	2	2	2	2		13	1	0	0	1	1	1	1	2017-11-24 09:00	2	2	2	1	2		2	1	1	2		15.8	2	2	1		0	1	0
2	64	2	1	1	2	0	2	2	2	2	2		12	2	0	0	1	1	1	1	2017-11-24 13:40	2	2	2	2	3		2	2	2	2		12.2	2	2	1		1	1	1
1	79	2	1	1	2	0	2	1	2	2	2		40	1	0	0	1	1	1	1	2017-11-27 16:00	2	2	2	2	3		2	2	2	2		24.5	1	2	1		1	2	1
1	55	1	1	1	1	0	2	2	2	2	2		22	1	0	0	1	1	1	1	2017-12-19 16:00	2	2	1	2	1	0	2	1	1	2		15.7	2	2	1		0	1	1
1	64	2	1	1	1	0	2	2	2	2	2		40	1	0	0	1	2	2	1	2017-12-31 13:50	2	2	2	1	2		2	1	1	2		17	2	2	1		1	1	1
2	50	1	2	1	2	0	2	2	2	2	2		24	2	0	0	1	1	1	1	2018-01-28 16:40	2	2	2	2	3		2	1	1	2		31.7	2	2	1		1	2	1
1	60	1	1	1	1	0	1	2	2	2	2		55	1	0	0	1	1	1	1	2018-01-19 11:20	2	2	2	2	3	0	2	2	2	2		1.6	2	1	2	1	1	2	1
1	63	2	1	1	1	0	2	1	2	2	2		42	1	0	0	1	1,5	1,5	1	2018-02-12 17:35	2	2	2	1	2	1	2	1	1	2		34	2	2	1		1	2	1
1	63	1	2	1	2	0	2	2	2	2	2		7	2	1	0	1	1,5	1,5	1	2018-02-15 15:30	2	2	2	2	3		2	1	1	2		19.8	2	2	1		1	1	1
1	52	1	1	1	1	0	2	2	2	2	2		17	1		0	1	1	1	1	2018-03-05 16:40	2	2	2	2	3		2	1	1	2		13.3	2	2	1		0	1	0
1	50	1	1	2	1	0	1	2	2	2	2		24	1		0	1	1	1	1	2018-03-17 15:40	2	1	1	2	1	1	2	1	1	2		17	2	1	1		0	1	0
2	32	1	2	1	2	0	2	2	2	2	2		31	2	0	0	1	1	1	1	2018-03-23 16:00	2	2	2	2	3		2	1	1	2		16.2	2	2	2	2	1	2	1
1	46	2	1	1	2	0	2	2	2	2	2		41	2	0	0	1	1	1	1	2018-03-24 14:25	2	2	2	2	3		2	1	1	2		24.2	2	2	2	2	1	2	1
2	68	2	2	2	2	0	2	2	2	2	2		39	2	0	0	1	1	1	1	2018-03-27 16:55	2	2	2	2	3		2	2	2	2		20.3	2	2	2	2	1	2	1
1	80	2	2	2	1	0	1	1	2	2	2		7	1	0	0	1	1	1	1	2018-04-01 13:10	2	2	2	2	3		2	1	1	2		6.1	2	2	1		0	1	0
2	30	2	1	2	1	0	2	2	2	2	2		11	1	0	0	1	1	1	1	2018-04-17 16:10	2	2	2	2	3	0	2	2	2	2		5.1	2	2	1		0	1	0
1	83	2	1	2	1	0	2	2	2	2	2		19	1	0	0	1	1,5,6	1,5,6	1	2018-04-29 17:50	2	2	2	1	2	1	2	1	2	1		11.4	2	2	1		0	1	0
2	77	1	2	1	2	0	2	2	2	2	2		49	2	0	1	1	2	2	1	2018-05-04 16:15	2	2	2	2	3	1	2	1	2	1		25.7	2	2	2	2	1	2	1
1	85	1	2	2	1	0	2	2	2	2	2		94	1	0	1	1	2	2	1	2018-04-09 15:05	2	1	2	2	3	0	2	2	2	2		8.1	1	2	2	1	1	2	1
1	62	2	1	1	1	0	1	2	2	2	2		14	1	1	0	1	1	1	1	2018-05-27 06:00	2	2	1	2	1	0	2	2	2	2		30.8	2	2	1		0	1	0
2	67	2	1	1	1	0	2	1	2	2	2		27	1	0	0	1	1	1	1	2018-05-29 13:00	2	2	2	1	2	0	2	1	2	1		31	2	2	1		0	1	0
1	79	2	1	2	2	0	1	1	1	2	2		38	1	0	0	1	1	1	1	2018-06-05 16:40	2	2	2	2	3		2	1	2	1		15.8	2	2	1		1	2	1
2	85	1	2	1	2	0	1	1	1	2	2	1	54	2	0	1	1	1	1	1	2018-06-17 21:45	2	2	2	2	3		2	1	2	1		4.3	1	2	2	4	1	2	1
2	87	2	2	1	2	0	2	2	2	2	2		25	2	0	0	1	2	2	1	2018-06-27 18:00	2	2	2	2	3		2	1	1	2		14.2	2	2	1		1	2	1
1	84	2	1	1	1	0	1	2	2	1	2		7	1	0	1	1	2	2	1	2018-07-05 14:00	2	2	2	2	3	1	2	2	2	2		14.8	2	2	2	1	1	2	1
2	45	1	2	2	2	0	2	2	2	2	2		77	2	0	0	1	2,6	2	1	2018-07-13 23:30	2	2	2	2	3	1	2	1	1	2		7.1	2	2	2	2	1	2	1
2	47	1	2	2	2	0	2	2	2	2	2		30	2	1	0	1	1	1	1	2018-08-04 17:00	2	2	2	2	3		2	2	2	2		0.1	1	1	2	1	1	2	1

1	52	1	1	1	1	0	2	2	2	2	2		10	1	0	0	1	1	1	1	2018-08-09 11:21	2	2	1	2	1	0	2	1	1	2		18.6	2	2	1		0	1	0
1	49	2	1	1	1	0	2	2	2	1	2		33	1	1	0	1	1,5,6	1	1	2018-08-11 16:40	2	2	2	2	3	1	2	1	1	2		19.3	2	2	2	2	1	2	1
2	33	2	1	2	1	0	2	2	2	2	2		25	1	0	0	1	1,5	1	1	2018-08-18 18:50	2	2	1	2	1	0	2	1	1	2		14.8	2	2	1		0	1	0
2	56	2	2	1	1	0	2	2	2	2	2		21	1	0	0	1	1	1	1	2018-08-24 21:40	2	2	2	2	3	0	2	1	1	2		25.5	2	2	1		0	1	0
1	78	2	2	1	2	0	2	2	2	2	2		38	2	0	0	1	1	1	1	2018-08-25 19:45	2	2	2	2	3	0	2	1	1	2		16.1	2	2	1		1	1	1
2	55	2	1	2	2	0	2	1	1	1	1		4	2	0	0	1	1	1	1	2018-09-17 14:55	2	2	2	2	3		2	1	1	2		14.1	1	2	1		0	1	0
1	66	2	1	2	2	0	2	2	2	2	2		7	2	1	1	1	1	1	1	2018-09-18 18:00	2	2	2	2	3		1	1	2	1		26.2	1	2	2	3	1	2	1
1	71	1	1	1	2	0	1	1	1	2	2		8	1	0	0	1	1	1	1	2018-10-14 18:15	2	2	2	1	2	1	2	1	1	2		33.7	2	2	2	2	1	2	1
2	81	2	1	1	2	0	1	1	1	2	2		23	1	0	0	1	1		1	2018-10-15 14:45	2	2	2	1	2		2	2	2	2		0.1	2	1	2	1	1	2	1
1	66	2	1	1	2	0	1	1	1	2	1	1	21	1	0	0	1	1	1,5	1	2018-10-20 17:40	2	2	1	2	1		2	2	2	2		33.1	1	2	1		0	2	1
1	58	1	1	2	1	0	2	1	2	2	2		118	1		1	1	1	1	1	2018-09-28 11:40	1	2	2	1	2	1	2	2	2	2		14.5	1	1	2	1	1	2	1
1	76	2	1	1	1	0	2	1	1	2	2		15	1	0	1	1	2	2	1	2018-10-15 20:20	2	2	2	2	3		2	2	2	2		23.2	1	2	1		0	1	0
1	81	2	1	1	2	0	2	1	2	2	2		38	1	0	0	1	1	1	1	2018-10-28 19:00	2	2	2	2	3	1	2	2	2	2		10.8	1	2	2	2	1	2	1
1	78	2	1	1	2	0	2	1	2	2	1		37	1	0	0	1	1	1	1	2018-11-05 19:50	2	2	2	2	3	0	2	1	1	2		8.1	1	2	2	4	1	2	1
2	80	1	1	1	2	1	1	1	2	2	2		43	2	0	0	1	2	2	1	2018-11-29 17:32	2	2	2	2	3		2	1	1	2		35.4	2	2	2	4	1	2	1
2	60	1	1	2	2	0	1	2	2	2	2		15	1		0	1	2	2	1	2018-12-04 15:08	2	2	2	2	3	0	2	2	2	2		13.6	2	2	1		0	1	0
1	54	2	2	2	2	1	2	2	1	2	1		11	2	0	0	1	2	2	1	2018-12-23 18:28	2	2	2	2	3		2	2	2	2		31.8	1	2	2	3	1	2	1
1	90	1	2	2	1	0	1	2	2	2	2	1	8	2	0	0	1	1	1	1	2018-12-21 19:45	2	2	2	2	3		2	1	1	2		26.8	2	2	1		1	1	1
1	69	1	1	1	2	0	2	1	1	2	1		19	1	0	0	1	2	2	1	2018-12-31 23:00	2	2	2	1	2		2	1	1	2		27	1	2	1		1	1	1
1	72	1	1	2	1	0	2	2	2	2	2		42	1	0	0	1	1	1	1	2015-10-08 17:26	2	2	2	1	2		2	1	2	1		33	2	2	1		1	2	1
2	46	2	1	2	1	0	1	2	2	2	2		14	1	0	0	1	1	1	1	2015-10-23 03:15	2	2	1	2	1	0	2	2	2	2		7.8	2	2	1		0	1	0
1	58	2	1	1	1	0	1	2	2	2	2		29	1	0	0	1	1	1	1	2015-10-30 20:49	2	2	1	2	1	0	2	1	1	2		13.2	2	2	1		0	1	0
2	76	2	1	1	2	0	2	1	2	2	2		45	2	0	0	1	1	1	1	2015-11-01 19:30	2	2	2	2	3		2	1	1	2		38.9	2	2	1		1	2	1
1	71	1	1	2	2	0	2	1	2	2	2		53	1	1	0	1	1	1	1	2015-11-09 14:21	2	2	2	2	3	0	1	2	2	2		27.6	1	2	2	4	1	2	1
2	41	2	1	2	1	1	2	2	2	2	2		33	1	1	0	1	1,6	1	1	2015-12-05 18:42	2	2	1	2	1	0	2	1	1	2		26.9	2	2	1		1	1	1
1	58	1	2	2	2	0	2	2	2	2	2		20	2	1	0	3	10	10	1	2016-11-08 03:27	2	2	2	2	3		2	1	1	2		22.2	2	2	1		1	2	1
1	49	1	1	2	2		2	2	1	2	2		49	1	1	0	1	1	1	1	2016-11-20 20:07	1	2	1	2	1	0	2	1	1	2		38	1	2	2	1	1	2	1
1	83	2	2	2	2	0	2	1	2	2	2		29	2	0	0	1	1	1	1	2016-11-23 17:21	2	2	2	2	3	0	2	2	2	2		11.1	2	2	1		1	2	1
2	70	1	2	2	2	0	1	1	1	2	2		38	1	0	0	1	1	1	1	2017-02-06 23:31	2	2	2	2	3	0	2	1	1	2		22.7	1	2	1		1	2	1
1	76	2	1	1	2	0	2	2	2	2	2		13	2	0	0	1	1	1	1	2017-03-03 16:37	2	2	2	2	3	0	2	1	1	2		10.7	2	2	1		1	1	1
1	56	1	1	1	2	1	2	1	1	2	2		62	1	0	0	1	1	1	1	2017-03-20 19:12	2	2	1	2	1		2	2	2	2		11.3	2	2	2	3	1	2	1
1	60	2	1	1	1	0	2	2	2	2	2		20	1	0	0	1	1	1	1	2017-03-21 14:55	2	2	2	1	2	0	2	2	2	2		23.7	2	2	1		0	1	0
1	76	1	1	2	1	0	2	1	1	2	2		13	1	0	0	1	1	1	1	2017-04-11 03:40	2	2	2	1	2		2	1	1	2		16.5	2	2	1		0	1	0
1	50	1	1	1	2	0	2	2	2	2	2		27	2	1	0	1	1	1	1	2017-04-15 04:10	2	2	2	2	3		2	2	2	2		25.4	2	2	2	2	1	2	1
1	31	2	1	2	1	0	1	2	2	2	2		20	1	0	0	1	1	1	1	2017-04-27 20:15	2	2	2	1	2	0	2	2	2	2		28.7	2	2	1		0	1	0
1	65	1	2	2	1	0	2	2	2	2	2		37	1		0	1	1	1	1	2017-05-01 14:20	2	2	2	1	2		2	1	1	2		10.8	2	2	2	2	1	2	1
2	70	1	2	2	2	1	2	1	2	2	2		20	2		0	1	1	1	1	2017-05-02 16:38	2	2	2	2	3		2	2	2	2		16.5	2	2	2	2	1	2	1
1	54	2	2	2	2	0	2	2	1	2	2		25	2	1	0	1	1	1	1	2017-05-13 03:38	2	2	2	2	3	0	2	1	2	1		25.9	1	2	2	2	1	2	1
1	77	1	1	1	2	0	2	1	2	2	2		24	1	1	0	1	1	1	1	2017-05-29 16:00	2	2	2	2	3	0	2	2	2	2		36.1	1	2	2	2	1	2	1
2	47	2	2	1	2	0	2	2	2	2	2		63	2	1	0	1	1	1	1	2017-06-21 01:35	2	2	2	2	3	0	2	2	2	2		31.3	2	2	1		1	2	1
1	58	2	2	2	1	0	2	2	2	2	2		7	1	0	0	1	1	1	1	2017-07-08 17:22	1	2	2	1	2		2	2	2	2		16.2	2	2	1		0	1	0
1	68	2	1	1	1	0	2	1	1	2	2		30	1	0	0	1	1	1	1	2017-07-24 08:45	2	2	1	2	1	0	2	2	2	2		25.1	2	2	1		1	1	1
1	58	1	1	2	2	0	2	2	2	2	2		12	2	0	0	1	1	1	1	2017-08-04 16:14	2	2	2	2	3		2	2	2	2		6.6	2	2	1		0	1	0
1	41	2	1	1	1	0	2	1	2	2	2		3	1	0	0	1	1	1	1	2017-09-07 15:00	2	2	2	1	2	0	2	2	2	2		16.6	2	2	1		0	1	0
1	39	2	1	2	1	0	2	2	2	2	2		34	1	0	0	1	1	1	1	2017-09-16 11:30	2	2	2	1	2		2	2	2	2		25.1	2	2	2	1	1	2	1
2	41	1	2	1	2	0	2	2	2	2	2		31	2	0	0	1	1	1	1	2017-09-30 02:46	2	2	2	2	3	0	2	1	1	2		20.9	2	2	1		1	2	1
1	62	2	1	1	1	0	2	1	2	2	2		13	1	0	0	1	1	1	1	2017-12-03 21:21	2	2	1	2	1	0	2	2	2	2		22.3	1	2	1		0	1	0
2	61	1	1	1	2	1	2	1	1	2	2		45	2	0	0	1	1	1	1	2017-12-04 19:15	2	2	1	2	1	0	2	2	2	2		37.8	1	1	2	4	1	2	1
2	50	1	1	1	2	0	2	1	2	2	2		26	1	0	0	1	1	1	1	2018-01-21 03:13	2	2	2	2	3	0	2	1	1	2		29.3	1	2	1		1	2	1
1	63	2	2	2	1	0	2	2	2	2	2		28	1	1	0	1	1	1	1	2018-02-27 04:53	1	2	1	2	1		2	2	2	2		25.2	2	1	2	1	1	2	1
1	71	2	1	1	2	0	2	1	2	2	2		106	1	0	0	1	1	1	1	2018-03-13 10:29	1	2	2	1	2	1	2	2	2	2		16.8	1	1	2	1	1	2	1
1	75	2	1	1	2	0	2	1	1	2	2		10	2	1	1	1	1	1	1	2018-04-02 10:37	2	2	2	2	3	1	2	1	1	2		8.4	2	2	1		1	1	1
2	52	1	2	2	2	0	2	2	2	2	2		15	1	0	0	1	1	1	1	2018-04-08 16:45	2	2	1	2	1	0	2	2	2	2		7.4	2	2	1	4	0	1	0
1	55	2	1	1	2	0	2	2	2	2	2		28	2	1	0	1	1	1	1	2018-04-22 19:06	2	2	2	2	3		2	1	1	2		31.1	2	2	1		1	1	1
1	55	1	1	1	1	0	2	2	2	2	2		12	1	0	0	1	1	1	1	2018-06-17 07:14	2	2	1	2	1	0	2	2	2	2		37.9	2	2	1		0	1	0
1	50	2	1	1	1	0	2	2	2	2	2		26	1	0	1	1	1	1	1	2018-08-10 18:00	2	2	2	1	2	0	2	2	2	2		17.8	2	2	1	4	0	1	0
1	36	1	1	2	2	1	2	2	2	2	2		58	1	1	0	1	1	1	1	2018-08-19 06:01	2	2	1	2	1	0	2	2	2	2		37.8	2	2	2	2	1	2	1
1	58	1	1	1	2	0	2	2	1	2	2		11	2	0	0	1	1	1	1	2018-08-19 17:52	2	2	2	2	3	1	2	2	2	2		30.1	2	2	1		1	1	1
1	44	2	1	1	2	1	2	2	2	2	2		22	1	0	1	1	1	1	1	2018-09-23 18:03	2	2	1	2	1	1	2	2	2	2		24.7	2	2	1		0	1	0
1	63	1	1	1	1	0	1	2	2	2	2		32	1	0	0	1	1	1	1	2018-09-24 08:39	2	2	2	2	3		2	2	2	2		14.8	1	1	2	1	1	2	1
2	82	1	1	1	1	0	2	2	2	2	2		34	1	0	0	1	1	1	1	2018-11-19 16:23	2	2	1	2	1		2	2	2	2		33.3	2	2	1		1	1	1
1	57	1	1	1	1	0	2	2	2	2	2		41	1	0	0	1	1	1	1	2018-11-27 09:10	2	2	2	1	2	0	2	2	2	2		12.9	2	2	1		0	1	0
2	72	1	1	1	2		2	2	2	2	2		32	2	0	1	1	1	1	1	2018-11-28 12:53	2	2	2	2	3		2	2	2	2			1	2	1		1	1	
2	62	2	1	1	1	0	2	2	2	2	2		24	1	0	0	2	4,6	4	1	2016-01-13 16:40	2	2	1	2	1	0	2	1	1	2		15.2	2	2	1		0	1	0
2	85	1	1	2	2	0	2	1	2	1	2		29	1	0	0	2	4	4	1	2016-01-22 17:30	2	2	2	2	3	0	2	1	1	2		79.2	2	2	1		1	1	1
1	73	2	1	1	2	0	2	1	1	2	2		11	1	0	1	1	2,5	2,5	1	2016-07-01 08:00	2	2	2	2	3		2	2	2	2		35.3	2	2	2	1	1	2	1
1	43	1	2	1	1	0	2	2	2	2	2		18	1	0	0	2	4	4	1	2016-06-24 05:15	2	2	2	2	3	0	2	1	1	2		13.6	2	2	1		0	1	0
1	65	2	1	2	1	0	1	1	2	2	2		13	1	0	0	2	4	4	1	2016-11-07 09:05	2	2	1	2	1	0	2	1	1	2		14.9	2	2	1		0	1	0
2	68	1	2	2	2	0	2	2	2	2	2		38	2	0	0	2	4	4	1	2016-10-17 02:50	2	2	2	2	3		2	1	1	2		2.8	2	1	2	3	1	2	1
1	78	1	1	1	2	0	2	2	2	2	2		41	1	0	0	2	4	4	1	2016-09-21 15:10	2	2	2	2	3		2	1	1	2		27.1	2	2	2	1	1	2	1
2	48	2	1	1	2	0	2	1	2	2	2		18	1	1	0	2	4	4	1	2016-06-30 13:50	2	2	2	2	3	0	2	2	2	2		29.5	1	2	2	2	1	2	1
2	67	2	1	1	2	0	1	1	2	2	2		5	1		0	2	4	4	1	2016-01-29 20:00	2	2	1	2	1	0	2	1	1	2		73.2	2	2	1		0	1	0
2	63	1	2	1	2	0	2	1	2	2	2		28	1	0	0	2	4	4	1	2016-12-08 11:50	2	2	2	2	3	0	2	1	1	2		8.9	2	2	1		1	1	1
1	25	2	1	2	1	0	2	2	2	2	2		2	1	0	0	2	4	4	1	2017-01-21 11:10	2	2	2	2	3	0	2	1	1	2		17	2	2	1		0	1	0
1	52	1	2	2	2	0	2	2	2	2	2		35	2	0	0	2	4	4	1	2017-02-20 00:52	2	2	2	2	3		2	2	2	2		1.9	2	1	2	4	1	2	1
1	57	2	1	1	2	0	2	2	1	2	2		32	1	0	1	2	4	4	1	2017-05-07 15:00	2	2	2	2	3	1	2	1	1	2		25.2	2	2	2	4	1	2	1
1	67	1	1	2	2	0	2	1	2	2	2		38	1	0	0	2	4	4	1	2017-05-30 13:30	2	2	2	2	3	0	2	1	1	1		41.1	1	2	2	3	1	2	1
2	44	2	2	2	1	0	2	2	2	2	2		34	1	0	0	2	4	4	1	2017-07-10 16:00	2	2	2	2	3	1	2	1	1	2		13.3	2	2	2	2	1	2	1
1	27	1	2	1	2	0	2	2	2	2	2		23	2	1	0	2	4	4	1	2017-12-19 05:10	2	2	2	2	3	1	2	1	2	2		51.2	2	2	2	2	1	2	1
1	74	2	2	2	2	0	2	1	1	2	2		9	2	0	0	1	2	2	1	2018-03-27 22:30	2	2	2	2	3	1	2	2	2	2		100	2	1	2	3	1	2	1
1	56	1	2	2	2	0	2	1	1	2	2		37	2	0	0	2	4	4	1	2018-04-08 06:20	2	2	2	2	3	0	2	1	1	2		100	2	2	2	3	1	2	1
2	54	1	2	1	1	0	2	2	2	2	2		82	1	0	0	1	2	2	1	2018-04-23 08:50	2	2	1	2	1	0	2	2	2	2		100	1	1	2	3	1	2	1
2	20	2	1	1	1	0	2	2	2	2	2		21	1	0	0	2	4	4	1	2018-04-28 22:50	2	2	2	2	3	0	2	1	1	2		100	2	2	1		0	1	0
1	61	1	2	1	2	0	2	2	2	2	2		33	1	1	0	1	2,6	2	1	2018-04-29 14:30	2	2	2	2	3		2	2	2	2		100	2	1	2	3	1	2	1
1	51	2	1	2	2	0	2	2	2	2	2		34	1	0	1	1	2,5	2,5	1	2018-08-09 23:50	2	2	2	2	3		2	2	2	2		52.8	1	1	2	3	1	2	1
2	21	1	2	1	2	0	2	2	2	2	2		51	2	0	0	2	4	4	1	2018-09-13 07:05	2	2	2	2	3	0	2	2	2	2		100	2	2	2	2	1	2	1
1	59	2	1	1	1	0	2	1	2	2	2		15	1	0	1	2	4	4	1	2018-11-07 13:05	2	2	1	2	1		1	2	2	2		100	2	2	1		0	1	0
1	46	2	2	2	2	0	2	2	2	2	2		9	2	0	0	2	4,5,6	4	1	2018-11-28 16:55	2	1	2	2	3	0	2	1	2	2		100	2	2	1		1	2	1
1	75	1	1	2	2	0	2	2	2	2	2		48	1	0	0	2	4	4	1	2018-10-15 05:40	2	2	2	2	3	0	2	1	1	2		100	1	2	1		1	1	1
1	85	2	2	2	2	0	1	1	1	2	1		20	1	0	0	1	1	1	1	2015-11-03 14:20	2	2	2	1	2		2	1	1	2		18.4	2	2	2	1	1	2	1
1	46	2	1	2	2	1	2	1	1	2	2		23	2	0	0	1	2,5,6	2	1	2015-11-10 19:00	2	2	2	2	3		2	2	2	2		34.1	2	2	2	3	1	2	1
1	58	1	1	2	2	0	2	2	1	2	1		44	2	0	0	1	1	1	1	2015-11-16 01:16	2	2	2	2	3		2	1	1	2		24.1	1	2	2	2	1	2	1
2	71	2	1	2	2	0	2	1	2	2	2		36	1	0	0	1	1,6,10	1	1	2015-11-27 03:50	2	2	1	2	1		2	1	1	2		25.4	2	2	1		1	2	1
1	42	2	1	1	1	0	2	1	2	2	2		8	1	0	0	2	4	4	1	2015-12-02 13:30	2	2	2	2	3	0	2	2	2	2		9.6	2	1	2	2	1	2	1
1	55	2	1	1	1		2	2	1	2	2		58	1	0	0	2	4,6	4	2		2	2	2	2	3		2	1	1	1		16.4	2	1	1		1		
1	72	1	2	1	2	0	1	1	1	2	2		29	1	0	0	1	1,10	1	1	2015-12-14 18:00	2	2	2	2	3	1	2	2	2	2		17.2	2	2	2	1	1	2	1
1	71	2	1	1	2	0	2	2	1	1	2		33	1	0	0	1	1	1	1	2016-01-03 20:50	2	2	2	2	3	1	2	1	1	2		24.3	2	2	2	2	1	2	1
1	72	2	1	1	1	0	2	2	2	2	2		7	1	0	0	1	1	1	1	2016-01-09 04:00	2	2	2	1	2	1	2	2	2	2		0.1	2	2	1		0	1	0
2	28	1	2	1	2	0	2	2	2	2	2		20	2	0	0	2	4	4	1	2016-01-09 04:57	2	2	2	2	3		2	2	2	2		11.4	2	2	1		1	2	1
2	40	1	1	2	2	1	1	2	2	2	2		9	1	0	0	1	1	1	2		2	2	1	2	1		2	2	2	2		7.2	2	2	1		0	1	0
1	33	2	1	1	1	0	2	1	2	2	2		19	1	0	0	1	1,5,6	1	1	2016-01-14 20:30	2	2	1	2	1		2	2	2	2		0.1	2	2	1		0	1	0
1	59	1	1	1	2	0	2	2	2	2	2		16	2	0	0	2	4	4	1	2016-02-08 23:00	2	2	2	2	3		2	2	2	2		27.6	2	2	2	2	1	2	1
1	64	1	1	2	2	0	1	2	2	2	2	1	15	1	0	0	2	4	4	1	2016-02-10 15:00	2	2	1	2	1	0	2	2	2	2		0.7	2	1	1		0	1	0
1	46	2	1	1	2	0	2	2	2	2	2		18	2	0	0	2	4	4	1	2016-02-17 03:00	2	2	2	2	3		1	1	1	2		7.6	2	2	1		1	1	1
1	81	1	1	1	1	0	2	1	1	2	2		41	1	0	1	1	1		1	2016-02-21 20:00	2	2	2	1	2	1	2	2	2	2		0.4	2	1	2	1	1	2	1
1	56	1	1	1	2	0	2	1	1	2	2		15	1	0	0	1	1	1	1	2016-03-11 08:45	2	2	2	1	2	1	2	1	1	2		7.8	2	2	1		0	1	0
1	42	2	2	2	2	0	2	2	2	2	2		59	2	0	0	1	1		2		2	2	2	2	3		2	1	1	1		20.8	2	2	2	1	1	2	1
1	58	2	1	1	1	0	2	1	2	2	2		72	1	0	0	2	4,5	4	1	2016-03-24 11:00	2	2	1	2	1		2	1	1	2		11.3	2	1	2	3	1	2	1
1	58	1	2	1	2	0	2	1	1	2	1		52	2	0	0	1	1,5	1	1	2016-04-02 00:00	2	2	2	2	3		2	1	1	2		10.8	1	2	2	3	1	2	1
1	73	1	2	2	2	0	2	2	2	2	2		9	2	0	0	2	4	4	1	2016-05-01 01:30	2	2	2	2	3	0	2	1	1	2		6.9	2	2	1		1	2	1
1	44	2	1	2	1	0	2	2	2	2	2		43	1	1	1	1	1	1	1	2016-05-17 11:14	1	2	2	1	2		2	1	1	2		13.6	2	2	1		0	1	0
1	76	1	1	2	2	0	2	2	1	2	2		37	2	0	0	1	1	1	1	2016-06-08 05:44	2	2	2	2	3		2	1	1	1		33.6	2	2	2			2	1
2	44	1	1	1	1	0	2	2	2	2	2		33	1	0	0	2	4	4	1	2016-06-10 09:45	2	2	1	2	1		1	2	2	2		8.3	2	2	1		0	1	0
1	87	1	1	2	2	0	2	2	2	2	2		39	2	0	0	1	1	1	1	2016-06-17 02:00	2	2	2	2	3		2	1	2	1		18.2	2	2	2	3	1	2	1
2	50	1	2	1	2	0	2	2	2	2	2		34	2	0	0	2	4	4	1	2016-06-17 18:30	2	2	2	2	3		2	2	2	2		28.5	2	2	2	2	1	2	1

2	76	2	1	2	2	0	2	1	2	2	2		38	2	0	1	2	4	4	1	2016-06-17 21:00	2	2	2	2	3		2	1	1	2	34.1	2	2	2	3	1	2	1
1	43	1	2	1	2	0	2	2	2	2	2		36	2	0	0	1	1,6	1	1	2016-06-24 05:35	2	2	2	2	3		2	2	2	2	24.7	2	2	2	2	1	2	1
2	49	1	1	1	2	0	2	2	2	2	2		9	2	0	1	1	1	1	2		2	2	2	2	3		2	2	2	2	15	2	2	1		1	2	1
1	49	1	1	1	1	0	2	2	2	2	2		92	1	1	0	1	1	1	1	2016-07-19 15:45	1	2	1	2	1		2	2	2	2	34.1	1	2	2	1	1	2	1
1	44	2	1	1	2	0	2	2	2	2	2		11	1	1	0	2	4		1	2016-07-21 12:25	2	2	2	2	3		2	1	2	1	4.4	2	2	2	1	1	2	1
1	84	2	1	1	2	0	1	2	2	2	2		69	1	0	0	1	1	1	1	2016-08-16 11:00	1	2	1	2	1		2	2	2	2	28.3	1	2	2	1	1	2	1
1	77	2	1	1	2	0	2	2	1	2	1		17	1	0	0	2	4	4	1	2016-08-20 00:05	2	2	2	2	3		2	2	2	2	14.6	2	2	1		1	2	1
2	86	1	1	1	2	0	2	2	2	2	2		34	1	0	1	2	4	4	2		2	2	2	2	3		2	2	2	2	1	2	1	2	1	1	2	1
1	38	2	2	1	2	0	2	2	2	2	2		28	2	0	0	2	4,6	4	1	2016-09-20 18:20	2	2	2	2	3		2	2	2	2	8.9	2	2	2	2	1	2	1
1	72	2	2	2	2	0	2	1	2	2	2		57	2	0	0	2	4	4	1	2016-09-27 01:57	2	2	2	2	3	0	2	2	2	2	23.6	2	2	2	2	1	2	1
2	46	1	2	1	2	0	2	2	2	2	2		26	2	1	0	2	4	4	1	2016-09-27 09:10	2	2	2	2	3		2	2	2	2	13.3	2	2	2	2	1	2	1
1	53	1	1	1	2	0	1	2	1	2	1		38	1	0	0	1	1	1	1	2016-09-29 17:10	2	2	2	2	3		2	1	1	2	20.6	1	2	1		1	2	1
2	30	1	2	1	2	0	2	2	2	2	2		40	2	0	0	1	1	1	1	2016-10-03 14:15	2	2	2	2	3		2	2	2	2	21.6	2	2	2	2	1	2	1
1	24	2	1	2	2	0	2	2	2	2	2		52	2	0	0	2	4	4	1	2016-10-05 13:15	2	2	2	2	3		2	1	1	2	27.8	2	2	2	2	1	2	1
2	65	2	1	2	2	0	2	1	1	2	2		22	1	1	0	1	1	1	1	2016-10-07 19:00	2	2	2	1	2		2	2	2	2	11.5	2	2	1		1	2	1
2	26	2	1	1	1	0	2	2	2	2	2		0	1	0	1	1	1,6	1	1	2016-11-14 18:20	2	2	1	2	1	1	2	2	2	2	4.2	2	2	1		0	1	0
2	68	2	2	1	2	0	2	1	2	2	2		24	1	1	0	1	1	1	1	2016-12-18 20:17	2	2	1	2	1		2	2	2	2	23.4	2	2	1		1	2	1
1	75	2	1	1	2	0	2	1	1	2	2		4	1	0	0	1	1	1	1	2016-12-21 19:45	2	2	1	2	1		2	1	1	1	23.5	2	1	2	3	1	2	1
1	66	2	1	1	1	0	2	2	2	2	2		75	1	0	1	2	4	4	1	2016-12-22 19:30	2	2	2	1	2		2	1	1	2	22.9	1	2	1		0	1	0
1	71	1	2	1	2	0	2	1	2	2	2		39	2	0	0	2	4	4	1	2017-01-11 14:35	2	2	2	2	3		2	2	2	2	20.8	2	2	2	3	1	2	1
1	47	1	2	1	2	0	2	2	2	2	2		40	2	0	0	2	4	4	1	2017-01-25 18:30	2	2	2	2	3		2	2	2	2	4.9	2	2	2	2	1	2	1
1	35	2	1	2	1	0	2	2	2	2	2		14	1	0	0	1	1	4	1	2017-01-25 18:30	2	2	2	1	2	0	2	1	1	2	13.7	2	2	1		0	1	0
2	45	1	2	1	2	0	2	2	2	2	2		30	2	0	0	2	4	4	1	2017-02-27 11:30	2	2	2	2	3		2	2	2	2	1.7	2	2	1		0	1	0
1	46	1	1	2	2	0	2	2	2	2	2		11	2	1	0	1	1	1	1	2017-03-05 17:30	2	2	2	2	3	0	2	2	2	2	13.4	2	2	1		1	1	1
1	46	1	2	2	2		2	2	2	2	2		53	1	0	0	2	4	4	2		2	2	2	2	3	0	2	1	2	1	0	2	1	2	4	1	2	1
2	49	1	2	1	2	0	2	2	2	2	2		6	2	0	0	1	1	1	1	2017-03-26 14:30	2	2	2	2	3		2	1	1	2	3.8	99	2	1		1	1	1
2	50	2	2	2	1	0	2	2	2	2	2		15	1	0	0	1	1	1	1	2017-04-20 20:00	2	2	1	2	1	0	2	1	1	2	4.7	2	2	1		0	1	0
1	21	2	2	1	2	0	2	2	2	2	2		35	2	0	0	2	4	4	1	2017-04-27 15:50	2	2	2	2	3	0	2	1	1	2	5.4	2	2	2	2	1	2	1
1	73	1	1	1	2	0	2	1	1	2	2		30	2	0	0	2	4	4	1	2017-04-30 17:50	2	2	2	2	3		2	1	1	2	2.5	99	2	2	3	1	2	1
1	70	2	1	1	2	0	2	1	1	2	1		10	2	0	0	1	1	1	1	2017-05-06 19:30	2	2	2	2	3		2	1	1	2	15	1	2	1		0	1	0
2	34	1	1	1	1	0	2	2	2	2	2		13	1	0	0	1	1	1	1	2017-05-11 02:25	2	2	2	1	2	0	2	2	2	2	13.9	2	2	1		0	1	0
1	45	2	2	1	2	0	2	2	2	2	2		31	2	0	0	2	4	4	1	2017-05-30 20:30	2	2	2	2	3	0	99	1	1	2	12.7	2	2	2	2	1	2	1
2	82	1	1	2	2	1	2	1	1	2	2		51	2	1	0	1	1	1	1	2017-06-01 21:30	2	2	2	2	3		2	2	2	2	10.5	2	2	2	3	1	2	1
1	46	1	2	1	2	0	2	2	2	2	2		25	2	1	0	1	1	1	1	2017-06-12 20:45	2	2	2	2	3		2	2	2	2	8.2	2	2	1		0	1	0
1	50	1	1	2	2	0	2	1	1	2	1		45	2	0	0	1	1	1	1	2017-06-14 20:40	2	2	2	2	3		2	2	2	2	21.8	1	2	2	3	1	2	1
1	64	1	2	1	2	0	2	2	2	2	2		57	1	1	0	1	1	1	1	2017-06-22 01:00	1	2	1	2	1		2	2	2	2	1.2	1	2	2	1	1	2	1
2	34	1	2	2	2	0	2	2	2	2	2		46	2	0	0	2	4	4	1	2017-06-27 02:38	2	2	2	2	3	0	2	2	2	2	14.5	2	2	2	2	1	2	1
1	21	2	2	2	2	0	2	2	2	2	2		49	2	0	1	2	4	4	1	2017-07-01 21:45	2	2	2	2	3		2	1	1	2	25.7	2	2	2	3	1	2	1
1	59	1	1	2	2	0	2	2	2	2	2		42	1	0	0	1	1	1	1	2017-07-04 03:00	2	2	2	1	2		2	2	2	2	30.3	2	2	1		1	1	1
1	79	2	1	2	1	0	2	1	1	2	2		31	1	0	0	2	4	4	1	2017-07-12 00:05	2	2	2	2	3	0	2	2	2	2	11.1	2	2	1		0	1	0
1	66	1	1	2	2	0	1	1	1	2	2		34	1	0	0	2	4	4	1	2017-07-16 11:00	1	1	2	1	2	1	2	2	2	2	12.8	2	2	1		1	1	1
2	82	2	1	1	2	0	2	2	1	2	2		10	2	0	0	2	4	4	1	2017-07-21 23:00	2	2	2	2	3		2	2	2	2	15.3	2	2	2	1	1	2	1
2	66	2	1	1	1		2	1	2	2	2		30	1	1	1	2	4	4	1	2017-07-25 10:00	2	1	2	1	2		2	2	2	2	21.8	2	2	1		1	1	
1	59	1	2	2	1	0	2	1	1	2	2		7	1	0	0	2	4	4	1	2017-08-07 10:10	2	2	1	2	1	0	2	2	2	2	5.7	2	2	1		0	1	0
1	81	2	2	2	2		2	2	2	2	2		35	2	1	0	2	4	4	2		2	2	2	2	3		2	2	2	2	4.6	2	2	2	4	1	2	1
2	38	1	2	2	2	0	2	2	2	2	2		19	2	1	0	1	2	2	1	2017-09-08 14:45	2	2	2	2	3	1	2	2	2	2	13.5	2	2	2	2	1	2	1
2	59	1	1	1	2		2	1	2	2	2		42	1	0	0	1	1	1	1	2017-09-13 05:00	2	2	1	2	1		2	2	2	2	31.3	2	2	2	1	1	2	1
1	72	1	1	2	2		1	2	1	2	1		57	1	0	0	1	1	1	1	2017-09-28 11:40	1	2	2	1	2		2	2	2	2	7	1	1	2	1	1	2	1
1	44	1	1	2	1	1	2	2	2	2	2		77	1	1	0	1	1,6	1	1	2017-10-02 10:15	1	1	2	1	2		2	1	2	2	32	1	2	2	1	1	2	1
2	65	2	1	1	1	0	2	2	1	2	2		39	1	1	0	1	1	1	1	2017-10-06 17:00	2	2	2	1	2		2	2	2	2	0.4	2	1	2	1	1	2	1
1	73	1	1	2	2		2	2	2	2	2		57	2	1	1	2	4	4	2		2	2	2	2	3		2	2	2	2	21.1	2	2	2	1	1	2	1
2	62	2	1	1	2		1	2	1	2	2		13	1	0	0	1	1	1	1	2017-11-01 00:30	2	1	2	1	2		2	1	2	2	6.8	2	2	1		1	1	1
1	61	1	1	1	1		1	2	2	2	2		32	1	1	0	1	1	1	1	2017-11-14 00:30	2	2	2	1	2	1	2	2	2	2	6.8	2	2	1		0	1	0
1	80	2	1	1	1		2	1	2	2	2		10	1	0	0	2	4	4	1	2017-12-05 16:24	1	2	2	1	2	1	2	1	1	2	12.3	2	2	2	1	1	2	1
1	58	1	1	1	2	0	2	2	2	2	2		33	2	0	0	2	4	4	1	2017-12-08 02:10	2	2	2	2	3		2	1	1	2	33.5	2	2	1		1	2	1
1	67	2	1	1	1	0	2	2	1	2	2		13	1	0	0	1	1	1	1	2017-12-07 17:30	2	2	2	1	2	1	2	2	2	2	10.3	2	2	1		0	1	0
1	47	2	1	1	2	0	2	1	2	2	2		49	1	0	0	1	1	1	1	2017-12-18 02:00	2	2	1	2	1	1	2	1	1	2	10.6	2	2	1		0	1	0
1	82	2	1	1	2	0	2	2	2	2	2		30	2	0	0	2	4	4	1	2017-12-19 20:20	2	2	2	2	3		2	1	1	2	25.2	2	2	2	1	1	2	1
1	32	1	2	1	2	1	2	2	2	2	2		30	2	0	0	2	4	4	1	2017-12-21 21:50	2	2	2	2	3	0	2	2	2	2	20.4	2	2	2	2	1	2	1
1	67	1	1	2	1	0	1	1	1	1	2		40	1	1	0	1	1	1	1	2017-12-31 14:15	1	2	2	1	2		2	2	2	2	26.7	1	2	2	1	1	2	1
1	54	2	2	1	2	0	2	2	2	2	2		16	2	0	0	2	4	4	1	2018-01-01 09:30	1	2	2	2	3		2	2	2	2	6.9	1	2	2	1	1	2	1
2	75	2	2	1	2	0	2	1	2	2	2		10	2	0	0	2	4	4	1	2018-01-12 01:20	2	2	2	2	3		2	1	1	2	27.9	2	2	1		1	2	1
2	53	2	1	1	2	1	2	2	1	2	2		18	2	0	0	2	4	4	1	2018-01-26 08:34	2	2	2	2	3		2	1	1	2	12.1	2	2	1		1	1	1
1	85	2	1	1	2	1	2	2	1	2	1		8	1	0	0	2	4	4	1	2018-01-28 20:45	2	2	2	1	2		2	1	2	1	9.3	1	1	2	1	1	2	1
1	77	2	1	2	2		2	1	2	2	2		29	1	1	0	2	4	4	2		2	2	2	2	3		2	1	1	2	17.7	2	1	2	1	1	2	1
1	79	1	1	1	2	0	1	1	1	2	2	1	11	1	0	0	2	4	4	1	2018-02-11 07:55	2	2	2	2	3		2	1	1	2	14.9	2	2	2	1	1	2	1
1	68	1	2	2	2	0	2	2	2	2	2		33	2	0	0	2	4	4	1	2018-02-17 18:30	2	2	2	2	3		2	2	2	2	2.9	2	2	1		1	2	1
1	43	2	1	2	1	0	2	1	1	2	2		112	1	0	0	2	4	4	1	2018-03-01 17:20	2	2	1	2	1		2	1	1	2	10.3	2	1	2	1	1	2	1
1	67	2	1	1	1	0	2	2	2	2	2		3	1	0	0	2	4	4	1	2018-03-06 19:48	2	2	1	2	1	0	2	1	2	2	7.5	2	2	1		0	1	0
1	19	2	2	1	1	0	2	2	2	2	2		50	1	0	0	1	1	1	1	2018-03-09 20:30	1	2	2	2	3	0	2	1	1	2	18.2	2	2	1		1	1	0
1	46	1	2	1	2	0	2	2	2	2	2		36	2	1	0	2	4	4	1	2018-03-11 06:24	2	2	2	2	3		2	2	2	2	13.9	2	2	1		1	2	1
1	82	1	1	2	2		2	2	1	2	2		36	1	0	0	2	4	4	2		2	2	2	1	2		2	2	2	2	4.7	2	1	2	1	1	2	1
1	84	2	2	1	2	0	2	1	2	2	2		44	1	1	0	2	4	4	2		2	2	1	2	1		2	1	1	2	0.1	2	1	2	1	1	2	1
1	69	1	2	2	2	0	2	2	1	2	2		60	2	1	0	2	4	4	1	2018-04-12 19:30	2	2	2	2	3		2	2	2	2	0.1	2	1	2	3	1	2	1
1	55	1	1	2	2	1	2	2	2	2	2		57	1	0	0	1	1	1	1	2018-02-27 21:10	2	2	2	2	3		2	1	1	2	24.3	2	1	2	2	1	2	1
1	65	2	1	1	1		2	1	2	2	2		38	1	0	0	1	1	1	2		2	2	1	2	1	1	2	2	2	2	0.8	2	1	2	1	1	2	1
1	46	2	1	1	1	0	2	2	2	2	2		13	1	0	0	2	4	4	1	2018-06-08 08:59	2	2	2	1	2	0	2	2	2	2	5.9	2	2	1		0	1	0
1	49	2	1	2	1	0	1	1	1	2	2		6	1	0	0	1	1	1	1	2018-07-11 09:45	1	2	2	1	2	1	2	1	1	2	15.7	1	2	1		0	1	0
2	70	1	1	1	2	0	2	1	1	1	2		28	2	0	0	1	1	1	1	2018-07-15 19:52	2	2	2	2	3		2	1	1	2	3.7	2	2	1		1	1	1
1	82	2	1	1	2	0	2	1	2	2	2		6	1	0	0	1	1	1	1	2018-07-30 13:15	2	2	1	2	1	0	2	1	2	2	10.4	2	1	1		0	1	0
1	76	2	1	2	2	0	2	1	2	2	2		35	1	0	0	1	1	1	2		2	2	2	2	3		2	1	2	1	13.2	2	1	2	1	1	2	1
1	60	2	2	1	1	0	1	1	2	2	2		11	1	0	0	1	1	1	1	2018-08-18 09:00	2	2	1	2	1	0	2	1	1	2	6	2	2	1		0	1	0
1	60	1	1	1	1	0	2	2	2	2	2		21	1	0	0	1	1	1	1	2018-08-28 09:15	2	2	1	2	1	0	2	2	2	2	9.7	2	2	1		0	1	0
2	34	2	1	1	1	0	1	2	2	2	2		15	1	0	0	1	1	1	1	2018-08-31 22:13	2	2	1	2	1	0	2	2	2	2	2	2	1	1		0	1	0
1	61	1	2	2	2	0	1	2	2	2	2		39	1	1	0	1	1	1	1	2018-09-01 21:13	2	2	1	2	1	0	2	1	1	2	19.9	2	2	1		1	1	1
1	57	2	1	2	2	0	2	2	2	2	2		41	2	0	0	1	1	1	1	2018-09-21 20:45	2	2	2	2	3		2	1	2	1	34.1	2	2	2	2	1	2	1
1	69	2	1	1	2	0	2	1	1	2	1		23	2	0	0	1	1	1	1	2018-10-15 03:22	2	2	2	2	3		2	2	2	2	8.9	1	2	1		1	2	1
1	57	2	1	1	1	0	2	2	2	2	2		25	1	0	0	1	1	1	1	2018-10-19 19:45	2	2	2	1	2	1	1	2	2	2	13.8	2	2	1		1	1	1
1	33	1	2	1	2	0	2	2	2	2	2		5	2	1	0	2	4	4	1	2018-10-27 14:45	2	2	2	2	3		2	2	2	2	9.4	2	2	1		0	1	0
1	45	2	1	1	2	1	2	1	2	2	2		33	1	0	0	2	4	4	1	2018-11-18 03:55	2	2	2	2	3		2	1	1	2	11.3	2	2	1		1	1	1
1	26	2	1	1	1	0	2	2	2	2	2		15	1	0	0	2	4	4	1	2018-12-01 05:19	2	2	1	2	1	0	2	2	2	2	4.6	2	2	1		0	1	0
1	67	2	2	1	1	0	2	2	1	2	2		31	1	1	0	1	1	1	1	2018-12-11 18:45	1	2	2	1	2	1	2	2	2	2	11.7	2	2	1		1	2	1
1	48	2	1	1	1	0	1	2	2	2	2	1	16	1	0	0	1	1	1	1	2018-12-16 01:22	2	2	2	1	2	0	2	2	2	2	3.1	2	2	1		0	1	0
1	56	2	1	1	2	0	1	1	1	2	1	1	18	1	0	0	1	1	1	1	2018-12-23 20:37	2	2	2	2	3		2	1	1	2	20.5	1	2	2	1	1	2	1
2	75	2	1	1	2	0	2	1	2	2	2		49	1	0	0	1	1,5	1	2		2	2	2	2	3		2	2	2	2	0.1	2	1	2	1	1	2	1
1	56	1	1	1	2	0	2	2	2	2	2		20	1	0	0	1	1	1	1	2016-03-24 21:10	2	2	2	2	3	0	2	1	2	2	14.1	2	2	1		1	1	1
1	60	2	1	1	2	0	2	1	2	2	2		14	2	0	0	1	1,5,6	1	1	2016-03-28 01:40	2	2	2	2	3		2	1	1	2	0.2	2	2	1		1	1	1
1	54	1	2	1	2	0	2	1	2	2	2		62	1	1	0	1	1,6		1	2016-03-27 16:50	2	2	1	2	1		2	2	2	2	15.6	2	2	2	1	1	2	1
2	53	2	2	1	2	0	2	2	2	2	2		27	2	0	0	1	1,5,6	1	1	2016-04-24 18:45	2	2	2	2	3		2	2	2	2	5.6	2	2	1		0	1	0
1	76	2	1	2	2	0	2	1	2	2	2		82	2	1	0	1	1	1	2		2	2	2	2	3		2	2	2	2	29.3	2	2	2	3	1	2	1
1	76	1	1	1	2	0	2	2	2	2	2		40	2	1	0	1	1,6	1	1	2016-05-16 17:45	2	2	2	2	3		2	2	2	2	0.2	2	2	2	3	1	2	1
2	73	2	1	1	1	0	1	1	2	2	2	1	21	1	0	0	1	1,5,6	1	1	2016-06-12 03:55	2	2	1	2	1	1	2	2	2	2	5.2	2	2	1		0	1	0
2	77	1	2	2	2	0	2	1	2	2	2		44	2	0	0	1	1,5,6	1	1	2016-06-17 18:25	2	2	2	2	3		2	2	2	2	1.5	2	1	2	3	1	2	1
2	56	1	2	2	2	0	2	2	2	2	1		28	2	0	0	1	1,5,6	1	1	2016-06-20 00:20	2	2	2	2	3		2	2	2	2	12.1	1	2	1		1	2	1
1	55	2	2	2	2	0	2	1	2	1	2		31	2	1	0	1	1,5,6	1	1	2016-07-16 18:01	2	2	2	2	3		2	2	2	2	0.2	2	1	2	3	1	2	1
1	54	2	1	1	2	0	2	2	2	2	2		4	2	0	0	1	1,5	1	1	2016-07-21 21:25	2	2	2	2	3		2	1	2	2	6.6	2	1	2	4	1	2	1
1	75	1	2	1	2	0	2	1	1	2	2		36	2	1	0	1	1,5,6	1	1	2016-07-27 06:00	2	2	2	2	3		2	2	2	2	0.1	2	2	2	3	1	2	1
1	25	2	1	1	2	0	2	2	2	2	2		13	2	0	0	1	1,5,6	1	2		2	2	2	2	3		2	1	2	1	0.7	2	1	2	3	1	2	1
1	47	1	1	1	2	0	2	2	1	2	2		41	2	1	0	1	1,2,5,6	1	1	2016-09-05 16:00	2	2	2	2	3		2	1	1	2	0.3	2	1	2	3	1	2	1
1	62	1	2	2	1	0	2	2	2	2	2		37	1	0	0	1	1	1	1	2016-09-07 14:30	2	2	2	2	3	0	2	1	2	1	37	2	2	2	2	1	2	1
1	39	1	1	1	1	0	2	2	2	2	2		17	1	0	0	1	1,5,6	1	1	2016-09-22 17:00	2	2	1	2	1	0	2	1	2	2	5.4	2	2	1		0	1	0
2	68	1	1	1	2	0	2	2	1	2	1		69	2	0	0	1	1,5,6	1	1	2016-09-26 20:30	2	2	2	2	3		2	1	1	2	0.9	1	2	2	3	1	2	1
1	45	1	1	1	1	0	2	2	2	2	2		20	1	1	0	1	1,5,6	1	1	2016-09-30 21:45	2	2	1	2	1	0	2	1	1	2	38.2	2	1	1		0	1	0
1	61	1	1	2	2	0	2	2	2	2	2		20	2	0	0	1	1,5,6	1	1	2016-10-13 18:35	2	2	2	2	3	0	2	1	1	2	0.4	2	2	2	3	1	2	1
1	72	2	1	1	2	0	2	1	2	2	2		28	2	0	0	1	1,5,6	1	1	2016-11-01 13:45	2	2	2	2	3	0	2	1	1	2	4.8	2	2	1		1	2	1

2	84	2	1	1	2	0	2	1	1	1	2		22	2	0	0	1	1,5		1	1	2016-11-22 17:00	2	2	2	2	3		2	1	1	2		19	2	2	1		1	1	1
1	51	2	1	2	1	0	2	2	2	2	2		36	1	0	0	1	1,5,6		1	1	2016-11-28 11:45	2	2	1	2	1	0	2	2	2	2		13.2	2	1	1		0	1	0
1	60	2	2	2	2	0	2	2	1	2	2		9	2	0	0	1	1,5,6		1	1	2016-11-30 22:00	2	2	2	2	3	0	2	1	2	1		13.6	2	2	1		1	1	0
1	50	2	1	2	2	0	2	2	2	2	2		28	1	1	0	1		1	1	1	2016-12-03 16:50	2	2	2	2	3		2	1	2	2		6.5	2	2	1		1	1	1
1	64	1	1	1	2	0	2	1	2	2	2		77	1	1	0	1		1		2		2	2	2	2	3		2	2	2	2		0	2	2	2	2	1	2	1
2	82	1	1	1	2	1	2	1	2	2	2		35	1	0	0	1	1,5			2		2	2	2	2	3		2	2	2	2		0.2	2	1	2	1	1	2	1
1	55	1	1	1	1	0	2	2	2	2	2		28	2	0	0	1	1,5		1	1	2016-12-23 21:30	2	2	2	2	3		2	1	2	1		0.1	2	2	2	3	1	2	1
1	73	2	1	1	1	0	2	2	2	2	2		13	1	0	0	1	1,5		1	1	2016-12-27 19:00	2	2	2	1	2		2	1	2	2		0.3	2	1	2	1	1	2	1
1	79	2	1	1	2	0	2	2	2	1	2		30	2	1	0	1	1,5,6		1	1	2016-12-28 20:25	2	2	2	2	3		2	1	1	2		7.2	2	2	1		1	1	1
2	67	1	1	1	2	0	2	1	2	2	2		40	1	0	0	1	1,5,6		1	1	2017-01-05 14:40	2	2	2	2	3		2	1	1	2		20.6	2	2	1		1	2	1
1	70	2	1	1	1	0	2	1	1	2	2		12	1	1	0	1	1,5,6		1	1	2017-01-13 14:25	2	2	2	1	2	1	2	1	2	2		25.4	2	2	1		0	1	0
1	81	2	1	1	2	0	2	2	1	2	1		18	2	0	0	1	1,5,6		1	2		2	2	2	2	3		2	2	2	2		0.1	2	2	2	3	1	2	1
1	54	1	2	2	2	0	2	2	2	2	2		32	2	1	0	1	1,6		1	1	2017-02-22 01:30	2	2	2	2	3		2	1	1	1		0.1	2	2	2	3	1	2	1
1	83	2	1	2	1	0	1	2	2	2	2		11	1	0	0	1	1,5,6		1	1	2017-02-24 11:00	2	2	2	2	3		2	1	1	2		0	1	2	2	3	1	2	1
2	77	2	1	1	2	0	2	1	1	2	2		48	1	0	0	1	1,5,6		1	1	2017-02-28 15:30	2	2	2	2	3		2	1	1	2		38.2	2	2	1		1	2	1
1	49	2	1	1	2	0	2	2	2	2	2		65	2	0	0	1	1,5,6			2		2	2	2	2	3		2	2	2	2		1.1	2	2	2	3	1	2	1
1	53	2	1	1	2	0	1	1	1	2	2		66	1	0	0	1	1,5,6			2		2	2	2	2	3	0	2	1	2	2		0.3	2	2	1		1	2	1
1	42	1	1	2	1	0	2	1	2	2	2		149	1	0	0	1	1,5,6,10		1	1	2017-03-18 09:05	2	2	2	2	3	0	2	2	2	2		0.4	1	2	2	3	1	2	1
1	60	2	2	1	2	0	2	1	1	2	2		43	1	0	0	1	1,5,6		1	1	2017-03-29 13:25	2	2	2	2	3	0	2	1	1	2		11.8	1	2	2	3	1	2	1
2	71	1	1	1	2	0	2	1	2	2	2		47	2	0	0	1	1,5,6		1	2		2	2	2	2	3		2	1	2	1		29.4	2	1	2	3	1	2	1
1	60	2	2	1	2	0	2	1	2	2	2		20	2	0	0	1	1,5,6		1	1	2017-04-18 16:40	2	2	2	2	3		2	1	2	1		12	2	2	2		1	1	1
2	76	2	2	2	2	0	2	2	2	2	2		18	2	1	0	1	1,5,6		1	1	2017-04-28 16:45	2	2	2	2	3		2	1	1	2		19.1	1	2	1		1	1	1
1	53	1	2	1	2	0	2	2	2	2	2		60	1	0	0	1	1,5,6			2		2	2	2	2	3		2	2	2	2		0	2	1	2	1	1	2	1
1	88	1	1	1	1	0	1	2	2	2	2	1	12	1	0	0	1	1,5,6			2		2	2	2	2	3		2	2	2	2		0.1	2	1	2	1	1	2	1
2	86	2	1	1	2	0	2	1	2	2	2		9	2	0	0	1	1,5,6		1	1	2017-05-11 15:10	2	2	2	2	3		2	1	1	2		12.3	2	2	1		1	1	1
2	87	1	1	1	2	0	2	2	2	2	2		33	2	0	0	1	1,5,6		1	2		2	2	2	2	3		2	1	1	2		15.9	2	2	1		0	1	0
1	71	2	1	1	2	0	1	1	2	2	2		8	1	0	0	1	1,5,6		1	1	2017-05-28 10:30	2	2	1	2	1	1	2	1	1	2		14.5	1	1	1		0	1	0
1	41	1	2	1	2	0	2	2	2	2	2		38	2	0	0	1	1,5,6		1	1	2017-06-12 22:00	2	2	2	2	3		2	1	1	2		19.1	2	2	2	2	1	2	1
1	64	2	1	2	2	0	2	1	1	2	2		42	2	1	0	1	1,5,6			2		2	2	2	2	3		2	1	2	1		27.4	2	1	2	3	1	2	1
2	52	1	2	1	2	0	2	2	2	2	2		61	2	1	0	1	1,5,6		1	1	2017-07-04 00:45	2	2	2	2	3		2	1	1	2		22.6	2	2	2	2	1	2	1
1	68	1	1	2	1	0	1	1	2	2	2		13	1	1	0	1	1,5,6		1	1	2017-07-06 21:25	2	2	2	1	2	0	2	2	2	2		6.3	2	2	1		0	1	0
1	59	2	1	2	2	0	2	2	2	2	2		64	1	1	0	1	1,5,6		1	1	2017-08-07 18:50	2	2	1	2	1	1	2	2	2	2		0.3	1	2	2	3	1	2	1
2	49	1	1	2	1	0	2	1	2	2	2		12	1	0	0	1	1,5,6		1	1	2017-08-11 12:00	2	2	1	2	1	0	2	2	2	2		2.1	2	2	1		0	1	0
1	23	1	2	2	2	0	2	2	2	2	2		80	2	0	0	1	1,5,6		1	1	2017-08-14 16:20	2	2	2	2	3		2	2	2	2		30.5	2	2	2	2	1	2	1
1	75	2	2	2	2	0	2	1	2	2	1		73	2	0	0	1	1,5		1	1	2017-08-18 19:50	2	2	2	2	3		2	1	1	2		17.2	1	2	2	3	1	2	1
2	49	2	1	2	2	0	2	2	2	2	2		75	2	1	0	1		1	1	1	2017-08-21 16:50	2	2	2	2	3		2	1	2	1		23.5	1	2	2	3	1	2	1
1	87	1	2	2	2	0	1	2	1	2	2		42	2	0	0	1		1	1	1	2017-08-28 15:05	2	2	2	2	3		2	2	2	2		0.5	1	2	2	3	1	2	1
1	67	1	1	1	1	0	2	2	2	2	2		7	1	0	0	1	1,5,6		1	1	2017-08-29 21:00	2	2	2	2	3	0	2	1	1	2		11.4	2	2	1		0	1	0
2	43	1	2	1	2	0	2	2	2	2	2		27	2	0	0	1	1,5		1	1	2017-09-26 18:20	2	2	2	2	3		2	2	2	2		24.5	2	2	1		1	1	1
2	79	2	1	2	2	0	2	2	2	2	2		24	2	1	0	1	1,5		1	1	2017-10-20 20:30	2	2	2	2	3		2	1	1	2		16.5	1	2	2	3	1	2	1
2	45	1	2	1	2	0	2	2	2	2	2		40	2	0	1	1	1,5		1	1	2017-10-22 05:38	2	2	2	2	3		1	2	2	2		0.6	2	2	2	3	1	2	1
2	49	1	1	1	2	0	2	2	2	2	2		24	1	0	0	1	1,5		1	1	2017-10-27 19:52	2	2	2	2	3	0	2	1	2	2		4.7	2	1	1		1	1	1
1	66	1	2	1	2	0	2	2	2	2	2		22	2	0	0	1	1,5		1	1	2017-10-31 00:10	2	2	2	2	3		2	2	2	2		22.6	2	2	2	3	1	2	1
1	42	1	2	1	1	0	2	2	2	2	2		22	1	0	0	1	1,5,6		1	1	2017-11-03 14:45	2	2	1	2	1	0	2	1	1	2		8	2	2	1		0	1	0
1	58	2	1	1	1	0	2	2	2	2	2		50	1	1	0	1	1,5		1	1	2017-11-06 20:50	2	2	2	2	3	0	2	1	2	1		1.1	1	1	2	3	1	2	1
1	72	2	1	1	2	0	1	1	2	2	2		23	1	1	0	1	1,5		1	1	2017-11-04 16:40	2	2	1	2	1	0	2	1	1	2		15.8	2	1	1		0	2	1
1	32	1	2	1	2	0	2	2	2	2	2		40	2	0	0	1	1,5		1	1	2017-11-13 16:55	2	2	2	2	3		2	1	2	2		9	1	2	1		1	2	1
1	62	2	1	1	2	0	2	1	1	1	2		16	2	0	0	1		1	1	1	2017-11-25 13:30	2	2	2	2	3		2	1	1	2		34	2	2	1		1	2	1
1	33	2	1	1	1	0	2	2	2	2	2		20	1	0	0	1	1,5		5	1	2017-11-30 20:40	2	2	1	2	1	0	2	1	2	2		18.1	2	2	1		0	1	0
1	69	2	1	1	2	0	1	2	2	2	1		53	2	0	0	1	1,5		1	1	2017-12-01 18:00	2	2	2	2	3		2	1	1	2		10.5	1	2	1		1	2	1
2	86	2	1	1	2	0	2	1	2	2	1		34	2	0	0	1		1	1	1	2017-12-22 21:40	2	2	2	2	3		2	1	1	2		17	2	2	2	3	1	2	1
2	70	2	1	1	2	0	2	1	2	2	2		36	2	0	0	1	1,5			2		2	2	2	2	3		2	2	2	2		0	2	2	2	3	1	2	1
2	59	1	1	2	2	0	2	2	2	2	2		54	1	1	0	1	1,5		1	1	2018-01-02 18:50	2	2	2	2	3	0	2	1	2	2		0.1	1	2	2	3	1	2	1
1	74	2	2	2	2	0	2	2	1	2	2		40	2	0	0	1	1,5,10		1	1	2018-01-04 19:00	2	2	2	2	3	0	2	1	2	2		0.5	1	2	2	3	1	2	1
2	57	1	2	1	2	0	2	2	2	2	2		65	2	0	0	1	1,5		1	2		2	2	2	2	3		2	2	2	2		0.5	2	1	2	2	1	2	1
2	66	2	1	2	1	0	1	2	2	2	1		72	1	1	0	1	1,5,6		1	1	2018-01-20 21:30	2	2	2	2	3	0	2	1	2	2		1.8	1	2	2	3	1	2	1
2	76	2	2	2	2	0	2	2	2	2	2		18	2	1	0	1	1,5,6		1	2		2	2	2	2	3		2	1	1	1		15.8	2	2	2	3	1	2	1
1	69	1	1	2	2	0	2	2	2	2	2		53	2	0	0	1		1	1	1	2018-02-01 15:05	2	2	2	2	3		2	1	2	2		0.1	1	2	2	3	1	2	1
2	58	1	1	1	1	0	2	2	2	2	2		27	1	0	0	1	1,5		1	1	2018-02-06 14:45	2	2	1	2	1	1	2	2	2	2		16.8	2	2	1		0	1	0
1	66	2	1	1	2	0	2	2	1	1	2		23	2	1	0	1	1,5,6		1	1	2018-02-13 18:00	2	2	2	2	3		2	1	1	2		30	2	1	2	3	1	2	1
1	57	1	1	1	2	0	2	2	2	2	2		19	2	1	0	1	1,5,6		1	1	2018-02-21 17:00	2	2	2	2	3		2	1	1	2		25.7	2	2	1		1	2	1
1	51	1	1	1	1	0	2	2	2	2	2		32	1	0	0	1	1,5,6		1	1	2018-03-10 17:00	2	2	2	1	2	1	2	1	1	2		9.9	2	1	1		1	2	1
1	59	2	2	1	1	0	2	2	2	2	2		54	1	0	0	1	1,5		1	1	2018-03-22 19:40	2	2	2	2	3	1	2	1	1	2		11.3	2	2	2	3	1	2	1
1	72	2	1	1	1	0	2	1	2	2	2		11	1	1	0	1	1,5		1	1	2018-03-17 13:40	2	2	2	2	3	1	2	1	1	2		13.6	2	2	1		1	2	1
1	78	2	1	1	2	0	2	2	2	1	2		22	2	1	0	1	1,5		1	1	2018-03-31 01:30	2	2	2	2	3		2	1	1	1		14.8	2	2	1		1	2	1
1	23	1	1	2	1	0	2	2	2	2	2		18	1	0	0	1	1,5,6		1	2		2	2	1	2	1	0	2	2	2	2		7.8	2	2	1		0	1	0
2	32	1	2	1	2	0	2	2	2	2	2		33	2	1	0	1		1	1	1	2018-04-09 17:10	2	2	2	2	3		2	1	2	2		18.4	1	2	1	2	1	2	1
1	51	2	1	2	2	1	1	1	2	2	1		6	2	0	0	1	1,5		1	1	2018-04-18 15:00	2	2	2	2	3		2	1	1	2		11.6	1	2	1		0	1	0
2	80	1	2	2	2	0	2	2	2	2	2		20	1	0	0	1	1,5		1	1	2018-04-21 14:25	2	2	2	2	3	0	2	1	1	2		25.2	2	2	2	3	1	2	1
1	66	2	2	2	2	0	2	1	2	2	2		32	1	0	0	1	1,5		1	1	2018-04-25 20:50	2	2	2	2	3		2	1	2	2		8.2	2	1	1		1	2	1
1	65	1	1	2	2	0	2	2	2	2	2		12	1	0	0	1	1,5,6		1	1	2018-05-03 18:30	2	2	1	2	1	0	2	2	2	2		11.8	2	2	1		0	1	0
1	52	2	1	2	1	0	2	2	2	2	2		28	1	0	0	1	1,5,6,7		1	1	2018-05-08 18:00	2	2	2	2	3		2	1	1	2		8.6	2	2	1		0	1	0
2	79	2	1	1	2	0	2	1	2	2	1		8	2	0	0	1		1	1	1	2018-07-02 22:45	2	2	2	2	3		2	1	1	2		32.4	1	2	2	3	1	2	1
2	82	2	2	2	2	0	2	1	2	2	2		2	1	1	0	1	1,5		1	1	2018-07-06 22:00	2	2	2	2	3		2	2	2	2		0.6	2	2	2	3	1	2	1
2	82	2	2	1	2	0	2	1	1	2	2		9	1	0	0	1	1,5		1	1	2018-07-24 17:00	2	2	2	2	3		2	1	2	1		7.5	2	1	2	3	1	2	1
1	58	2	1	1	1	0	2	1	1	2	2		23	1	0	0	1		1	1	1	2018-07-22 17:00	2	2	1	2	1	1	2	1	1	2		16.9	2	2	1		0	1	0
1	85	2	1	1	2	0	2	2	2	2	2		54	2	0	0	1		1	1	1	2018-07-30 17:10	2	2	2	2	3		2	1	1	2		0.8	2	2	1		1	2	1
2	76	2	1	1	1	0	2	2	2	2	2		10	1	0	0	1	1,5		1	1	2018-08-03 14:40	2	2	2	2	3	0	2	1	1	2		33.9	1	1	1		1	2	1
2	77	2	1	2	2	0	2	1	2	2	2		42	2	0	0	1	1,5,6		1	1	2018-09-28 12:16	2	2	2	2	3		2	1	2	1		0.1	2	1	2	3	1	2	1
1	46	2	2	2	2	0	1	1	2	2	2		26	1	0	0	1	1,5,6		1	1	2018-10-02 20:30	2	2	2	2	3		2	1	2	2		6.5	2	1	1		1	1	1
1	77	1	1	2	2	0	2	2	1	2	1		46	2	0	0	1	1,5		1	1	2018-11-02 18:50	2	2	2	2	3		2	1	1	2		7	2	2	2	3	1	2	1
1	45	2	2	1	2	0	2	1	2	1	2		36	1	0	0	1	1,5,6		1	1	2018-12-05 16:30	2	2	2	2	3		2	1	2	2		0.4	2	1	2	2	1	2	1
2	81	1	1	2	2	0	2	1	2	2	2		60	2	0	0	1		1	1	1	2018-12-11 22:40	2	2	2	2	3		2	1	2	1		17.8	2	2	2	3	1	2	1
1	49	1	1	1	2	0	2	1	1	2	2		34	1	0	0	1		1	1	1	2018-12-30 07:42	2	2	1	2	1	0	2	1	1	2		8.6	2	2	1		1	1	1
1	67	1	1	2	2	0	2	1	2	2	2		38	2	0	0	1		1	1	1	2015-12-05 19:00	2	2	2	2	3	0	2	2	2	2		2.2	2	2	1		1	1	1
1	59	1	1	1	2	0	2	2	2	2	1		55	2	1	0	1		1	1	1	2016-01-13 20:00	2	2	2	2	3	0	2	2	2	2			2	2	2	3	1	2	1
2	46	1	1	1	1	0	2	2	2	2	2		8	1	1	0	1		1	1	1	2016-02-08 19:00	2	2	2	1	2	0	2	2	2	2		11.9	2	2	1		0	1	0
2	38	2	2	2	2	0	2	2	2	2	2		31	1	0	0	1		1	1	1	2016-03-05 20:00	2	2	2	2	3	0	2	1	2	2		5.5	2	2	1		1	1	1
1	76	2	1	1	2		2	2	2	2	1		28	1	1	0	1		1		2		2	2	2	2	3		2	2	2	2		5.4	2	1	2	4	1	2	1
1	83	2	2	1	1	0	2	2	2	2	2		33	1	0	0	1		1	1	1	2016-08-04 22:00	2	2	2	2	3		2	2	2	2		5.3	2	2	1		1	2	1
2	70	1	1	1	2	0	2	2	1	2	2		36	1	0	1	1		2	2	1	2016-11-19 09:30	2	2	2	2	3		2	2	2	2		5	2	2	1		1	1	1
1	58	1	1	1	1	0	2	1	2	2	2		16	1	1	1	1	1,6		1	1	2016-12-11 14:00	2	2	2	2	3	1	2	2	2	2		13.4	2	2	1		0	1	0
1	45	1	1	1	1	0	2	2	2	2	2		34	1	1	0	1		1	1	1	2016-12-21 18:00	2	2	1	2	1	0	2	2	2	2		0	2	2	1		0	1	0
1	46	1	2	1	2	0	2	2	2	2	2		30	2	1	1	1	2,6		2	1	2016-12-17 22:00	2	2	2	2	3		2	2	2	2		18.6	2	2	2	3	1	2	1
2	34	1	1	2	2	0	2	2	2	2	2		20	1	1	0	1	2,6		2	1	2017-01-21 02:00	2	2	2	2	3	0	2	1	1	2		3.9	2	2	1		1	2	1
1	35	2	1	2	1	0	2	2	2	2	2		14	1	0	0	1		2	2	2		2	2	2	2	3		2	2	2	2		0.6	2	2	1		0	1	0
1	42	1	1	2	1	0	2	2	2	2	2		8	1		0	1		1	1	1	2017-02-06 14:00	2	2	1	2	1	0	2	2	2	2		1.1	2	2	1		0	1	0
2	54	1	1	1	2	1	2	2	1	2	2		30	2	0	0	1		1	1	1	2017-05-05 13:00	2	2	2	2	3		2	1	1	2		0	2	2	2	2	1	2	1
1	37	1	1	2	2	0	2	2	2	2	2		56	1	1	0	1		1	1	1	2017-08-27 13:00	2	2	2	2	3		2	1	1	2		19.6	2	1	1		1	1	1
1	35	1	1	2	1	0	2	2	2	2	2		26	1	0	0	1		1	1	1	2017-09-24 13:00	2	2	2	2	3	0	2	2	2	2		0.1	2	2	1		0	1	0
1	24	1	1	1	1	0	2	2	2	2	2		12	1	0	0	1		1	1	1	2017-09-28 19:00	2	2	2	2	3	0	2	2	2	2		0	2	2	1		0	1	0
1	81	2	1	2	2	0	2	2	1	2	1		18	2	0	0	1	1,6		1	1	2018-02-13 19:50	2	2	2	2	3	0	2	2	2	2		13.2	2	2	2	3	1	2	1
2	47	1	1	1	2	1	2	2	2	2	2		13	2	0	0	1		1	1	1	2018-02-20 10:00	2	2	2	2	3	1	2	1	1	2		0.2	2	2	2	2	1	2	1
1	74	2	1	1	1	0	2	2	2	2	1		15	1	0	1	1		1	1	1	2018-04-23 18:20	2	2	2	2	3		2	2	2	2		0	1	1	2	1	1	2	1
2	67	2	1	2	2	0	2	1	1	2	1		11	2	0	0	1		1	1	1	2018-05-14 16:00	2	2	2	2	3		2	1	1	2		0	2	2	1		1	1	1
2	54	2	1	2	2	0	2	2	2	2	2		9	1	0	1	1		1	1	1	2018-06-16 22:00	2	2	1	2	1	1	2	2	2	2		14.4	2	2	1		0	1	0
1	81	1	1	1	2	0	2	1	1	2	2		45	1	0	1	1		1	1	1	2018-07-16 19:00	2	2	2	2	3	1	2	2	2	2		0	2	2	2	1	1	2	1
2	74	1	1	1	2	0	2	1	1	2	2		39	1	0	1	1		1	1	1	2018-09-12 20:00	2	2	2	2	3		2	2	2	2		1.1	2	2	2	2	1	2	1
1	70	1	1	1	1	0	2	1	1	2	2		48	1		1	1		1	1	1	2018-09-19 18:15	2	2	2	2	3		2	2	2	2		0	2	2	1		1	1	1
1	61	2	1	1	2	0	2	2	2	2	2		48	2	0	1	1		1	1	1	2018-09-07 18:00	2	2	2	2	3		2	2	2	2		0	1	2	2	2	1	2	1
2	55	1	1	2	2	0	2	2	2	2	2		59	1	0	1	1		1	1	1	2018-09-09 17:00	2	2	2	2	3	0	2	1	2	2		1.2	2	2	1		1	1	1
1	45	2	1	1	2	0	2	2	2	2	2		24	1	0	0	1		1	1	1	2018-11-26 21:00	2	2	2	1	2	1	2	2	2	2		1.1	2	2	1		0	1	0
1	57	1	2	2	2	0	1	2	2	2	2		25	1	0	1	3		9	9	1	2015-10-28 11:20	1	2	1	2	1	0	2	2	2	2		12.8	2	2	1		0	1	0
2	69	1	1	1	2	0	2	2	1	2	2		38	1	0	1	1	1,6		1	1	2015-10-28 16:55	2	2	1	2	1		2	1	1	2		12.8	2	1	2	3	1	2	1
1	46	2	1	1	1	0	2	2	2	2	2		23	1	1	0	1	1,8		1	1	2015-11-14 10:00	2	2	1	2	1	0	2	1	1	2		14.2	2	2	1		0	1	0

2	42	1	1	1	1	0	2	2	2	2	1		28	1	0	0	1	1	1	1	2015-11-04 18:20	2	2	1	2	1	0	2	2	2	2		19.2	2	2	1		0	1	0
2	77	1	2	2	2	0	2	1	2	2	2		32	2	0	1	1	1	1	1	2015-11-26 16:40	2	2	2	2	3		2	1	1	2		28	2	2	1		1	1	1
1	51	2	1	1	1	0	2	1	2	2	2		15	1	0	0	1	1	1	1	2015-11-25 02:05	2	2	1	2	1	0	2	1	2	1			1	2	2	3	1	2	1
2	75	1	2	1	2	0	2	1	1	2	2		47	1	1	1	1	1	1	1	2015-11-23 16:30	2	2	2	2	3		2	2	2	2		0.5	2	1	2	1	1	2	1
1	26	2	2	1	2	0	2	2	2	2	2		8	2	0	0	1	1,8	1	1	2015-11-29 16:38	2	2	2	2	3		2	1	1	2		13.7	2	2	1		0	1	0
1	64	2	1	1	2	0	2	2	2	2	2		22	2	0	0	1	1	1	1	2015-12-04 07:48	2	2	2	2	3	0	2	1	1	2		25.6	2	2	2	2	1	2	1
1	80	2	2	1	2	0	2	2	2	2	2		32	1	1	1	1	1	1	1	2015-12-04 18:17	2	2	2	2	3		2	2	2	2		5.2	1	2	2	3	1	2	1
1	37	1	2	1	2	0	2	2	2	2	2		9	1	1	0	1	2	2	1	2015-12-07 02:15	2	2	1	2	1	0	2	1	1	2		13.4	2	2	1		1	2	1
1	68	2	1	1	2	0	1	2	2	2	2		25	1	0	1	1	1	1	1	2016-01-03 17:10	2	2	2	1	2	1	2	1	1	2		18.9	2	2	1		0	1	0
1	66	1	2	2	2	0	2	2	2	1	2		59	2	1	1	1	1	1	1	2016-01-21 20:50	2	2	1	2	1		2	1	1	2		18	2	2	2	2	1	2	1
1	44	1	1	1	1	0	2	2	2	2	2		23	1	1	1	1	1	1	1	2016-01-24 11:25	2	2	1	2	1	0	2	1	1	2		15.1	2	2	1		1	1	1
1	71	1	1	2	2	0	1	1	2	2	2		48	1	0	1	1	1	1	1	2016-02-04 08:58	2	2	1	2	1		2	2	2	2		23.4	2	1	2	1	1	2	1
1	70	1	1	1	1	0	2	1	2	2	2		20	1	0	1	1	1,8	1	1	2016-02-07 00:55	2	2	2	1	2		2	2	2	2		21.9	2	2	2	1	1	2	1
1	59	1	1	1	1	0	1	2	2	2	2		25	1	0	1	1	1,8	1	1	2016-02-21 17:30	2	2	2	1	2	1	2	1	1	2		22.5	2	1	1		0	1	0
1	57	2	1	1	2	0	1	2	2	2	2		26	1	0	1	1	1,8	1	1	2016-02-23 08:00	2	2	1	2	1	0	2	2	2	2		21.5	2	2	1		0	1	0
1	58	1	2	2	2	0	2	2	2	2	2		80	1	0	1	1	1,8	1	1	2016-02-28 20:48	2	2	1	2	1		2	1	1	2		30	1	2	2	2	1	2	1
1	59	1	1	2	2	0	2	2	2	2	2		39	1	1	1	1	1,8	1	1	2016-03-06 10:10	2	2	1	2	1		2	1	1	2		12.9	2	2	2	3	1	2	1
2	55	2	2	2	2	0	2	2	2	2	2		9	2	0	1	1	1,8	1	1	2016-03-19 12:10	2	2	2	2	3	0	2	1	1	2		26.9	2	2	1		0	2	1
1	40	2	1	1	1	0	2	1	2	2	2		31	1	1	1	3	9	9	1	2016-03-25 16:55	1	2	1	2	1	1	2	2	2	2		14.7	1	2	1		0	1	0
2	20	1	1	1	1	0	2	2	2	2	2		43	1	0	1	1	1,8	1	1	2016-03-28 10:25	2	2	1	2	1	0	2	2	2	2		96.5	2	2	1		0	1	0
1	67	2	2	1	2	0	2	1	2	2	2		32	2	1	1	1	1,8	1	1	2016-04-15 03:27	2	2	2	2	3		2	2	2	2		15.1	2	2	1		1	2	1
1	64	1	1	1	2	0	2	1	2	2	2		29	1	0	1	1	1,8	1	1	2016-05-09 13:50	2	2	1	2	1		2	1	1	2		16.7	2	2	1		0	1	0
1	75	2	2	2	2	0	2	1	1	2	2		13	2	0	1	1	1	1	1	2016-05-23 09:30	2	2	2	2	3	0	2	1	1	2		25.2	2	2	1		0	1	0
1	46	1	1	1	2	0	1	2	2	2	2		41	1	0	1	1	1,8	1	1	2016-05-29 09:18	2	2	1	2	1	0	2	1	1	2		23.2	2	2	1		0	1	0
1	35	2	2	2	2	0	2	2	2	2	2		41	2	1	1	1	1,2,8	1,8	1	2016-06-02 14:25	2	2	2	2	3		2	1	1	2		51.3	2	2	1		0	1	0
1	49	1	2	2	2	1	2	2	2	2	2		35	2	0	1	1	2,8	2	1	2016-06-04 12:45	2	2	2	2	3		2	1	1	2		17.1	2	2	2	2	1	2	1
1	53	1	1	2	1	0	2	2	2	2	2		19	1	0	1	1	1	1	1	2016-06-06 07:00	2	2	2	1	2	1	2	1	1	2		33.9	2	2	1		1	1	1
1	54	2	2	2	2	0	2	2	1	2	2		35	1	1	1	1	2	2	1	2016-06-06 23:50	2	2	1	2	1		2	2	2	2		13	1	2	2	3	1	2	1
1	33	2	1	1	1	0	2	2	2	2	2		17	1	0	1	1	1,8	1	1	2016-06-18 01:20	2	2	1	2	1	0	2	1	1	2		35.8	2	2	1		0	1	0
2	20	2	1	2	2	0	2	2	2	2	2		30	2	0	1	1	1,8	1	1	2016-06-19 01:00	2	2	2	2	3	1	2	2	2	2		16	2	2	1		1	1	1
2	57	2	1	1	1	0	2	2	2	2	2		32	1	0	1	1	1,8	1	1	2016-07-07 23:00	2	2	1	2	1	0	2	2	2	2		22	2	2	1		0	1	0
1	77	1	1	1	1	0	2	1	1	2	2		18	1	0	1	1	1,8	1	1	2016-07-05 01:20	2	2	1	2	1		2	1	1	2		23.7	2	2	2	1	1	2	1
1	52	1	1	1	1	0	1	2	2	2	2		12	1	0	1	1	1,8	1	1	2016-07-22 10:27	2	2	2	1	2	0	2	1	1	2		30.3	2	2	1		0	1	0
1	35	2	2	2	2	1	2	2	2	2	2		5	1	0	1	1	1,8	1	1	2016-08-13 02:30	2	2	1	2	1	0	2	2	2	2		37.4	2	2	1		0	1	0
2	83	1	1	1	2	0	2	1	2	2	2		31	2	0	1	1	2,8	2	1	2016-08-15 14:56	2	2	2	2	3		2	1	1	2		36	2	2	1		1	2	1
1	78	1	1	2	2	0	2	2	2	2	1		43	2	0	1	1	1	1	1	2016-08-23 03:43	2	2	2	2	3		2	2	2	2		13.6	1	2	2	2	1	2	1
1	40	2	1	2	1	0	2	2	1	2	2		17	2	0	1	1	1,8	1	1	2016-08-30 17:00	2	2	2	2	3		2	2	2	2		6	2	2	1		0	1	0
1	58	2	1	1	2	0	2	2	2	2	2		76	1	1	1	3	9	9	1	2016-09-01 21:09	1	2	1	2	1		1	2	2	2		9.3	2	2	2	3	1	2	1
1	67	1	1	1	2	0	2	2	2	2	2		22	2	0	1	1	1	1	1	2016-09-10 17:19	2	2	2	2	3	0	2	2	2	2		32.3	2	2	2	3	1	2	1
1	22	1	2	2	2	0	2	2	2	2	1		12	2	1	1	1	2	2	1	2016-09-21 20:35	2	2	2	2	3		2	2	2	2		21.1	2	2	2	2	1	2	1
2	64	2	2	2	2	0	2	2	2	2	2		37	2	0	1	1	2,8	2	1	2016-09-27 10:22	2	2	2	2	3		2	1	1	2		21.2	2	2	1		1	2	1
2	55	2	2	2	2	0	2	2	2	2	2		37	1	0	1	1	2,8	2	1	2016-10-01 14:47	2	2	1	2	1		2	2	2	2		24.2	2	2	2	2	1	2	1
1	36	2	2	1	1	0	2	2	2	2	2		70	1	1	1	1	1	1	1	2016-10-04 15:40	2	2	1	2	1		2	2	2	2		57.1	1	2	2	2	1	2	1
1	78	2	2	1	2	0	2	1	2	2	2		12	2	0	1	1	1,8	1	1	2016-11-04 16:50	2	2	2	2	3		2	2	2	2		38.5	2	2	1		1	2	1
1	76	1	1	1	2	0	2	1	1	2	2		65	2	0	1	1	2	2	1	2016-11-06 11:40	2	2	2	2	3		2	2	2	2		28.3	2	2	2	2	1	2	1
2	50	1	1	1	1	0	2	2	2	2	2		18	1	0	1	1	2	2	1	2016-11-13 08:30	2	2	1	2	1	0	2	1	1	2		21.9	2	2	1		0	1	0
1	75	1	1	1	2	0	2	2	2	2	2		24	2	0	1	1	1	1	1	2016-11-12 02:43	2	2	2	2	3	0	2	2	2	2		23.9	2	2	1		1	2	1
1	59	2	1	1	1	0	2	2	2	2	2		16	1	0	1	1	2,8	1	1	2016-11-28 17:08	2	2	1	2	1	1	2	1	1	2		22.4	2	2	1		0	1	0
1	59	2	1	1	1	0	2	1	1	2	2		21	1	0	1	1	1,8	1	1	2016-12-03 16:35	2	2	2	1	2	1	2	2	2	2		18.7	2	2	1		0	1	0
1	48	2	1	1	1	0	1	2	2	2	2		62	1	1	1	3	9	9	1	2016-11-01 10:03	1	2	2	1	2	1	2	2	2	2		10.7	1	2	2	1	1	2	1
1	67	1	1	1	1	1	1	1	2	2	2		37	1	0	1	1	1	1	1	2017-01-15 06:24	2	2	1	2	1	1	2	2	2	2		15.5	2	2	1		0	1	0
1	49	1	1	1	1	0	2	2	2	2	2		16	1	0	1	1	2,8	2	1	2017-01-19 10:58	2	2	1	2	1	0	2	2	2	2		22.7	2	2	1		0	1	0
1	57	2	2	2	2	0	2	2	2	2	2		33	2	0	0	1	1	1	2		2	2	2	2	3	0	2	2	2	2		1.1	2	1	2	3	1	2	1
1	62	1	1	1	2	0	2	2	1	2	2		45	2	1	0	1	1	1	1	2017-03-08 00:54	2	2	2	2	3		2	2	2	2		40.8	2	2	2	3	1	2	1
2	49	2	1	2	2	0	2	2	2	2	2		30	1	1	0	3	9	9	1	2017-03-15 20:00	1	2	1	2	1	0	1	2	2	2		7.9	2	1	2	2	1	2	1
1	58	2	1	1	1	0	2	1	2	2	2		22	1	0	1	1	1	1,8	1	2017-03-23 16:40	2	2	2	1	2	0	2	1	1	2		64.6	2	2	1		0	1	0
1	63	1	2	2	2	0	2	1	1	1	1		29	2	0	1	1	1	1	1	2017-04-14 21:05	2	2	2	2	3		2	2	2	2		15.2	2	2	1		1	2	1
1	36	2	1	1	2	0	2	2	2	2	2		49	2	1	1	1	1	1	1	2017-04-21 21:37	2	2	2	2	3	0	2	2	2	2		25.3	99	2	2	2	1	2	1
1	52	2	2	2	2	0	2	2	2	2	2		33	1	0	1	1	1	1	1	2017-05-21 04:10	1	2	2	1	2	1	2	1	1	2		27.4	2	2	1		0	1	0
2	78	2	1	2	1	0	2	1	2	2	2		12	1	0	1	1	1,8	1	1	2017-05-22 17:35	2	2	2	1	2	1	2	2	2	2		14.1	2	2	1		0	1	0
2	42	1	1	1	1	0	2	2	2	2	2		9	1	0	1	1	1,8	1	1	2017-05-28 09:22	2	2	1	2	1	0	2	1	1	2		18	2	2	1		0	1	0
1	51	2	1	2	2	0	2	2	2	2	2		15	2	1	1	1	1	1	1	2017-05-29 21:30	2	2	2	2	3		2	2	2	2		18.2	2	2	1		1	2	1
1	59	2	1	1	2	0	2	2	2	2	2		28	1	1	1	1	1,8	1	1	2017-06-02 23:50	2	2	1	2	1		2	1	1	2		13.5	99	2	2	2	1	2	1
1	80	2	2	1	1	0	2	2	2	2	2		41	1	0	1	1	1,8	1,8	1	2017-06-20 09:36	2	2	2	1	2	1	2	2	2	2		5.3	1	1	2	3	1	2	1
1	78	1	2	2	2	0	2	2	2	2	2		50	2	0	1	1	1,8	1	1	2017-06-25 21:15	2	2	2	2	3		2	2	2	2		23.8	2	2	1		1	2	1
1	69	2	2	2	2	0	1	2	1	2	2		8	1	0	1	1	1	1	1	2017-07-08 21:27	2	2	2	1	2	1	2	1	1	2		20.2	2	1	1		0	1	0
1	52	2	2	2	2	0	2	2	2	2	2		33	1	1	1	1	2,8	2	1	2017-07-22 12:19	2	2	1	2	1		2	1	1	2		15.8	2	2	1		1	2	1
1	38	2	1	1	1	0	2	2	2	2	2		32	1	0	1	1	1,8	1	1	2017-07-22 21:10	2	2	1	2	1		2	1	1	2		14.1	2	1	1		1	1	1
1	68	1	2	2	2	0	2	2	2	2	1		40	2	0	1	1	1	1	1	2017-08-06 14:20	2	2	2	2	3		2	1	1	2		13.4	1	2	1		1	2	1
1	61	1	1	1	2	0	2	1	2	2	2		45	1	0	1	1	1	1	1	2017-08-10 11:47	2	2	2	1	2	1	2	1	1	2		15.2	2	2	1		0	1	0
1	57	1	2	2	2	0	2	1	1	2	2		57	2	0	1	1	2	2	1	2017-08-19 23:49	2	2	2	2	3		2	1	1	2		34.6	2	2	2	2	1	2	1
1	54	1	1	1	1	0	2	2	2	2	2		45	1	0	1	1	1,8	1,8	1	2017-08-20 17:23	2	2	2	1	2	1	2	1	1	2		10	2	2	1		1	1	1
2	36	1	2	2	2	0	2	2	2	2	2		62	2	1	1	1	2	2	1	2017-08-23 05:42	2	2	2	2	3	0	2	2	2	2		23.7	2	2	2	2	1	2	1
1	76	1	1	1	1	0	2	1	2	1	2		16	1	0	1	1	1	1	1	2017-08-25 21:05	2	2	2	1	2	1	2	2	2	2		9.7	2	2	1		0	1	0
2	36	1	2	2	2	0	2	2	2	2	2		52	2	1	1	1	2	2	1	2017-08-29 12:09	2	2	2	2	3	0	2	1	1	2		26.6	1	2	2	2	1	2	1
1	78	1	2	1	2	0	1	1	1	1	2		45	1	0	1	1	1	1	1	2017-09-02 21:41	2	2	2	2	3		2	2	2	2		0.7	2	1	2	3	1	2	1
1	59	1	2	2	2	0	2	2	2	2	2		51	2	1	1	1	2,8	2	1	2017-09-01 20:35	2	2	1	2	1	0	2	1	1	2		32.8	2	2	2	2	1	2	1
1	60	2	1	1	2	0	1	1	2	2	2		17	1	0	1	1	1	1	1	2017-10-21 09:30	1	2	1	2	1	1	2	1	1	1		34.4	1	2	2	3	1	2	1
2	71	1	2	2	2	0	2	2	1	2	2		35	2	0	1	1	1,8	1	1	2017-10-21 20:30	2	2	2	2	3		2	1	1	2		26.3	2	2	1		1	2	1
1	84	2	2	2	2	0	2	2	2	2	2		18	2	1	1	1	1,8	1	1	2017-10-27 20:11	2	2	2	2	3		2	1	1	2		9.5	2	2	2	3	1	2	1
2	56	1	1	2	2	0	2	2	2	2	1		42	2	0	1	1	1,8	1	1	2017-11-06 16:35	2	2	2	2	3		2	2	2	2		13.5	1	2	1		1	1	1
1	55	2	1	1	1	0	2	2	2	2	2		33	1	0	1	1	1,8	1,8	1	2017-11-22 00:43	2	2	1	2	1	0	2	2	2	2		16.5	2	2	1		0	1	0
1	39	1	2	2	2	0	2	2	2	2	2		49	2	0	1	1	1	1	1	2017-11-22 17:27	2	2	2	2	3	0	2	1	1	2		34.9	2	2	1		1	2	1
1	24	2	1	1	2	0	2	2	2	2	2		54	2	1	1	1	1	1	1	2017-12-15 23:00	2	2	2	2	3		2	2	2	2		31.4	2	2	1		1	2	1
1	52	1	1	2	1	0	2	2	2	2	2		9	1	0	1	1	1,8	1	1	2017-12-19 11:49	2	2	2	1	2	1	2	2	2	2		19.5	2	2	1		0	1	0
1	45	2	2	2	2	0	2	2	2	2	2		71	2	0	1	1	2	2	1	2017-12-23 08:45	2	2	2	2	3		2	2	2	2		20.7	2	2	1		1	2	1
1	77	2	2	2	2	0	2	1	2	2	2		40	2	0	1	1	1	1	1	2017-12-27 10:22	2	2	2	2	3		2	1	1	2		33.6	1	2	2	2	1	2	1
1	25	2	1	2	2	0	2	2	2	2	2		27	1	0	1	1	1,8	1	1	2018-01-01 06:16	2	2	1	2	1	0	2	2	2	2		4.9	2	2	1		0	1	0
2	77	1	2	1	2	0	2	1	1	2	2		28	1	0	1	1	1	1	1	2018-01-06 22:20	2	2	2	1	2	1	2	2	2	2			2	2	2	2	1	2	1
1	47	1	1	1	2	0	2	2	2	2	2		40	2	0	1	1	1	1	1	2018-01-11 20:05	2	2	1	2	1		2	2	2	2		12.2	1	1	2	3	1	2	1
2	82	2	2	2	2	0	2	1	2	2	2		20	2	0	1	1	1,8	1	1	2018-01-19 14:40	2	2	2	2	3		2	2	2	2		15.1	2	2	1		1	2	1
1	24	1	2	1	2	0	2	2	2	2	2		38	2	0	1	1	1	1	1	2018-01-19 04:59	2	2	2	2	3	0	2	1	1	2		26.2	2	2	2	2	1	2	1
2	60	1	1	1	2	0	2	1	2	1	2		46	2	1	1	1	1	1	1	2018-01-25 15:55	2	2	2	2	3		2	2	2	2		27.6	2	2	1		1	2	1
2	47	2	2	2	2	0	2	2	2	2	2		30	2	1	1	1	2	2	1	2018-01-26 18:35	2	2	2	2	3		2	2	2	2		4.4	2	1	2	3	1	2	1
1	79	2	2	1	2	0	2	1	2	2	2		50	2	0	1	1	1	1	1	2018-02-01 16:41	2	2	2	2	3		2	2	2	2		1.9	1	1	2	3	1	2	1
1	72	1	2	1	2	0	2	2	2	2	2		60	2	1	1	1	1	1	1	2018-02-13 03:55	2	2	2	2	3		2	2	2	2		11.6	2	1	2	2	1	2	1
2	65	2	2	2	2	0	2	1	1	1	2		36	2	1	1	1	1	1	1	2018-02-17 16:50	2	2	2	2	3		2	1	1	2		7	1	2	2	3	1	2	1
2	52	1	2	2	2	0	2	2	2	2	2		26	2	0	1	1	2	2	1	2018-02-19 17:45	2	2	2	2	3		2	2	2	2		12.4	2	2	2	2	1	2	1
1	60	2	1	1	1	0	2	2	2	2	2		14	1	0	1	1	1	1	1	2018-03-06 21:49	2	2	2	1	2	1	2	2	2	2		3.6	2	2	1		0	2	1
2	74	1	2	2	2	0	2	1	2	2	2		52	2	1	1	1	2,8	2	1	2018-03-09 01:44	2	2	2	2	3		2	2	2	2		19.6	1	2	2	2	1	2	1
2	73	1	2	1	2	0	2	2	1	2	2		42	2	1	1	1	2	2	1	2018-03-14 01:14	2	2	2	2	3		2	1	1	2		34.7	2	2	2	2	1	2	1
1	60	2	2	1	1	0	1	2	2	2	2		37	1	1	1	1	1	1	1	2018-03-18 16:35	2	2	2	2	3		2	2	2	2		10	2	2	1		1	2	1
2	42	2	2	2	2	0	2	2	2	2	2		71	2	0	1	1	1	1	1	2018-03-24 20:00	2	2	2	2	3		2	2	2	2		17.8	2	1	2	2	1	2	1
2	63	2	1	2	1	0	2	1	2	2	2		49	1	0	1	1	1,8	1	1	2018-05-09 02:30	2	2	2	1	2	1	2	2	2	2		7.5	2	2	1		1	1	1
1	79	1	2	2	2	0	2	2	2	2	2		41	2	0	1	1	1	1	1	2018-05-26 00:08	2	2	2	2	3		2	2	2	2		15.3	2	2	2	2	1	2	1
1	71	1	2	2	2	0	2	2	2	2	2		41	2	0	1	1	2	2	1	2018-05-28 11:18	2	2	2	2	3		2	1	1	2		18.9	2	2	2	2	1	2	1
1	41	1	2	2	2	0	2	2	2	2	2		46	2	1	1	1	1	1	1	2018-06-30 02:59	2	2	2	2	3	0	2	2	2	2		32.7	2	2	2	2	1	2	1
1	37	1	2	1	2	1	2	2	2	2	2		60	2	0	1	1	1	1	1	2018-07-08 21:45	2	2	2	2	3		2	2	2	2		28.2	2	1	2	2	1	2	1
1	56	2	2	1	2	0	2	2	2	2	2		81	2	0	1	1	1	1	1	2018-07-17 21:05	2	2	2	2	3		2	2	2	2		18.1	2	1	1		1	2	1
1	42	1	2	2	2	0	2	2	2	2	2		32	2	0	1	1	1	1	1	2018-07-31 05:57	2	2	2	2	3		2	2	2	2		32.4	2	2	2	2	1	2	1
1	30	2	1	1	2	0	2	2	2	2	2		8	2	0	1	1	1	1	1	2018-07-31 18:23	2	2	2	2	3		2	2	2	2		25.6	2	2	1		0	1	0
1	44	1	2	1	2	0	2	2	2	2	2		57	2	0	1	1	2,8	2	1	2018-08-01 16:08	2	2	2	2	3		2	2	2	2		15.3	2	2	2	2	1	2	1
2	71	2	2	1	2	0	2	1	1	2	1		25	2	0	1	1	1	1	1	2018-01-15 10:50	2	2	2	2	3		2	2	2	2		13.5	1	2	1		1	2	1
1	60	2	2	1	2	0	2	2	2	1	2		28	2	0	1	1	1	1	1	2018-08-09 21:35	2	2	2	2	3	0	2	2	2	2		36	2	2	2	2	1	2	1
1	61	2	1	1	2	0	2	2	1	2	2		85	1	1	1	3	9	9	1	2018-08-10 13:18	1	2	2	1	2	1	2	1	2	2		22.8	1	2	2	3	1	2	1

2	78	1	1	2	2	0	1	2	2	2	2		61	2	0	1	1		1	1	1	2018-08-11 13:54	2	2	2	2	3	0	2	1	1	2		24.5	2	2	2	2	1	2	1
2	83	2	2	1	1	0	2	2	2	2	2		11	2	0	1	1	1,8		1	1	2018-08-15 22:05	2	2	2	2	3		2	2	2	2		13.5	2	2	1		1	2	1
2	68	2	2	2	2	0	2	1	1	1	2		19	1	0	1	1		1	1	1	2018-08-28 20:00	2	2	2	2	3	1	2	1	1	2		19.6	2	2	1		0	1	0
1	49	2	1	1	2	0	2	2	2	2	2		61	1	1	1	3		9	9	1	2018-09-07 19:35	1	2	2	1	2	1	1	2	2	2		50.7	1	1	2	3	1	2	1
1	55	1	2	1	1	0	2	1	2	2	2		21	1	1	1	1		1	1	1	2018-09-13 15:40	2	2	2	1	2	1	2	1	1	2		17.9	2	2	1		1	1	0
1	62	1	1	1	2	1	2	1	2	2	2		78	1	0	1	1		1	1	1	2018-09-18 10:00	2	2	1	2	1	0	2	1	1	2		31.4	2	2	1		1	2	1
1	72	1	2	2	2	0	1	2	2	2	2		57	2	0	1	1		1	1	1	2018-09-25 08:50	2	2	2	2	3		2	2	2	2		29.6	2	2	2	2	1	2	1
1	55	1	1	2	2	0	2	2	2	2	2		35	2	0	1	1		1	1	1	2018-10-06 17:30	2	2	1	2	1	0	2	2	2	2		9.6	2	2	2	2	1	2	1
1	32	1	1	1	1	0	2	2	2	2	2		14	1	0	1	1		1	1	1	2018-10-15 05:21	2	2	1	2	1	0	2	1	1	2		27.5	2	2	1		0	1	0
1	38	1	2	1	2	0	2	2	2	2	2		34	2	0	1	1	2,8		2	1	2018-10-17 01:53	2	2	2	2	3		2	2	2	2		35.8	2	2	2	2	1	2	1
1	79	2	2	2	1	0	2	2	2	2	2		20	2	0	1	1	1,8		1	1	2018-10-25 05:40	2	2	2	2	3	0	2	2	2	2		6.6	2	2	1		1	1	0
1	57	2	1	1	2	0	2	2	2	2	2		22	2	0	1	1	1,8		1	1	2018-10-27 23:25	2	2	1	2	1		2	1	1	2		26.1	2	2	1		1	2	1
1	46	2	2	2	2	0	2	2	2	2	2		6	2	1	1	1		1	1	1	2018-11-01 04:50	2	2	2	2	3		2	2	2	2		6.7	2	2	1		0	1	0
1	75	1	2	2	2	0	2	1	1	2	2		51	2	0	1	1	1,8		1	1	2018-11-27 13:50	2	2	2	2	3		2	2	2	2		20.2	2	2	1		1	2	1
2	54	2	2	2	2	0	2	2	2	2	2		34	2	0	1	1	1,8		1	1	2018-11-29 16:50	2	2	1	2	1		2	1	2	1		12.9	2	2	2	3	1	2	1
2	56	1	1	1	1	0	2	2	2	2	2		17	1	0	1	1	1,8		1	1	2018-12-08 20:26	2	2	1	2	1	1	2	1	1	2		34.5	2	2	1		0	1	0
2	77	2	2	1	2	0	2	2	2	2	2		6	2	0	1	1	1,8		1	1	2018-12-10 16:38	2	2	2	2	3	0	2	1	1	2		24.2	2	2	1		0	1	0
2	81	1	1	1	2	0	2	1	1	2	2		33	1	0	1	1		1	1	1	2018-12-29 14:05	2	2	2	2	3		2	2	2	2		6.2	2	2	2	2	1	2	1
1	76	1	2	1	2	0	2	2	2	2	2		51	2	0	1	1		1	1	1	2018-12-31 16:45	2	2	2	2	3		2	2	2	2		5.1	2	2	1		1	2	1
1	26	1	1	1	2	0	2	2	2	2	2		39	2	0	0	1		1	1	1	2015-12-26 11:39	2	2	2	2	3		2	1	1	2		25.5	1	1	2	3	1	2	1
1	64	1	1	1	2	1	2	2	2	2	2		39	2	0	0	1		1	1	1	2016-01-15 17:10	2	2	2	2	3	0	2	2	2	2		35.4	2	1	2	3	1	2	1
1	82	1	2	2	2	0	2	2	2	2	2		23	2	0	0	1		1	1	1	2016-02-04 13:53	2	2	2	2	3		2	2	2	2		22	2	2	2	3	1	2	1
1	71	2	1	1	2	0	2	1	1	2	2		34	1	1	0	1		1	1	1	2016-02-22 20:00	2	2	2	2	3		2	1	1	2		36.1	2	2	2	2	1	2	1
1	57	2	1	1	1	0	2	1	2	2	2		12	1	0	0	1		1	1	1	2016-02-21 21:35	2	2	2	1	2	0	2	2	2	2		63.7	2	2	1		0	1	0
2	38	1	2	2	2	0	2	2	2	2	2		27	2	0	0	1		1		1	2016-02-26 13:01	2	2	2	2	3		1	2	2	2		3.1	1	2	2	3	1	2	1
1	80	1	2	1	2	0	2	2	2	2	2		33	2	1	1	2		4	4	1	2016-02-26 15:35	2	2	2	2	3		2	1	1	2		18.5	1	2	2	3	1	2	1
1	68	1	1	2	1	0	2	1	2	2	2		15	1	0	0	1		1	1	1	2016-05-17 09:02	2	2	1	2	1	1	2	1	1	2		20.8	1	1	1		1	1	1
1	42	2	1	2	2		2	2	2	2	2		66	2	1	0	1		1		2		2	2	2	2	3		2	2	2	2		1	2	1	2	2	1	2	1
1	56	1	1	1	1	0	2	1	2	2	2		77	1	0	1	1		1	1	1	2016-05-20 22:00	2	2	2	1	2	1	2	2	2	2		13.8	2	2	1		0	1	0
1	47	2	1	1	1	0	2	2	2	2	2		43	1	0	0	1	1,6		1	1	2016-05-21 14:17	2	2	2	1	2	1	2	2	2	2		11.7	2	2	1		0	1	0
1	76	1	1	1	2	0	2	1	2	2	2		68	2	0	0	1		1	1	1	2016-06-11 14:50	2	2	2	2	3		2	1	1	1		30.4	2	2	2	2	1	2	1
1	60	2	2	2	2	0	2	1	2	2	2		50	2	0	0	1	1,6		1	1	2016-06-18 00:45	2	2	2	2	3		2	1	1	1		21.2	2	2	1		1	1	1
2	68	1	2	1	2		2	2	2	2	2		50	1	1	1	1		1	1	1	2016-07-07 16:50	2	2	2	2	3	0	2	2	2	2		29.9	2	2	1		1	2	1
1	31	2	1	2	2		2	2	2	2	2		53	2	0	0	1		1		2		2	2	2	2	3		2	2	2	2		20	2	2	2	2	1	2	1
1	74	1	1	2	2	0	2	2	2	2	2		94	1	0	0	1		1	1	1	2016-08-10 01:04	2	2	2	2	3		2	2	2	2		65.2	2	2	2	2	1	2	1
1	53	2	1	1	2	0	2	2	2	2	2		16	2	0	0	1		1	1	1	2016-08-15 14:20	2	2	2	2	3		2	1	1	2		19.2	2	1	1		1	2	1
1	33	2	1	2	2	0	2	2	2	2	2		39	2	0	0	1		1	1	1	2016-09-25 19:33	2	2	2	2	3		2	2	2	2		45.5	2	2	2	2	1	2	1
1	71	2	1	1	2		1	1	1	2	2	1	24	1	0	0	1		1	1	1	2016-10-16 17:39	2	2	2	2	3	1	2	2	2	2		13	2	2	2	3	1	2	1
2	76	1	1	1	2	0	2	1	2	2	2		32	1	1	0	1		1		1	2016-10-29 19:50	2	2	2	2	3	1	2	2	2	2		1.4	2	2	2	3	1	2	1
2	60	2	1	1	2		2	1	1	2	2		47	1	0	0	1		1	1	1	2016-11-01 14:32	2	2	2	2	3		2	2	2	2		33.4	2	99	2	2	1	2	1
1	67	2	1	1	1	0	2	2	2	2	2		20	1		1	1		1	1	1	2016-11-19 01:00	2	2	2	2	3	0	2	1	1	2		18.8	2	2	2	1	1	2	1
2	69	1	1	1	1	0	2	2	2	2	2		20	1	0	0	1		1	1	1	2016-11-27 06:30	2	2	2	1	2	1	2	1	2	1		11.8	2	1	1		0	1	0
2	76	2	1	2	1	0	2	1	2	2	2		10	1	0	1	1		1	1	1	2016-11-29 21:10	2	2	2	2	3		2	1	1	2		34.6	2	2	2	3	1	2	1
1	82	1	1	2	2	0	2	1	1	2	2		32	1	0	0	1		1	1	1	2017-01-19 00:30	2	2	2	2	3		2	1	1	2		15.8	2	2	2	3	1	2	1
1	74	2	2	2	1	0	1	1	1	2	2		22	1	0	0	1		1	1	1	2017-02-01 16:00	2	2	2	1	2	1	2	1	1	2		13.5	2	2	1		0	1	0
1	69	2	1	2	2	0	1	1	1	2	1		29	1	0	0	1		1	1	1	2017-03-14 21:48	2	2	2	2	3	0	2	1	1	2		12.4	1	2	2	2	1	2	1
1	23	1	2	2	2		2	2	2	2	2		37	2	0	0	1		1		2		2	2	2	2	3		2	2	2	2		1.4	2	2	2	1	1	2	1
1	75	2	1	1	2	0	2	2	2	2	2		21	1	0	0	1		1	1	1	2017-01-05 14:41	2	2	2	2	3		2	2	2	2		26.8	1	2	2	3	1	2	1
2	90	1	1	1	2		2	1	2	2	2		29	1	0	1	1		1	1	1	2017-01-08 21:22	2	2	2	2	3		2	1	1	2		21.7	2	2	2	3	1	2	1
1	70	1	1	2	2		2	2	1	2	2		27	1	0	0	1		1	1	1	2017-03-12 20:21	2	2	2	2	3		2	2	2	2		1.4	2	1	2	3	1	2	1
2	42	2	1	2	1	0	2	2	2	2	2		52	2	1	1	1		1		1	2017-05-17 15:49	2	2	2	2	3		2	1	1	2		0.4	2	2	2	3	1	2	1
1	55	1	1	1	1	0	2	2	2	2	2		40	1		0	1		1	1	1	2017-07-06 11:40	2	2	2	1	2	0	2	2	2	2		13.9	2	2	1		0	1	0
1	47	2	1	1	2	1	2	2	2	2	2		18	2	0	0	1		1	1	1	2017-07-13 10:09	2	2	2	2	3		2	2	2	2		13	2	2	1		0	1	0
1	39	2	1	1	1	0	2	1	2	2	2		54	1	0	0	1		1	1	1	2017-07-18 17:05	2	2	1	2	1	0	2	1	1	2		10.8	2	1	1		0	1	0
1	54	2	1	1	1	0	2	2	2	2	2		11	1	0	0	1		1	1	1	2017-10-24 22:45	2	2	2	1	2	0	2	1	2	2		31.3	2	2	1		0	1	0
1	62	1	1	2	2	0	2	1	1	2	2		32	1	0	0	1		1	1	1	2017-12-01 12:50	2	2	2	1	2	1	2	1	1	2		48.9	2	1	1		1	1	1
1	56	1	2	1	2	0	2	2	2	2	2		78	2	0	0	1		1	1	1	2018-01-17 20:20	2	2	2	2	3		2	1	1	2		31.4	2	2	2	3	1	2	1
2	81	2	1	1	1	0	2	2	2	2	2		33	1	0	0	1		1	1	1	2018-01-23 21:55	2	2	2	2	3	1	2	1	1	2		20.3	2	1	2	4	1	2	1
2	57	1	2	1	2	1	2	2	2	2	2		43	2	0	0	1		1	1	1	2018-02-15 11:50	2	2	2	2	3		2	2	2	2		26.3	2	2	2	2	1	2	1
1	61	2	1	2	1	0	2	1	2	2	2		70	2	0	0	1		1	1	1	2018-03-04 13:40	2	2	2	2	3		2	2	2	2		38.1	2	2	2	3	1	2	1
1	80	1	1	2	2	0	2	2	2	2	2		35	1	0	0	1		1	1	1	2018-03-07 21:32	2	2	2	2	3		2	1	1	2		5.5	2	2	2	1	1	2	1
2	66	1	2	1	2	0	2	1	2	2	2		50	2	0	0	1		1	1	1	2018-03-23 18:51	2	2	2	2	3		2	1	1	2		100	2	2	2	4	1	2	1
1	46	1	1	2	2	0	2	2	2	2	2		29	2	1	0	1		1	1	1	2018-03-31 09:34	2	2	2	2	3		2	1	1	2		7.3	2	2	1		1	1	1
1	48	1	2	2	2	0	2	2	2	2	2		33	2	1	0	1		1	1	1	2016-05-26 01:30	2	2	2	2	3		2	2	2	2		50.8	2	2	2	3	1	2	1
2	70	1	2	2	2	0	2	1	2	2	2		11	2	0	0	1		1	1	1	2015-10-01 13:52	2	2	2	2	3	0	2	1	1	2		23.7	2	2	1		1	1	1
1	78	1	1	2	2	0	2	1	1	2	2		59	1	0	0	1		1	1	1	2018-01-05 02:00	2	2	2	1	2		1	2	2	2		93.2	1	1	2	1	1	2	1
1	69	1	1	2	2	0	2	1	1	2	2		46	1	0	0	1		1	1	1	2018-05-10 03:50	2	2	2	2	3		2	1	1	2		6.9	2	2	2	4	1	2	1
1	73	1	2	2	2	0	1	2	2	1	2		46	1	0	0	1		1	1	2		2	2	2	2	3		2	1	1	2		1.8	2	1	2	3	1	2	1
1	73	2	2	1	1	0	1	1	1	2	2		17	1	0	1	1		1	1	1	2018-04-21 19:30	2	2	2	2	3		2	2	2	2		12.9	1	2	1		0	1	0
1	40	1	1	1	1	0	2	1	2	2	2		46	1	0	0	1		1	1	1	2018-06-22 10:05	2	2	2	1	2		2	1	1	2		100	2	2	2	1	1	2	1
1	55	2	1	2	1	0	2	2	1	2	2		51	1	1	0	1		1	1	1	2018-07-04 17:32	2	2	2	1	2	1	2	2	2	2		14.1	2	2	1		0	1	0
1	48	2	2	1	1	0	2	1	1	2	2		34	1	1	0	1		1	1	1	2018-08-31 18:50	2	2	2	2	3		2	1	1	2		19.4	2	2	2	3	1	2	1
1	42	2	1	2	2	0	2	2	2	2	2		30	1	0	0	1		1	1	1	2018-09-20 13:00	2	2	2	1	2	1	2	2	2	2		9.7	2	2	1		0	1	0
2	40	1	1	2	2	0	2	2	1	2	1		35	1	0	1	1		1	1	1	2018-11-04 13:45	2	2	2	2	3	1	2	1	1	2		21.6	1	2	2	3	1	2	1
1	68	2	1	1	1		2	2	2	2	2		15	1	0	0	1		2	2	1	2016-05-07 15:01	2	2	1	2	1	1	2	1	1	2		9.7	2	2	1		1		
1	56	2	1	2	2	0	2	1	2	2	2		43	2		0	1		2	2	1	2016-05-16 19:55	2	2	1	2	1	1	2	1	1	2		22.9	2	2	1		0		0
1	67	2	1	1	2	0	1	1	1	1	2		17	1	0	0	1		2	2	1	2016-05-18 18:51	2	2	1	2	1		2	2	2	2			2	2	2	2	1	2	1
2	43	2	1	2	2	0	2	2	1	2	2		26	2	0	0	1		2	2	1	2016-05-31 21:04	2	1	2	2	3		2	2	2	2		4.2	2	2	1		1	2	1
2	41	1	1	1	1	0	2	2	2	2	2		15	1	0	0	1		2	2	1	2016-06-04 23:14	2	2	1	2	1	0	2	2	2	2		6.7	2	2	1		0	1	0
1	79	1	1	2	2	0	1	1	1	2	2	1	39	1	0	0	1		2	2	1	2016-06-06 19:49	2	2	2	2	3	1	2	1	1	2		18.4	1	2	1		1	2	1
1	47	2	1	2	1	0	2	1	2	2	2		13	1		0	1		2	2	1	2016-06-14 18:17	2	2	2	1	2	0	2	2	2	2		6.4	2	2	1		0	1	0
1	47	2	1	1	1		1	2	2	2	2		33	1	0	0	1		2	2	1	2016-06-17 12:59	2	2	2	2	3	0	2	2	2	2		12.6	2	2	1		0	1	0
1	18	1	1	1	1	0	2	2	2	2	2		26	1	0	0	1		2	2	1	2016-09-12 03:50	2	1	2	2	3	0	2	1	1	2		17.9	2	2	1		0	1	0
2	41	2	1	1	1	0	2	2	2	2	2		20	1	0	0	1		2	2	1	2016-10-11 22:43	2	2	1	2	1	1	2	1	1	2		11.7	2	2	1		0	1	0
1	50	2	1	2	1	0	2	1	1	2	2		15	1		0	1		2	2	1	2016-10-14 16:48	2	2	1	2	1	0	2	1	2	2		11.4	2	2	1		0	1	0
1	68	2	1	1	2	0	2	2	2	2	2		6	2	0	0	1		2	2	1	2016-10-21 17:26	2	1	2	2	3		2	2	2	2		4.9	2	2	1		1	2	1
1	72	2	1	1	2	0	1	1	1	2	2		10	1		0	1		2	2	2		2	2	1	2	1		2	2	2	2		6.1	2	1	2	1	1	2	1
1	40	2	1	2	2	0	2	2	2	2	2		8	2	1	0	1		2	2	1	2016-12-20 16:06	2	2	2	2	3		2	1	1	1		25.8	2	2	1		0	1	0
1	74	1	1	2	1	0	2	1	2	2	2		25	1	0	0	1		2	2	1	2017-01-01 22:05	2	2	2	2	3	1	2	2	2	2		11.6	2	1	1		0	1	0
1	63	2	1	2	1	0	2	1	2	2	2		52	1	0	0	1		2	2	1	2017-01-10 17:10	2	1	2	2	3		2	1	1	2		9.9	2	1	2	1	1	2	1
1	28	2	1	2	1	0	2	2	2	2	2		22	1	1	0	1		2	2	1	2017-01-06 18:32	2	2	1	2	1	0	2	1	2	2		9.6	2	2	1		0	1	0
2	82	1	1	2	2	0	1	1	1	2	1	1	41	2	1	0	1		2	2	1	2017-01-08 15:28	2	2	2	2	3		2	2	2	2			1	2	2	1	1	2	1
1	78	2	1	1	2	0	2	2	1	1	2		20	2	0	0	1		2	2	1	2017-01-17 16:49	2	2	2	2	3		2	2	2	2		19.9	2	2	2	1	1	2	1
1	55	1	2	2	2	0	2	1	2	2	2		46	2	0	0	1		2	2	1	2017-03-02 02:17	2	2	2	2	3		2	1	1	2		6.9	2	2	1		1	2	1
1	45	1	1	1	1	0	2	2	2	2	2		10	1	0	0	1		2	2	1	2017-03-30 20:25	2	2	2	1	2	0	2	2	2	2			2	2	1		0	1	0
1	84	1	1	1	2	0	2	1	1	2	2		59	2	1	0	1		2	2	1	2017-04-13 16:50	2	2	2	2	3		2	2	2	2			2	1	2	1	1	2	1
1	52	1	2	2	2	0	2	2	2	2	2		44	1	1	0	1		2	2	1	2017-04-20 23:14	1	2	2	1	2	1	1	2	2	2		0	2	1	2	1	1	2	1
2	55	2	1	2	2	0	2	2	1	2	2		44	2	0	0	1		2	2	1	2017-05-02 21:27	2	1	2	2	3		2	2	2	2		12.7	2	2	2	1	1	2	1
1	61	2	1	1	2	0	2	1	1	2	1		22	2		0	1		2	2	1	2017-05-09 03:52	2	1	2	2	3		2	2	2	2		11.6	1	2	2	3	1	2	1
1	76	2	1	1	2	1	2	2	2	2	2		53	2	0	0	1		2	2	1	2017-05-19 21:02	2	2	2	2	3	1	2	1	1	2		20.2	2	1	2	2	1	2	1
1	60	2	1	1	1	0	1	2	2	2	2	1	9	1		0	1		2	2	1	2017-06-03 01:24	2	2	2	1	2	1	2	2	2	2		17.1	2	2	1		0	1	0
1	57	1	1	2	1	0	1	1	1	2	2		28	1	0	0	1		2	2	1	2017-06-05 04:00	2	1	2	1	2	0	2	2	2	2		16.2	2	2	1		0	1	0
1	65	1	1	1	1	0	2	2	2	2	2		40	1	0	0	1		2	2	1	2017-06-19 14:21	2	2	2	1	2		2	2	2	2		19.4	2	2	1		1	1	1
2	55	2	1	1	2	1	1	1	1	1	1		18	2	0	0	1		2	2	1	2017-07-29 03:42	2	2	2	2	3	1	2	1	2	2		16.7	2	2	1		1	2	1
2	80	1	1	2	2	0	2	1	1	2	1		18	2		0	1		2	2	1	2017-08-05 01:56	2	1	2	2	3		2	2	2	2		16.9	1	2	2	3	1	2	1
2	26	1	1	2	1	0	2	2	2	2	2		55	2		0	3		9	9	1	2017-09-30 17:45	2	1	2	2	3		2	2	2	2		10.3	2	2	1		1	2	1
2	75	1	1	1	2	0	2	1	1	1	1		25	1		0	1		2	2	1	2017-10-09 14:47	2	2	2	1	2	1	2	2	2	2		18.3	1	1	2	1	1	2	1
1	31	1	1	1	1	0	2	2	2	2	2		14	1	0	0	1		2	2	1	2017-11-09 09:40	2	2	1	2	1	0	2	1	1	2		5.6	2	2	1		0	1	0
1	56	1	1	1	1	0	1	1	2	2	2		22	1		0	1		2	2	1	2017-11-26 14:20	2	2	1	2	1	0	2	1	1	2		16	2	2	1		0	1	0
1	84	2	1	1	1	0	1	2	2	2	1		13	1	0	0	1		2	2	1	2017-12-16 17:57	2	2	2	1	2	1	1	1	1	2		19.2	2	2	1		0	2	1
2	73	1	1	2	2	0	2	2	1	2	1		8	1	0	0	1		2	2	1	2018-01-12 23:30	2	2	2	2	3		2	1	1	2		17.2	1	2	1		1	2	1
1	67	1	1	2	2	0	2	2	2	2	2		87	1	1	0	1		2	2	1	2018-01-23 17:11	2	2	1	2	1	0	1	1	1	2		18.8	2	1	1		1	1	1
1	54	1	2	2	2	0	2	2	2	2	2		44	2	1	0	1		2	2	1	2018-03-19 00:36	2	2	2	2	3		2	1	1	2		12.7	2	2	2	2	1	2	1
2	74	1	1	1	2		2	1	2	1	2		40	1		0	1		2	2	1	2018-03-28 21:28	2	2	2	2	3		2	2	2	2		4.5	2	2	1		1		
1	79	2	1	1	2	0	1	1	1	2	2		13	2	0	0	1		2	2	1	2018-04-03 14:25	2	1	2	2	3		2	1	2	2		10.4	2	2	2	1	1	2	1

2	74	1	1	1	1	0	2	1	1	1	1		15	1		0	1	2	2	1	2018-04-07 14:15	2	1	2	2	3	1	2	2	2	2		7.5	2	2	1		0	1	0
2	81	1	1	1	2	0	2	1	2	2	2		40	1	0	0	1	2	2	1	2018-04-24 16:41	2	2	2	1	2	0	2	2	2	2		15.5	2	1	2	1	1	2	1
1	50	1	1	1	1	0	2	1	2	2	2		38	1	0	0	1	2	2	1	2018-05-08 14:50	2	2	1	2	1	0	2	2	2	2		16	2	2	1		0	1	0
2	35	1	1	1	2	0	2	2	2	2	2		24	2	1	1	1	2	2	1	2018-05-14 03:49	2	2	2	2	3		2	2	2	2		0	2	1	2	2	1	2	1
1	59	1	1	2	1	1	2	1	1	2	2		1	1	1	0	1	2	2	1	2018-05-30 09:34	2	2	1	2	1		1	1	2	2		1.6	2	1	2	1	1	2	1
1	63	1	1	2	1	0	2	1	2	2	2		18	1	0	0	1	2	2	1	2018-06-07 20:56	2	2	1	2	1	0	1	2	2	2		6.8	2	2	1		0	1	0
1	72	2	1	2	2		2	2	2	2	2		35	1	0	0	1	2	2	1	2018-06-08 16:08	2	2	2	2	3		99	2	2	2		19.8	2	2	1		1		
2	65	1	1	2	2	0	2	2	2	2	2		45	1	1	0	1	2	2	1	2018-06-10 01:54	2	2	2	2	3	0	2	2	2	2		10.3	2	1	2	1	1	2	1
1	52	1	1	1	1	0	2	2	2	2	2		32	1	1	0	1	2	2	1	2016-07-22 20:55	1	2	2	1	2	1	2	1	2	1		29.6	2	2	1	4	0	1	0
1	60	2	1	1	1	0	2	2	2	2	2		15	1	0	0	1	2	2	1	2018-08-08 22:23	2	2	1	2	1	0	2	1	2	2		2.7	2	1	1		0	1	0
1	83	2	1	1	1	0	2	2	2	2	2		53	1	0	0	1	2	2	1	2018-09-03 20:01	2	2	2	1	2	0	1	1	1	2		15	2	2	1		0	1	0
1	75	2	1	2	2	0	1	2	1	1	1	1	22	1	0	0	1	2	2	1	2018-10-14 04:22	2	2	2	1	2	1	2	2	2	2		10.1	1	1	2	1	1	2	1
1	64	2	2	2	2	0	1	1	1	2	2		15	1	0	0	1	2	2	1	2018-10-26 00:13	2	2	2	2	3	1	2	1	1	2		17.6	1	1	2	1	1	2	1
2	69	2	1	2	1	0	2	2	2	1	2		10	1	0	0	2	4	4	1	2016-07-05 21:00	2	2	1	2	1	0	2	2	2	2		100	2	2	1		0	1	0
1	55	1	2	2	2	0	2	2	2	2	2		42	2	1	0	2	4	4	2		2	2	2	2	3		2	2	2	2		0.8	2	1	2	2	1	2	1
2	55	2	2	2	1	0	2	2	2	2	2		15	2	1	0	2	4	4	1	2016-07-29 20:32	2	2	2	2	3	0	2	2	2	2		53.3	2	2	1		0	1	0
1	76	1	1	2	1	0	1	1	2	2	2		16	1	0	1	2	4	4	1	2016-08-23 20:40	2	2	1	2	1	1	2	1	1	2		16.5	2	2	1		1	2	1
1	58	2	1	2	2	0	2	2	2	2	2		34	1	0	0	2	4	4	1	2016-10-17 16:20	2	2	2	2	3	0	2	1	1	2		13.6	2	2	1		1	2	1
1	68	1	2	1	2	0	2	2	2	2	2		21	2	0	0	2	4	4	2		2	2	2	2	3		2	2	2	2		1.6	2	2	1		1	2	1
1	45	1	1	1	1	0	2	2	2	2	2		38	1	0	0	1	1	1	1	2016-12-13 17:00	2	2	1	2	1	1	2	1	1	2		34.9	2	2	2	2	1	2	1
2	36	1	1	1	2	0	2	2	2	2	2		35	2	1	0	2	4	4	1	2016-12-15 19:00	2	2	2	2	3	0	2	1	1	2		24.4	2	2	1		1	2	1
1	49	1	1	1	1	0	2	2	2	2	2		26	1		1	1	1	1	1	2017-01-04 19:00	2	2	1	2	1	0	2	2	2	2		5.8	2	1	1		0	1	0
1	40	1	1	1	1	0	2	2	2	2	2		39	1	1	0	1	1	1	1	2017-01-06 20:40	2	2	1	2	1	1	2	2	2	2		24.4	2	2	1		0	1	0
1	85	2	1	2	2	0	2	1	2	2	2		48	2	0	0	1	1	1	1	2017-02-07 12:00	2	2	2	2	3	0	2	2	2	2			2	2	2	4	1	2	1
1	89	1	1	1	1	0	1	1	2	2	2	1	8	1	0	0	1	1	1	1	2017-03-01 15:10	2	2	2	2	3	1	2	2	2	2		8.1	1	2	1		1	2	1
1	68	1	1	1	1	0	1	2	2	2	2		28	1	0	0	1	1	1	1	2017-04-08 10:20	2	2	1	2	1	1	2	2	2	2		12.6	2	2	1		0	1	0
2	62	2	1	1	2	0	2	2	2	2	2		42	2	0	0	1	1	1	1	2017-04-11 10:35	2	2	2	2	3	0	2	2	2	2		39.7	2	2	1		1	1	1
2	68	1	1	1	2	0	2	2	2	2	2		20	2	1	0	1	1	1	1	2017-05-04 13:30	2	2	2	2	3	0	2	2	2	2		4.1	2	1	2	4	1	2	1
1	55	1	1	1	1	0	2	2	2	2	2		14	1	0	0	1	1	1	1	2017-06-27 21:00	2	2	2	1	2	1	2	2	2	2		6	2	2	1		0	1	0
2	31	1	2	2	2	0	2	2	2	2	2		15	2	0	0	1	1	1	1	2017-07-04 20:05	2	2	2	2	3	0	2	2	2	2		23.9	2	2	2	2	1	2	1
1	37	1	1	1	1	0	2	2	2	2	2		39	2	0	0	1	1	1	1	2017-07-21 17:05	2	2	2	2	3	1	2	2	2	2		100	2	2	2	2	1	2	1
1	33	1	1	2	1	0	2	2	2	2	2		45	1	0	0	1	1	1	1	2017-08-07 21:07	1	2	1	2	1	0	2	2	2	2		10	2	2	1		0	1	0
2	66	2	1	2	2	0	2	1	1	2	1		42	2	0	0	1	1	1	1	2017-08-15 15:40	2	2	2	2	3	0	2	2	2	2		17	2	1	1		1	2	1
1	67	1	1	2	2	0	2	2	2	2	2		31	2		0	1	1	1	1	2017-09-14 16:00	2	2	2	2	3	0	2	2	2	2		5.9	2	2	2	2	1	2	1
2	58	1	1	1	2	0	2	2	2	2	2		26	2	1	0	1	1	1	1	2017-09-16 16:00	2	2	2	2	3	0	2	2	2	2		42.1	2	2	2	2	1	2	1
2	60	2	1	1	2	0	2	2	1	2	2		32	1	0	0	1	1	1	1	2017-12-23 19:20	2	2	2	1	2	1	2	1	1	2		22.1	2	2	1		1	1	1
1	61	2	1	1	1	0	2	2	2	2	2		22	1	0	0	1	1	1	1	2018-01-17 14:20	2	2	2	1	2	0	2	2	2	2		22.3	2	2	1		0	1	0
2	55	1	1	2	2	0	2	2	2	2	2		24	1	0	0	1	1	1	1	2018-02-02 20:15	2	2	2	2	3	0	2	2	2	2		30.8	1	2	1		0	1	0
1	20	1	1	1	1	0	2	2	2	2	2		21	1	0	0	1	1	1	1	2018-02-17 15:20	2	2	1	2	1	0	2	2	2	2		11.3	2	2	1		0	1	0
2	58	2	1	1	1	0	2	2	2	2	2		10	1	0	0	1	1	1	1	2018-02-19 16:50	2	2	1	2	1	1	2	2	2	2		6.1	2	2	1		0	1	0
1	57	2	2	2	2	0	2	2	1	2	2		29	2	0	0	1	1	1	1	2018-03-13 15:30	2	2	2	2	3	0	2	2	2	2		16	2	2	1		1	1	1
1	43	1	2	1	2	0	2	2	2	2	2		49	2	1	0	1	1	1	2		2	2	2	2	3		2	2	2	2		0.1	2	1	2	2	1	2	1
1	52	2	1	1	1	0	2	2	2	2	2		16	1	0	0	1	1	1	1	2018-03-23 13:30	2	2	1	2	1	1	2	1	1	2			2	2	1		0	1	0
1	48	1	2	2	2	0	2	2	2	2	2		26	2	0	0	1	1	1	1	2018-08-21 13:20	2	2	2	2	3		2	1	1	2		20	2	2	2	2	1	2	1
1	26	2	1	1	1	0	2	2	2	2	2		85	1		0	1	1	1	1	2018-09-01 03:00	1	2	2	2	3		2	2	2	2		0.1	2	1	2	3	1	2	1
1	72	1	1	1	2	0	2	1	2	2	2		69	1	1	0	1	1	1	1	2018-10-04 14:10	2	2	2	2	3	0	2	1	1	2		19.4	2	1	2	2	1	2	1
2	48	1	1	1	2	0	2	2	2	2	2		11	2	0	0	1	1	1	1	2018-10-07 13:00	2	2	2	2	3	1	2	1	1	2		16	2	2	1		1	1	1
1	30	1	1	1	1	0	2	2	2	2	2		28	1	1	0	1	1	1	1	2018-10-19 16:05	2	2	1	2	1	1	2	1	1	2		9.2	2	2	1		0	1	0
1	65	1	1	1	1	0	2	2	1	2	2		8	1	0	0	1	1	1	1	2018-11-15 13:10	2	2	2	1	2	1	2	2	2	2		26	2	2	1		0	1	0
1	63	2	1	2	1	0	2	2	2	2	2		40	1	1	0	1	1	1	1	2018-11-19 13:18	2	2	2	1	2	1	2	1	1	2		25.9	2	2	1		1	1	1
1	58	1	1	2	2	0	2	2	1	2	2		40	2	0	0	1	1	1	1	2018-11-27 20:40	2	2	2	2	3	1	2	1	1	2		16	2	2	1		1	1	1
2	54	1	2	1	2	0	2	2	1	2	2		34	2	0	0	1	1	1	1	2018-12-11 17:57	2	2	2	2	3		2	1	1	2		26	2	1	2	3	1	2	1
1	48	1	1	1	1	0	2	2	1	2	2		14	1	0	0	1	1	1	1	2015-11-04 06:40	2	2	2	1	2	1	2	99	2	2		11.3	2	2	1		0	1	0
1	39	1	1	1	2	0	2	2	2	2	2		35	2	1	0	1	1	1	1	2015-11-25 17:40	2	2	2	2	3		2	2	2	2		5.6	2	2	2	4	1	2	1
1	57	2	1	1	1	0	2	2	2	2	2		10	1	0	0	1	2	2	1	2015-11-28 14:20	2	2	2	1	2	1	2	1	1	2		34.2	2	2	1		0	1	0
2	47	1	1	2	2	0	2	2	2	2	2		10	2	0	0	1	1	1	1	2015-10-04 21:00	2	2	2	2	3	0	2	2	2	2		15.2	2	2	2	2	1	2	1
2	41	1	1	2	2	0	2	2	2	2	2		45	1	0	0	1	1	1	1	2015-10-17 19:35	2	2	2	2	3	0	2	2	2	2		7.8	2	2	2	1	1	2	1
1	60	1	1	1	2	0	2	2	2	2	2		27	1	0	0	1	1	1	1	2015-12-12 03:50	2	2	2	2	3		2	2	2	2		26.5	2	2	1		1	1	1
2	19	1	1	2	2	0	2	2	2	2	2		30	2	0	0	1	1	1	1	2015-12-12 19:30	2	2	2	2	3	0	2	1	1	2		20.5	2	2	2	2	1	2	1
1	54	2	1	2	1	0	2	2	2	2	2		24	1	0	0	1	1	1	1	2015-12-20 16:00	2	1	2	1	2	0	2	2	2	2		6.9	2	2	1		0	1	0
1	38	1	1	2	1	0	2	2	2	2	2		23	1	0	0	1	1	1	1	2015-12-27 11:42	2	1	2	2	3	0	1	1	1	2		31.5	2	2	2	3	1	2	1
1	47	2	1	1	2	0	2	1	1	2	2		24	1	0	0	1	1	1	1	2016-01-04 18:40	2	2	2	2	3	0	2	2	2	2		13.4	2	2	1		1	1	1
1	60	1	1	1	1	0	2	2	2	2	2		11	1	0	0	1	1,6,8	1	1	2016-01-19 01:30	2	2	1	2	1	0	2	1	1	2		3.7	2	2	1		0	1	0
1	61	2	1	2	1	0	2	2	2	2	2		73	1	0	0	1	1,6,8	1	1	2016-02-01 16:10	2	2	1	2	1	1	2	1	1	2		2.9	2	2	1		0	1	0
2	56	2	1	2	2	0	2	2	2	2	2		32	2	0	0	1	1,6,8	1	1	2016-02-03 14:30	2	2	2	2	3	1	2	2	2	2		12.2	2	2	2	3	1	2	1
1	63	1	1	2	1	0	2	1	2	2	2		14	1	0	0	1	1,6,8	1	1	2016-02-09 14:00	2	2	2	1	2	1	2	1	1	1		19	2	1	2	1	1	2	1
1	68	2	1	2	1	0	2	2	2	2	2		44	1	0	0	1	1,6,8	1	1	2016-02-26 15:00	2	2	2	2	3	0	1	1	1	2		23	1	2	2	1	1	2	1
1	68	2	1	1	2	0	1	1	1	2	2		58	1	0	0	1	1	1	1	2016-02-15 17:20	2	2	2	2	3	0	2	2	2	2		9.7	2	1	2	1	1	2	1
2	42	1	1	2	2	0	2	2	2	2	2		47	1	1	0	1	1	1	1	2016-03-11 06:05	2	2	2	2	3		2	2	2	2			2	2	2	3	1	2	1
1	31	1	1	1	2	0	2	2	2	2	2		9	1	0	0	1	1,6,8	1	1	2016-03-11 18:40	2	2	1	2	1	0	2	1	1	2		15	2	2	1		0	1	0
1	38	1	1	1	2	0	2	2	2	2	2		54	2	0	0	1	1,6,8	1	1	2016-03-27 17:00	2	2	2	2	3	0	2	1	1	2		17.3	2	2	2	3	1	2	1
2	39	2	1	2	1	0	2	2	2	2	2		30	1	0	0	1	1,6,8	1	1	2016-04-24 01:35	2	2	1	2	1	0	2	2	2	2		11.7	2	2	1		0	1	0
1	35	1	1	2	1	0	2	2	2	2	2		32	1	0	0	1	1	1	1	2016-04-17 15:28	2	2	2	2	3	0	2	1	1	2		12.9	2	2	1		0	1	0
1	68	2	1	1	2	0	2	1	2	2	2		15	1	0	0	1	1	1	1	2016-04-18 15:45	2	2	2	2	3	0	2	1	1	2		6.7	2	2	1		0	1	0
2	43	1	1	2	2	0	2	2	2	2	2		35	2	0	0	1	1	1	1	2016-04-24 05:00	2	2	2	2	3		2	2	2	2		0	2	2	2	2	1	2	1
1	38	1	1	1	1	0	2	2	2	2	2		30	1	0	0	1	1	1	1	2016-04-15 10:00	2	2	2	2	3	0	2	2	2	2		7.6	2	2	1		0	1	0
1	68	2	1	1	2	0	2	2	1	2	2		44	1	0	0	1	1	1	1	2016-06-16 18:40	2	2	2	2	3	0	2	2	2	2		34	2	2	2	2	1	2	1
2	57	1	1	1	2	0	2	2	2	2	2		34	2	0	0	1	1	1	1	2016-06-05 22:00	2	2	2	2	3	0	2	2	2	2		21.6	2	2	2	2	1	2	1
1	52	1	1	2	2	0	2	2	2	2	2		68	2	0	0	1	1	1	1	2016-07-13 19:40	2	2	2	2	3	0	2	2	2	2		14.2	2	2	2	2	1	2	1
2	61	1	1	2	2	0	2	2	2	2	2		45	2	0	0	1	1	1	1	2016-08-01 22:20	2	2	2	2	3	0	2	2	2	2		15.2	2	2	2	2	1	2	1
1	55	2	1	2	1	0	1	1	2	2	2	1	45	1	0	0	1	1,6,8	1	1	2016-09-17 20:00	2	2	1	2	1	1	2	1	1	2		31.2	2	2	1		1	1	1
1	28	2	1	1	2	0	2	2	2	2	2		51	1	1	0	1	1	1	1	2016-09-09 08:50	2	2	2	2	3	0	2	2	2	2		36.4	2	2	2	3	1	2	1
1	56	1	1	1	2	0	1	2	2	2	2		47	2	0	0	1	1	1	1	2016-10-30 05:48	2	2	2	2	3		2	1	1	2		18.6	2	2	2	2	1	2	1
2	74	1	1	1	1	0	2	2	2	2	2		47	1	0	0	1	1	1	1	2016-10-06 20:40	2	2	2	2	3	0	2	2	2	2		38.8	2	2	2	1	1	2	1
1	74	1	1	2	2	0	2	1	2	2	2		15	2	0	0	1	1	1	1	2016-11-22 19:00	2	2	2	2	3	0	2	1	1	2		17.3	2	2	2	3	1	2	1
1	19	1	1	2	2	0	2	2	2	2	2		56	1	0	0	1	1	1	1	2016-11-24 12:30	2	2	2	2	3	0	2	2	2	2		12.4	2	2	1		1	1	1
1	47	1	1	1	2	0	2	1	2	2	2		21	1	0	0	1	1	1	1	2016-11-17 06:50	2	2	2	2	3	0	2	2	2	2		24.2	2	1	2	1	1	2	1
1	59	2	1	1	2	0	2	1	1	2	2		33	1	0	0	1	1	1	1	2016-12-17 06:40	2	2	2	2	3	0	2	2	2	2			2	2	2	3	1	2	1
1	54	1	1	2	2	0	1	2	2	2	2		24	1	1	0	1	1	1	1	2017-01-19 09:50	2	2	1	2	1	0	2	2	2	2		11.1	2	2	1		0	1	0
1	53	1	1	2	2	0	2	2	1	1	1		47	1	0	0	1	1	1	1	2017-01-01 22:30	2	2	2	2	3	0	2	1	1	2		43.4	2	2	1		1	1	1
1	72	2	1	2	2	0	2	2	2	2	2		50	1	1	0	1	1	1	1	2017-01-29 23:35	2	2	2	2	3		2	2	2	2		11.3	2	2	2	3	1	2	1
1	66	2	1	2	1	0	2	1	2	2	2		15	1	0	0	1	1	1	1	2017-01-05 23:30	2	2	1	2	1	0	2	1	1	2		28.2	2	2	1		0	1	0
2	70	2	1	2	1	0	1	1	2	1	2		38	1	0	0	1	1	1	1	2017-02-27 23:40	2	2	2	2	3	0	2	1	1	2		23.2	2	2	1		0	1	0
1	53	1	1	2	2	0	2	2	2	2	2		24	1	0	0	1	1	1	1	2017-02-18 01:40	2	2	2	2	3	0	2	1	1	2		27.1	2	2	1		0	1	0
1	48	1	1	1	1	0	2	2	1	2	2		12	1	0	0	1	1	1	1	2015-11-04 06:40	2	2	2	2	3	0	2	1	1	2		11.3	2	2	1		0	1	0
1	51	1	1	1	1	0	2	1	2	2	2		14	1	0	0	1	1	1	1	2016-01-24 12:00	2	2	2	2	3	0	2	1	1	2		19.6	2	2	1		0	1	0
2	44	2	1	2	1	0	2	2	2	2	2		15	1	0	0	1	1	1	1	2016-03-22 01:15	2	2	1	2	1	0	2	2	2	2		7.2	2	2	1		0	1	0
2	34	1	2	2	1	1	2	2	2	2	2		46	1	0	0	1	1	1	1	2016-08-11 02:00	2	2	2	2	3	1	2	2	2	2		24	1	2	2	1	1	2	1
1	56	2	1	2	1	0	2	2	2	2	2		45	1	0	0	1	1	1	1	2017-06-28 20:00	2	2	1	2	1	0	2	2	2	2		10.3	2	2	1		0	1	0
1	63	2	1	1	1	0	2	2	2	2	2		20	1	0	0	1	1	1	1	2017-07-25 18:00	2	2	1	2	1	0	2	2	2	2		15.2	2	2	1		0	1	0
1	63	1	1	1	1	0	1	2	2	2	2		9	1	0	0	1	1	1	1	2017-07-05 22:30	2	2	1	2	1	0	2	2	2	2		20.6	2	2	1		0	1	0
1	70	2	1	2	2	0	2	2	2	2	2		28	1	0	0	1	1	1	1	2017-07-15 05:40	2	2	2	2	3	1	2	2	2	2		21.9	2	2	2	1	1	1	1
1	39	2	1	1	1	0	2	2	2	2	2		19	1	0	0	1	1	1	1	2017-08-22 03:10	2	2	1	2	1	0	2	2	2	2		11.8	2	2	1		0	1	0
1	61	2	1	1	1	0	2	1	1	2	2		21	1	0	0	1	1	1	1	2017-08-25 23:24	2	2	1	2	1	0	2	2	2	2		10.7	2	2	1		0	1	0
1	22	2	1	1	1	0	2	2	2	2	2		16	1	0	0	1	1	1	1	2017-08-09 17:00	2	2	1	2	1	0	2	2	2	2		17.9	2	2	1		0	1	0
2	31	1	1	1	2	0	2	2	2	2	2		46	2	0	0	1	1	1	1	2017-08-05 20:15	2	2	2	2	3		2	2	2	2			2	1	2	2	1	2	1
1	71	1	1	2	2	0	2	2	2	2	2		48	1	0	0	1	1	1	1	2017-08-19 13:30	2	2	2	2	3	1	2	2	2	2		6.8	2	2	1		1	1	1
2	39	1	2	1	2	0	2	2	2	2	2		51	1	0	0	1	1	1	1	2017-08-11 19:50	2	2	2	2	3	0	2	2	2	2		26.7	2	2	2	2	1	2	1
2	41	1	1	1	2		2	2	2	2	2		34	1	0	0	1	1	1	1	2017-10-07 09:20	2	2	2	2	3		2	2	2	2		48.5	2	2	1		1		
1	60	1	1	1	2	0	2	2	2	2	2		28	1	0	0	1	1	1	1	2017-11-15 21:40	2	2	2	2	3	0	2	2	2	2		6.4	2	2	1		0	1	0
1	59	2	1	1	1	0	2	2	2	2	2		24	1	0	0	1	1	1	1	2017-11-06 18:00	2	2	2	2	3	0	2	2	2	2		21.9	2	1	2	1	1	2	1
1	44	2	1	1	1	0	2	2	2	2	2		20	1	0	0	1	1	1	1	2017-11-21 21:10	2	2	2	2	3	0	2	2	2	2		17.8	2	2	1		0	1	0
1	63	2	1	1	1	0	2	2	2	2	2		9	1	0	0	1	1	1	1	2017-11-26 18:30	2	2	2	1	2	1	2	2	2	2		22.3	2	2	1		0	1	0
1	63	1	1	1	1	0	2	2	2	2	2		14	1	0	0	1	1	1	1	2017-11-16 23:50	2	2	2	2	3	0	2	2	2	2		16.3	2	2	1		0	1	0
1	57	1	1	1	2	0	2	2	2	2	2		30	2	0	0	1	1,8	1	1	2017-12-31 16:15	2	2	2	2	3	0	2	1	1	2		25.2	2	2	2	2	1	2	1

1	74	2	1	1	2	0	2	2	2	2	2		25	1	0	0	1		1	1	1	2017-12-25 15:00	2	2	2	2	3	0	2	2	2	2		16.9	2	2	2	3	1	2	1
1	68	1	1	2	1	0	2	2	2	2	2		44	1	0	0	1		1	1	1	2017-10-10 16:45	2	2	2	2	3	0	2	2	2	2		16.8	2	2	1		1	1	1
1	71	2	1	1	1	0	2	2	2	2	2		25	1	0	0	1	1,6,8		1	1	2017-03-11 15:30	2	2	1	2	1	0	2	1	1	2		17	2	2	1		1	1	1
1	73	2	1	1	1	0	2	2	2	2	2		23	1	0	0	1		1	1	1	2017-04-01 22:30	2	2	1	2	1	0	2	2	2	2		12	2	2	1		0	1	0
2	60	1	1	1	2	0	2	2	2	2	2		48	1	0	0	1		1	1	1	2017-04-10 20:40	2	2	2	2	3	1	2	2	2	2		12.4	1	1	2	1	1	2	1
2	44	1	1	1	2	0	2	2	2	2	2		33	1	0	0	1		1	1	1	2017-04-01 16:00	2	2	1	2	1	0	2	2	2	2		6.7	2	2	1		0	1	0
1	73	1	1	2	2	0	2	2	2	2	2		20	1	0	0	1		1	1	1	2017-05-02 13:50	2	2	2	2	3	0	2	2	2	2		10.4	2	2	1		0	1	0
1	22	1	1	1	2	0	2	2	2	2	2		53	2	0	0	1		1	1	1	2017-05-13 14:30	2	2	2	2	3		2	2	2	2		24.1	2	2	2	4	1	2	1
1	77	1	1	1	2	0	2	2	2	2	2		50	1	1	0	1		1	1	1	2017-05-16 14:10	2	2	2	2	3		2	2	2	2		36.5	2	2	2	2	1	2	1
1	77	1	1	1	2	0	2	2	2	2	2		32	1	0	0	1		1	1	1	2017-06-14 20:00	2	2	2	2	3	0	2	2	2	2		12.9	2	2	1		1	1	1
1	58	1	1	1	2	0	1	2	2	2	2		96	1	1	0	1		1	1	1	2018-12-06 20:17	2	1	1	2	1	0	2	2	2	2		0.4	2	1	2	3	1	2	1
1	57	1	2	2	2	0	2	1	2	2	2		54	1	0	0	1		1		1	2018-06-04 20:40	2	2	2	2	3		2	2	2	2		2.5	2	1	2	1	1	2	1
2	67	2	1	1	2	0	1	2	2	2	2		13	1	0	0	1		1	1	1	2018-11-22 23:30	2	2	2	2	3	0	2	1	1	2		19	2	2	2	3	1	2	1
2	50	1	2	2	1	0	1	2	2	2	2		55	1	1	0	1		1	1	1	2018-01-15 13:00	2	2	1	2	1	0	2	1	1	2		12.2	2	2	1		1	1	1
1	57	2	1	1	2	0	2	2	2	2	2		35	1	0	0	1		1	1	1	2018-11-18 15:55	2	2	2	1	2	1	2	1	1	2		13.4	2	2	1		0	1	0
1	70	2	1	1	1	0	2	1	1	2	2		19	1	0	0	1	2,8		2	1	2018-11-14 18:00	2	2	2	1	2	1	2	1	1	2		11.4	2	2	1		0	1	0
2	47	2	1	1	1	0	2	1	2	2	2		17	1	0	0	1	2,5,6,8		2	1	2018-11-13 18:30	2	2	1	2	1	1	2	1	1	2		14.2	2	2	1		1	1	1
2	45	1	1	2	2	0	2	2	2	2	2		37	1	0	0	1	1,6,8		1	1	2018-09-24 17:15	2	1	1	2	1	0	2	2	2	2		11.3	2	2	1		0	1	0
2	66	1	2	2	2	0	2	1	2	2	2		31	2	0	0	1	1,6,8		1	1	2018-06-26 20:00	2	2	2	2	3		2	2	2	2		18.7	2	2	2	2	1	2	1
1	45	2	1	2	1	1	2	1	2	2	2		28	1	0	0	1		1	1	1	2018-02-09 20:30	2	2	2	1	2	0	2	1	1	2		9.3	2	2	1		0	1	0
2	20	1	1	1	1	0	2	2	2	2	2		16	1	0	0	1		1	1	1	2018-02-16 11:00	2	2	1	2	1	0	2	2	2	2		10.1	2	2	1		0	1	0
1	50	2	1	2	2	0	2	2	2	2	2		27	1	1	0	3		10	1	1	2018-09-08 00:29	1	1	2	1	2		2	2	2	2		25.4	2	1	2	1	1	2	1
2	51	1	1	1	2	0	2	2	1	2	2		41	2	0	0	1		1	1	1	2018-05-08 23:45	2	2	2	2	3		2	2	2	2		34.4	2	2	2	2	1	2	1
1	51	1	1	1	1	0	2	2	1	2	2		12	1	0	0	1		1	1	1	2018-07-25 08:45	2	2	1	2	1	0	2	2	2	2		23.1	2	1	1		0	1	0
2	66	2	2	1	1	0	2	1	2	2	1		28	1	0	0	1		1	1	1	2018-08-06 20:25	2	1	1	2	1	1	2	2	2	2		13.4	1	2	2	1	1	2	1
1	59	2	2	2	1	0	2	1	1	2	2		33	1	0	0	1		1	1	1	2018-08-09 13:40	2	2	1	2	1		2	1	1	2		14.5	1	2	1		0	1	0
1	44	1	1	1	2	0	2	2	2	2	2		35	2	0	0	1		1	1	1	2018-08-25 17:15	2	2	2	2	3		2	2	2	2		14	2	2	2	2	1	2	1
1	63	2	1	2	1	0	2	2	2	2	2		13	1	0	0	1		1	1	1	2018-03-13 17:10	2	2	2	1	2	1	2	1	1	2		31.8	2	1	1		0	1	0
1	58	2	1	1	1	0	2	2	2	2	2		7	1	0	0	1		1	1	1	2018-04-02 15:05	2	2	2	1	2	1	2	1	1	2		10.8	2	2	1		0	1	0
1	64	2	1	2	2	0	1	2	2	2	2		41	1	0	0	1		1	1	1	2018-04-25 07:30	2	2	2	1	2		2	2	2	2		24.8	1	1	2	3	1	2	1
2	63	1	1	1	2	0	2	2	2	2	2		45	2	0	0	1	1,8		1	1	2018-04-26 23:30	2	2	2	2	3		2	1	1	2		19.1	2	2	1		1	2	1
1	43	1	1	2	1	0	1	2	2	2	2		112	1	0	0	1	1,6,8			1	2018-07-01 14:10	2	2	2	2	3		2	2	2	2		7.6	2	2	2	1	1	2	1
2	73	1	1	2	2	0	2	1	1	2	2		26	1		0	1		1	1	1	2018-06-29 09:45	2	2	2	2	3	0	2	2	2	2		17.5	2	1	2	3	1	2	1
1	86	1	1	2	1	0	1	2	2	2	2	1	54	1	1	0	1	2,6,8		2	1	2018-01-25 19:58	2	2	2	2	3		2	2	2	2			2	1	2	1	1	2	1
2	37	1	1	1	1	0	2	2	2	2	2		58	1	0	0	1		1	1	1	2016-01-12 02:42	2	2	2	2	3		2	2	2	2			2	1	2	1	1	2	1
1	57	1	1	1	1	0	2	1	2	2	2		14	1	0	0	1		1	1	1	2016-03-19 00:00	2	2	1	2	1	1	2	2	2	2		93.5	2	2	1		0	1	0
1	58	2	1	1	1	0	2	2	2	2	2		25	1	0	0	1		1	1	1	2016-04-15 18:43	2	2	1	2	1		2	1	1	2			2	2	1		0	1	0
1	76	2	1	2	2	0	2	2	1	2	2		9	2	0	0	1	1,8		1	1	2016-04-25 14:45	2	1	2	2	3	1	2	1	1	1		100	1	2	2	4	1	2	1
1	52	2	2	1	1		2	2	2	2	2		7	1	0	0	1		1	1	1	2016-01-22 02:22	2	2	2	2	3	0	2	2	2	2		100	2	2	1		1		
2	76	1	2	1	2	1	2	1	2	2	2		37	1	0	0	1		1	1	1	2016-07-30 03:20	2	2	2	2	3		2	2	2	2		100	2	1	2	1	1	2	1
1	78	1	2	2	1		2	2	1	2	2		60	1	0	0	1	1,8		1	1	2016-07-31 13:10	2	2	1	2	1		2	2	2	2		0.3	2	2	1		1		
1	75	1	1	1	2	0	2	1	1	1	2		28	2	0	0	1	1,8		1	1	2016-05-04 14:30	2	2	2	2	3	0	2	1	1	2		100	2	2	2	1	1	2	1
1	42	2	1	1	2	0	2	1	2	2	2		20	1	0	1	3		9	9	1	2016-06-09 14:25	1	2	2	1	2	1	2	2	2	2		2	1	2	1		0	1	0
1	61	2	1	2	1	1	1	2	2	2	2		29	1	0	0	1		1	1	1	2016-08-26 09:20	2	2	2	1	2	0	2	1	2	1		70.8	2	2	2	1	1	2	1
1	58	2	2	1	2	0	2	2	2	2	2		33	1	0	0	1		2	2	1	2016-10-09 17:09	2	2	2	2	3		2	2	2	2			2	1	2	1	1	2	1
2	58	1	2	1	2		2	2	2	2	2		32	2	1	0	1	1,2,8		1	1	2016-10-01 01:50	2	2	2	2	3		2	1	1	2		96.6	2	2	1		1		
1	51	2	1	1	2		2	2	2	2	2		11	2	0	0	1		1	1	1	2016-10-15 11:52	2	2	2	2	3		2	1	1	2		86.3	2	2	1		0		
1	75	2	1	1	1	1	2	1	1	2	2		35	1	0	1	2		4		1	2016-10-20 01:35	1	2	2	1	2	1	2	1	2	1		1	1	1	2	1	1	2	1
2	53	1	1	1	1	0	2	1	2	2	2		70	1		1	1		2	2	1	2015-10-01 07:00	2	2	1	2	1	0	2	1	1	2		15.8	2	1	1		0	1	0
2	19	1	1	1	2	0	2	2	2	2	2		20	1		0	1		1	1	1	2015-10-11 16:00	2	2	2	2	3	0	2	2	2	2		9.6	2	1	1		0	1	0
2	38	1	2	2	1	0	2	2	2	2	2		34	1	0	0	1		1	1	1	2015-10-19 13:40	2	2	2	1	2	1	2	1	1	2		6.3	2	2	1		0	1	0
1	60	2	2	1	2	0	1	2	2	2	2		22	1	0	0	1		1	1	1	2015-11-12 00:00	2	2	2	2	3		2	1	1	2		34.8	2	1	1		0	1	0
2	65	1	1	1	2	0	2	1	2	2	2		30	1		0	1		1	1	1	2015-11-16 19:10	2	2	2	2	3	0	2	1	1	2		27.2	2	2	1		1	1	1
2	26	2	1	2	2	0	2	2	2	2	2		5	1	0	0	1		1	1	1	2015-12-15 02:50	2	1	2	2	3		2	1	1	2		45.4	2	2	1		1	1	1
1	63	2	1	2	2	0	2	1	2	2	1		33	1	0	0	1		1	1	1	2015-12-16 13:00	2	2	2	2	3		2	1	2	1		28.1	1	1	2	3	1	2	1
1	61	2	1	1	2	0	2	2	2	1	2		15	1	1	0	1		1	1	1	2015-12-31 17:10	2	2	2	1	2	1	2	1	1	2		10.7	2	2	1		0	1	0
1	59	2	2	2	1	0	1	2	2	2	2		13	1	0	0	1		1	1	1	2016-01-11 13:20	2	2	1	2	1	0	2	1	1	2		8.6	2	1	1		0	1	0
2	61	2	1	2	2	0	2	2	2	2	2		15	1	0	0	1		1	1	1	2016-01-15 00:10	2	2	2	2	3		2	2	2	2		9.2	2	2	1		1	2	1
2	74	1	2	1	2	0	2	1	1	2	2		60	1	0	0	1		1	1	1	2016-01-02 18:30	2	2	1	2	1		2	1	1	2		18	1	1	2	1	1	2	1
2	74	1	2	2	2	0	2	1	2	2	2		70	2	0	0	1		1	1	1	2016-02-01 00:30	2	2	2	2	3		2	1	2	1		28.3	1	1	2	3	1	2	1
1	63	2	1	1	1	0	2	2	2	2	2		35	1	0	0	1		1	1	1	2015-11-11 14:15	1	2	2	1	2		2	2	2	2		0	2	1	2	1	1	2	1
1	43	2	2	2	2	0	2	2	2	2	2		41	2	0	0	1		1	1	1	2015-11-14 02:00	2	2	2	2	3		2	1	1	1		31	2	1	2	3	1	2	1
1	70	1	2	2	2	0	2	2	1	2	2		90	1	0	0	1		1	1	1	2016-02-11 15:40	2	2	2	2	3	0	2	2	2	2		10.7	2	2	1		0	1	0
2	42	2	1	1	2	0	2	2	2	2	2		30	2	0	0	1		1	1	1	2016-02-13 14:40	2	2	2	2	3		1	2	2	2		1	1	1	2	1	1	2	1
1	48	1	2	1	2	0	2	2	2	2	2		36	2	1	0	1		1	1	1	2016-02-14 18:35	2	2	2	2	3		2	1	1	2		10.3	2	2	1		1	1	1
2	43	2	1	1	2	0	2	2	2	2	2		39	1	1	0	1		1	1	1	2016-02-16 14:30	2	2	1	2	1	0	2	1	1	2		8.4	2	2	1		0	1	0
1	67	2	2	2	2	0	2	2	2	2	2		7	2	0	1	1		2	2	1	2016-02-17 04:50	2	2	2	2	3		2	1	1	2		13.5	2	2	1		0	1	0
1	54	2	1	1	2	0	2	2	1	2	2		30	2	0	0	1		1	1	1	2016-02-21 05:30	2	2	2	2	3		1	1	1	2		18.3	2	2	1		1	1	1
1	58	2	1	1	2	0	2	2	1	2	2		20	1	0	0	1		1	1	1	2016-02-28 14:35	2	2	2	2	3	0	2	1	2	1		34.4	1	1	2	1	1	2	1
1	78	2	1	2	2	0	1	1	1	2	1		25	1	0	0	1		1	1	1	2016-03-03 00:20	2	2	2	1	2		2	1	1	2		9.5	1	1	2	3	1	2	1
1	35	1	1	2	2	0	1	2	2	1	2		27	1	0	0	1		1	1	1	2016-03-18 20:25	2	2	1	2	1	0	2	1	1	2		13.4	2	2	1		0	1	0
1	70	1	1	1	1	0	2	1	2	2	2		24	1	0	0	1		1	1	1	2016-03-23 07:05	2	2	2	1	2	1	1	1	2	1		23.4	2	1	2	3	1	2	1
1	73	1	2	2	2	0	2	2	1	1	2		47	2	0	0	1		1	1	1	2016-03-23 22:35	2	2	2	2	3		2	1	2	1		35	2	1	2	3	1	2	1
2	68	1	2	1	1	0	2	2	2	1	2		30	1	0	0	1		1	1	1	2016-03-13 01:00	2	2	2	1	2	1	2	1	1	2		10.4	2	1	1		0	1	0
1	90	1	2	1	2	0	2	1	2	2	2		38	1	0	0	1		1	1	1	2016-03-26 16:40	2	2	2	1	2		2	1	2	1		17.8	2	1	2	1	1	2	1
2	81	2	1	1	1	0	2	1	1	2	2		18	1	0	0	1		1	1	1	2016-04-04 15:50	2	2	1	2	1	0	2	1	1	2		17.6	2	2	1		0	1	0
1	55	2	1	1	2	0	2	1	2	2	1		10	1	0	0	1		1	1	1	2016-04-10 03:40	2	2	2	2	3	0	2	1	1	2		15.5	1	2	1		0	1	0
1	70	1	2	1	2	0	1	2	2	2	2		38	1	0	0	1		1	1	1	2016-04-10 17:25	2	2	2	2	3	0	2	1	1	2		13.8	2	1	2	1	1	2	1
1	59	2	1	1	2	0	2	1	2	2	2		43	1	0	0	1		1	1	1	2016-04-14 14:10	2	2	2	2	3	0	2	1	2	1		17.2	2	1	2	1	1	2	1
1	49	1	2	2	2	0	2	1	1	2	2		45	1	1	0	1		2	2	1	2016-05-04 17:15	2	2	2	2	3		2	2	2	2		9.9	2	2	2	3	1	2	1
2	51	2	2	1	2	0	2	2	2	2	2		19	2	0	0	1		1	1	1	2016-05-05 17:05	2	2	2	2	3		1	1	2	1		16.9	2	2	2	1	1	2	1
2	57	1	2	1	1	0	2	1	2	2	2		54	1	1	0	1		1	1	1	2016-05-07 14:30	2	2	1	2	1	0	2	1	1	2		14.7	2	2	1		1	2	1
1	56	1	2	1	1	0	2	1	1	2	2		16	1	0	0	1		1	1	1	2016-05-13 12:30	2	2	2	1	2	0	2	1	1	2		13.7	2	2	1		0	1	0
1	62	1	1	1	1	0	2	1	2	2	2		15	1	1	0	1		1	1	1	2016-05-14 00:48	2	2	2	1	2	1	2	1	1	2		18.8	2	1	1		0	1	0
1	45	2	1	1	2	1	2	2	2	2	2		5	1	0	0	1		1	1	1	2016-05-16 14:14	2	2	1	2	1	0	2	2	2	2		6.1	2	2	1		0	1	0
2	74	2	2	2	2	0	2	1	1	2	2		32	2	0	1	1		2	2	1	2016-05-24 21:00	2	2	2	2	3	0	2	1	2	1		20.2	2	1	2	3	1	2	1
1	50	2	2	2	2	0	2	2	1	1	2		10	2	0	0	1		1	1	1	2016-06-05 22:10	2	2	2	2	3	0	2	1	2	1		17.9	1	1	2	3	1	2	1
1	46	2	1	2	2	0	2	2	2	2	2		20	1	0	0	1		1	1	1	2016-06-10 00:20	2	2	2	1	2	1	2	2	2	2		8.2	2	2	1		0	1	0
1	69	1	2	1	1	0	1	1	2	2	2	1	53	1	0	0	1		1	1	1	2016-06-12 23:20	2	2	1	2	1	1	2	2	2	2		8.8	2	2	1		1	2	1
2	83	1	2	2	2	0	1	1	2	2	1		10	2	0	0	1		1	1	1	2016-06-24 06:35	2	2	2	2	3	0	2	2	2	2		8.4	1	2	1		0	1	0
1	61	1	2	1	1	0	2	2	2	2	2		21	1	1	0	1		1	1	1	2016-07-04 22:00	2	2	1	2	1		2	1	2	1		15.7	2	1	1		1	2	1
1	86	2	2	2	2	0	2	2	2	2	2		37	2	1	0	1		1	1	1	2016-07-08 01:35	2	2	2	2	3		2	2	2	2		14.6	2	2	2	3	1	2	1
2	68	1	1	2	2	0	1	1	1	1	1		16	1	0	0	1		1	1	1	2016-07-13 08:30	2	2	2	2	3	1	2	2	2	2		10.2	1	2	1		0	1	0
2	76	2	1	2	1	0	1	1	2	2	2		18	1	1	0	1		1	1	1	2016-07-17 15:50	2	2	2	1	2	1	2	1	1	2		9	2	2	1		0	1	0
1	43	1	1	1	1	1	2	1	2	2	2		15	1	0	0	1		1	1	1	2016-07-17 22:50	2	2	2	1	2	0	2	1	1	2		11.4	2	2	1		0	1	0
2	65	2	2	2	2	0	2	1	2	2	2		75	2	0	0	1		1	1	1	2016-07-18 12:45	2	2	2	2	3		2	1	1	2		14.6	2	2	2	1	1	2	1
2	66	1	1	2	2	0	2	2	1	2	1		45	1	0	0	1		1	1	1	2016-08-08 04:30	2	2	2	2	3	0	2	2	2	2		5.7	1	1	2	1	1	2	1
1	69	2	1	1	1	0	1	1	1	1	2	1	40	1	0	0	1		1	1	1	2016-08-08 15:30	2	2	2	1	2	1	2	1	1	2		15.8	2	1	1		1	2	1
1	47	2	1	1	2	0	2	1	1	2	1		8	1	0	0	1		1	1	1	2016-08-12 15:50	2	2	2	2	3	0	2	1	1	2		15.6	1	2	1		0	1	0
1	26	1	2	1	2	0	2	2	1	2	2		25	2	0	0	1		2	2	1	2016-08-19 00:00	2	2	2	2	3		2	1	2	1		37	2	1	2	1	1	2	1
1	34	1	1	1	1	0	2	2	2	2	2		30	1	1	0	1		1	1	1	2016-08-19 14:35	2	2	1	2	1	0	2	2	2	2		7.5	2	2	1		0	1	0
1	49	2	1	1	2	0	2	2	2	2	2		18	1	0	0	1		1	1	1	2016-08-21 16:45	2	2	2	1	2	0	2	1	1	2		10.6	2	2	1		1	2	1
1	61	1	1	1	2	0	1	1	1	1	2		40	1	0	0	1		1	1	1	2016-09-14 00:15	2	2	1	2	1		2	1	1	2		14.1	2	2	1		1	2	1
1	50	1	1	2	2	0	2	2	1	2	2		113	1	0	0	1		1	1	1	2016-09-21 07:05	2	2	2	1	2	1	2	1	1	2		19.8	1	2	1		0	1	0
1	65	2	1	1	2	0	2	2	2	2	2		22	1	0	0	1		1	1	1	2016-09-29 21:20	2	2	1	2	1	0	2	2	2	2		6.8	2	2	1		0	1	0
2	67	1	1	2	2	0	2	1	2	2	2		40	1	0	0	1		2	2	1	2016-10-03 13:40	2	2	1	2	1		2	1	1	2		8.9	2	2	2	3	1	2	1
1	47	2	1	1	1	0	2	2	2	2	1		25	1	0	0	1		1	1	1	2016-10-10 22:00	2	2	1	2	1	0	2	1	2	1		18.1	2	2	1		0	1	0
1	65	1	1	2	2	0	2	2	2	2	2		26	1	0	0	1		1	1	1	2016-10-16 17:40	2	2	2	2	3	0	2	1	1	2		8.6	2	2	1		1	2	1
1	68	1	1	2	2	0	2	2	2	2	2		35	2	0	0	1		1	1	1	2016-10-04 18:10	2	2	2	2	3		2	1	2	1		18.7	2	2	2	3	1	2	1
1	62	1	1	2	1	0	2	2	2	2	2		10	1	0	0	1		1	1	1	2016-10-18 01:35	2	2	1	2	1	1	2	1	1	2		16.5	2	2	1		0	1	0
1	84	2	1	2	2	0	1	1	1	2	1	1	52	2	0	0	1		1	1	1	2016-10-19 17:15	2	2	2	2	3		2	1	2	1		17.3	1	2	2	3	1	2	1
1	70	2	1	2	2	0	2	2	1	2	2		46	1	1	0	1		1	1	1	2016-10-26 18:00	2	2	1	2	1		2	1	2	1		18.3	2	2	2	3	1	2	1
2	70	2	2	2	2	0	1	1	1	1	1	1	5	1	0	0	1		1	1	1	2016-11-08 10:40	2	2	2	1	2		2	1	2	1		19.1	1	2	1		1	2	1
1	70	1	2	2	2	0	2	1	2	2	2		39	2	1	1	1		1	1	1	2016-11-09 23:50	2	2	2	2	3	0	2	1	1	2		21.8	2	2	1		1	2	1
1	75	2	2	2	2	0	2	2	2	2	2		75	1	1	0	1		1	1	1	2016-11-24 16:30	2	2	2	1	2		2	2	2	2		0	2	2	2	1	1	2	1

1	57	2	1	2	1	0	1	1	2	2	2		45	1	1	0	1	1	1	1	2016-11-29 21:20	2	2	1	2	1		2	1	1	2		32	2	1	2	1	1	2	1
1	75	2	2	1	2	0	2	1	1	2	2		45	2	1	0	1	1	1	1	2016-12-03 15:10	2	2	2	2	3	0	2	1	2	1		13.5	1	1	2	1	1	2	1
1	52	2	1	2	1	0	2	2	2	2	2		7	1	1	0	1	1	1	1	2017-01-11 20:30	2	2	1	2	1		2	1	1	2		13.8	2	2	1		0	1	0
2	76	1	1	1	2	0	2	1	2	1	2		15	2	0	0	1	1	1	1	2017-01-26 00:18	2	2	2	2	3		1	1	2	1		27.8	1	1	2	3	1	2	1
1	59	2	1	2	2	0	2	2	2	2	2		20	1	0	0	1	1	1	1	2017-02-04 23:15	2	2	1	2	1	0	2	1	1	2		12.7	2	2	1		0	1	0
1	83	2	1	2	2	0	2	2	1	2	2		45	2	0	0	1	1	1	1	2017-02-06 04:00	2	2	2	2	3		2	1	2	1		21.1	2	1	2	3	1	2	1
1	56	2	2	1	2	0	2	2	2	2	2		48	2	0	0	1	1	1	1	2017-02-15 17:05	2	2	2	2	3		2	1	2	1		16.8	2	2	1		1	2	1
1	87	2	1	1	2	0	2	1	2	2	2		12	2	1	0	1	2	2	1	2017-02-19 20:30	2	2	2	2	3		1	1	2	1		17.1	2	2	1		1	2	1
1	55	2	2	1	2	0	2	1	2	2	2		32	2	0	0	1	1	1	1	2017-03-02 16:30	2	2	2	2	3		2	1	2	1		25.7	1	2	2	3	1	2	1
1	52	2	2	1	1	0	2	2	2	2	2		54	1	0	0	1	1	1	1	2017-03-13 11:00	2	2	1	2	1		2	1	2	1		34.2	2	1	1		1	2	1
1	50	1	2	1	2	0	1	2	1	2	2	1	97	1	1	1	1	2,9	2,9	1	2017-03-15 17:00	1	2	2	1	2	1	2	1	2	1		18.4	1	2	2	1	1	2	1
1	58	1	2	2	2	0	2	1	1	2	1		81	2	0	0	1	1	1	1	2017-03-17 03:20	2	2	1	2	1	0	2	1	1	2		10.2	1	2	1		1	2	1
1	73	1	1	1	2	0	2	1	1	2	1		43	2	0	0	1	1	1	1	2017-03-17 23:30	2	2	2	2	3		2	1	1	1		36	1	2	2	3	1	2	1
2	71	2	2	2	2	0	2	1	2	2	2		30	1	0	0	1	1	1	1	2017-04-17 14:00	2	2	1	2	1		2	1	1	2		7.8	2	2	1		1	2	1
2	51	2	1	1	1	0	2	1	2	2	2		66	1	1	0	1	1	1	1	2017-04-15 22:30	2	2	2	2	3	0	2	2	2	2		2.3	2	2	2	1	1	2	1
1	78	1	2	2	2	0	2	1	1	2	2		46	1	1	0	1	1	1	1	2017-04-21 17:20	2	2	2	2	3		2	1	2	1		17.7	1	1	2	3	1	2	1
1	44	2	1	1	1	1	2	2	2	2	2		15	1	0	0	1	1	1	1	2017-04-23 03:15	2	2	1	2	1	0	2	1	1	2		6.9	2	2	1		0	1	0
2	33	2	1	1	2	1	2	2	1	2	2		14	1	1	0	1	1	1	1	2017-04-30 18:40	2	2	2	2	3	0	2	1	2	1		32.2	2	2	2	3	1	2	1
1	80	1	2	2	2	0	2	1	1	2	2		22	2	0	0	1	2	2	1	2017-05-08 03:00	2	2	2	2	3		2	1	2	1		17	2	1	1		1	1	1
2	76	2	1	1	2	0	2	2	2	2	2		23	1	0	0	1	1	1	1	2017-05-20 13:50	2	2	2	2	3	0	2	2	2	2		0.6	1	2	2	1	1	2	1
1	30	2	2	2	2	0	2	2	2	2	2		50	2	1	0	1	1	1	1	2017-06-03 19:10	2	2	2	2	3		2	1	1	2		16.9	2	2	1		1	2	1
1	75	2	1	2	2	0	2	1	1	2	2		20	2	1	0	1	1	1	1	2017-06-07 21:40	2	2	2	2	3	0	2	1	2	1		18.3	1	2	2	3	1	2	1
1	52	2	1	1	1	0	2	2	2	2	2		15	1	0	0	1	1	1	1	2017-06-20 10:40	2	2	2	1	2	0	2	1	1	2		8.9	2	2	1		0	1	0
2	63	1	2	1	2	0	2	2	1	1	1		61	1	0	0	1	1	1	1	2017-06-24 21:50	2	2	2	2	3	0	2	1	2	1		29.7	1	2	2	3	1	2	1
1	57	1	2	1	1	0	1	1	2	1	2		41	1	0	0	1	1	1	1	2017-06-26 17:45	2	2	2	1	2	1	2	1	1	2		14.6	2	2	1		0	1	0
1	62	2	1	1	2	0	1	2	2	2	2		37	1	0	0	1	1	1	1	2017-06-30 18:20	2	2	2	2	3	0	2	1	2	1		4.8	1	2	2	1	1	2	1
1	70	2	1	2	1	0	2	2	2	2	2		17	1	0	0	1	2	2	1	2017-07-01 17:30	2	2	2	2	3	0	2	1	1	2		18.2	2	2	1		0	1	0
1	48	1	1	1	2	0	2	1	2	1	2		52	2	1	0	1	1	1	1	2017-07-04 12:15	2	2	2	2	3		2	1	2	1		17.4	2	2	1		1	2	1
1	73	1	1	1	2	0	2	2	2	2	2		40	1	0	0	1	1	1	1	2017-07-18 17:00	2	2	1	2	1	0	2	1	2	1		12.5	2	2	1		1	1	1
1	42	2	1	2	2	0	2	2	2	2	2		40	1	1	0	1	1	1	1	2017-07-19 20:30	1	2	2	1	2		1	1	2	1		40	1	1	2	3	1	2	1
1	38	1	2	2	2	0	2	2	2	2	2		42	2	0	0	1	1	1	1	2017-07-25 20:10	2	2	2	2	3	0	2	1	1	1		26.1	2	2	1		1	2	1
2	58	1	2	1	2	0	2	2	1	2	2		30	2	1	0	1	1	1	1	2017-07-31 13:40	2	2	2	2	3		2	1	1	1		24.2	2	2	1		1	2	1
1	35	2	1	1	1	0	2	2	2	2	2		18	1	0	0	1	1	1	1	2017-08-17 14:48	2	2	1	2	1	0	2	1	1	2		8.2	2	2	1		0	1	0
2	39	2	1	1	2	0	2	2	2	2	2		14	2	0	0	1	1	1	1	2017-08-23 17:35	2	2	2	2	3		2	1	1	2		4.6	2	2	1		0	1	0
1	64	2	2	2	2	1	2	1	1	2	2		70	1	0	0	1	1	1	1	2017-08-24 10:40	2	2	1	2	1		2	1	1	1		39.7	1	2	2	3	1	2	1
1	64	1	2	2	2	0	2	1	1	2	2		43	1	0	0	1	1	1	1	2017-08-31 03:40	2	2	2	2	3	0	2	1	2	1		17.8	2	2	1		1	2	1
1	68	2	2	2	2	0	2	1	1	2	2		60	2	1	0	1	1	1	1	2017-09-11 16:40	2	2	2	2	3		2	1	2	1		18.4	2	2	1		1	1	1
2	75	1	1	1	2	1	2	1	2	2	2		42	2	0	0	1	1	1	1	2017-09-14 01:45	2	2	2	2	3		2	1	2	2		12	2	2	1		1	1	1
2	71	1	2	2	2	0	2	1	1	1	2		47	2	0	0	1	2	2	1	2017-09-16 19:00	2	2	2	2	3		2	1	2	1		13.9	2	2	2	3	1	2	1
1	54	1	1	1	1	0	2	2	2	2	2		62	1	0	0	1	1	1	1	2017-09-17 10:00	2	2	2	2	3	0	2	1	2	1		28.6	2	2	1		1	2	1
2	45	1	2	2	2	1	2	1	2	2	2		41	2	0	0	1	1	1	1	2017-09-30 02:30	2	2	2	2	3		2	1	1	2		5.2	2	2	2	3	1	2	1
2	47	2	1	2	2	0	2	2	2	2	2		60	1	0	0	1	2	2	1	2017-10-06 02:00	2	2	2	2	3		2	1	1	2		18.2	1	2	1		1	2	1
1	48	2	2	1	1	0	1	2	2	2	2	1	20	1	0	0	1	1	1	1	2017-10-06 21:00	1	2	1	2	1	1	2	1	1	1		20.3	2	1	1		0	1	0
2	65	1	1	2	1	0	2	1	2	2	1		54	1	0	0	1	1	1	1	2017-10-09 09:10	2	2	2	2	3	0	2	1	1	2		8.7	2	2	1		1	2	1
1	45	1	2	1	1	1	2	1	1	2	2		76	1	1	0	1	1	1	1	2017-10-09 19:00	2	2	2	1	2	1	2	1	2	1		12.2	2	2	2	3	1	2	1
2	58	1	1	1	2	0	2	1	2	2	2		39	2	0	0	1	1	1	1	2017-10-20 23:20	2	2	1	2	1	1	2	1	2	1		16.8	2	1	1		1	2	1
2	62	1	2	2	2	0	2	1	2	2	2		25	2	0	0	1	1	1	1	2017-10-25 16:15	2	2	2	2	3		2	1	1	1		40.5	1	2	2	3	1	2	1
1	55	1	1	1	1	0	2	1	2	2	2		19	1	0	0	1	1	1	1	2017-11-05 15:20	1	2	2	1	2	1	2	1	2	1		22.7	1	2	1		0	1	0
1	64	2	1	1	2	0	2	1	1	2	1		12	1	0	0	1	1	1	1	2017-11-06 11:30	2	2	2	2	3	0	2	2	2	2		13.2	1	2	1		1	2	1
1	60	1	1	1	2	0	2	2	2	2	2		32	2	0	0	1	1	1	1	2017-11-08 16:10	2	2	2	2	3	0	2	2	2	2		11.5	2	2	1		1	2	1
1	59	2	1	1	2	0	1	1	1	2	2	1	19	1	0	0	1	1	1	1	2017-11-10 12:00	2	2	1	2	1	1	2	1	1	2		11.5	2	1	1		1	1	1
1	53	2	1	1	1	0	2	2	2	2	2		17	1	1	0	1	1	1	1	2017-11-12 14:45	2	2	2	1	2		2	1	1	2		6.5	2	1	2	1	1	2	1
2	76	1	2	2	2	0	2	1	1	2	2		60	1	1	0	1	1	1	1	2017-11-15 22:40	2	2	2	2	3	1	2	1	1	2		7.1	2	2	2	1	1	2	1
1	67	1	1	2	2	0	2	1	1	2	2		25	2	0	0	1	1	1	1	2017-11-16 00:50	2	2	2	2	3		2	2	2	2		14.9	2	2	1		1	2	1
2	52	1	2	1	2	0	2	2	2	2	2		40	2	0	0	1	1	1	1	2017-11-20 21:45	2	2	2	2	3		2	1	1	2		18.6	2	2	1		1	2	1
1	58	2	2	1	2	0	2	2	2	2	2		45	2	0	0	1	1	1	1	2017-11-28 00:45	2	2	2	2	3		2	1	2	1		29	2	2	1		1	2	1
1	64	1	2	2	2	0	2	1	2	2	1		68	2	1	0	1	1	1	1	2017-12-05 22:45	2	2	2	2	3		2	1	2	1		11.3	1	2	2	1	1	2	1
1	55	2	2	2	2	0	2	2	2	2	2		25	2	0	0	1	1	1	1	2017-12-12 22:30	2	2	1	2	1		2	2	2	2		15.9	1	2	1		1	1	1
1	57	2	1	1	1	0	1	1	1	1	2	1	26	1	0	0	1	1	1	1	2017-12-17 14:20	2	2	1	2	1	0	2	1	1	2		13.4	2	2	1		0	1	0
1	62	2	1	1	2	0	2	2	2	2	2		71	2	1	0	1	1	1	1	2017-12-18 13:00	2	2	2	2	3		2	1	2	1		27.7	2	2	2	3	1	2	1
2	25	1	1	1	2	0	2	2	2	2	2		52	2	1	0	1	1	1	1	2017-12-30 22:05	2	2	2	2	3		2	2	2	2		6	2	2	1		1	2	1
1	68	1	1	1	1	0	2	1	2	2	2		54	1	0	0	1	1	1	1	2017-12-31 12:15	2	2	2	1	2	1	2	2	2	2		10.3	2	2	1		0	1	0
1	30	1	1	1	1	0	2	2	2	2	2		25	1	0	0	1	1	1	1	2018-01-04 13:00	2	2	1	2	1	0	2	1	1	2		15.6	2	2	1		0	1	0
1	81	1	1	2	2	0	1	1	2	2	2	1	49	1	0	0	1	1	1	1	2018-01-05 13:25	2	2	2	1	2		2	1	2	1		21.6	2	2	2	3	1	2	1
1	47	1	1	1	2	0	2	2	1	2	2		33	2	0	0	1	2	2	1	2018-01-30 21:00	2	2	2	2	3	0	2	1	2	1		16.3	2	2	1		1	2	1
1	76	2	1	1	2	0	2	1	1	2	2		11	2	1	0	1	1	1	1	2018-02-01 08:45	2	2	2	2	3	0	2	1	1	2		7.3	2	2	1		1	1	1
1	79	1	1	2	1	0	2	1	2	2	2		24	1	0	0	1	1	1	1	2018-02-04 04:10	2	2	1	2	1	0	2	2	2	2		12.3	2	2	1		0	1	0
1	51	1	2	1	1	0	2	2	2	2	2		14	1	1	0	1	1	1	1	2018-02-26 08:00	2	2	1	2	1	0	2	1	1	2		10.6	2	2	1		0	1	0
2	69	2	1	1	1	0	2	2	2	2	2		21	1	1	0	1	1	1	1	2018-03-13 20:35	2	2	2	1	2		2	1	1	2		11.7	2	2	1		0	1	0
2	67	1	2	2	1	0	2	2	2	2	2		16	1	0	0	1	1	1	1	2018-03-16 00:10	2	2	1	2	1	1	2	2	2	2		9.4	2	2	1		0	1	0
1	64	2	1	2	1	0	1	1	1	2	1	1	12	1	0	0	1	1	1	1	2018-03-18 02:00	2	2	2	1	2	1	2	2	2	2		17.7	1	2	2	3	1	2	1
1	54	2	1	1	1	0	2	2	2	2	2		16	1	0	0	1	1	1	1	2018-03-24 18:00	2	2	2	1	2		2	1	1	2		9.6	2	2	1		0	1	0
1	60	1	2	1	2	1	2	1	1	2	2		34	1	0	0	1	1	1	1	2018-03-25 14:00	2	2	1	2	1		2	1	1	2		8.4	2	2	1		1	2	1
1	72	1	1	2	2	0	2	1	2	1	1		37	2	0	0	1	1	1	1	2018-04-02 19:30	2	2	1	2	1		2	1	2	1		23.5	2	2	1		1	2	1
1	52	1	2	1	1	0	1	2	2	2	2		34	1	0	1	1	1	1	1	2018-04-12 16:30	2	2	1	2	1	0	2	2	2	2		25.9	2	2	1		0	1	0
1	75	2	1	1	2	0	2	1	1	2	2		6	1	1	0	1	1	1	1	2018-04-14 01:30	2	2	1	2	1	0	2	1	2	1		20.6	1	2	1		1	1	1
1	37	2	2	1	2	0	2	2	2	2	2		36	2	0	0	1	1	1	1	2018-04-14 17:00	2	2	2	2	3		2	2	2	2		3	2	2	1	4	1	2	1
1	45	2	2	1	1	0	2	1	2	2	2		36	1	0	0	1	1	1	1	2018-04-25 02:10	2	2	1	2	1	1	2	1	1	2		11.2	2	2	1		0	1	0
1	39	2	2	2	2	0	2	2	2	2	2		22	2	1	0	1	1	1	1	2018-04-28 10:50	2	2	2	2	3		2	2	2	2		13.3	2	2	1	4	1	2	1
1	54	2	1	2	2	0	2	2	2	2	2		48	1	0	0	1	1	1	1	2018-05-29 10:20	2	2	1	2	1		2	2	2	2		15	1	2	1		1	1	1
1	43	2	1	1	2	0	2	1	2	2	2		20	1	0	0	1	1	1	1	2018-06-09 15:45	2	2	1	2	1		2	1	1	2		7.1	2	2	1		0	1	0
1	49	1	2	1	2	0	2	2	1	2	2		60	2	1	0	1	1	1	1	2018-06-13 09:00	2	2	2	2	3		2	1	2	1		46.4	1	2	1		1	2	1
2	33	1	2	1	1	0	2	2	2	2	2		33	1	0	1	1	1	1	1	2018-06-17 01:00	2	2	2	2	3		2	2	2	2		5.1	1	2	1		1	1	1
1	42	1	2	2	2	0	2	2	2	2	2		52	2	0	0	1	1	1	1	2018-06-21 17:00	2	2	2	2	3		2	2	2	2		20.8	2	2	2	3	1	2	1
1	52	1	1	1	1	0	2	2	2	2	2		22	1	0	0	1	1	1	1	2018-06-27 17:45	2	2	1	2	1		2	1	1	2		16.5	2	2	1		0	1	0
2	70	2	1	1	2	0	2	2	1	2	2		6	1	1	0	1	1	1	1	2018-07-04 16:40	2	2	1	2	1		2	2	2	2		21.7	2	1	1		1	2	1
1	18	2	1	1	2	0	2	2	2	2	2		43	1	1	0	1	1	1	1	2018-07-09 17:30	2	2	2	2	3	0	2	2	2	2		9.8	2	2	1		0	1	0
2	27	1	2	2	2	0	2	2	2	2	2		53	2	0	0	1	1	1	1	2018-07-14 16:45	2	2	2	2	3		2	1	1	2		7.3	2	2	1		1	2	1
1	69	1	1	1	2	0	2	1	2	2	2		52	1	1	0	1	1	1	1	2018-07-18 21:00	2	2	2	1	2		2	1	1	1		29.5	1	2	2	3	1	2	1
1	63	1	2	1	2	0	2	2	2	2	2		20	2	0	0	1	1	1	1	2018-07-21 11:35	2	2	1	2	1		2	2	2	2		16.1	2	2	1		1	2	1
1	63	1	2	1	2	0	1	2	2	2	2		38	1	1	0	1	1	1	1	2018-07-23 14:25	2	2	2	2	3		2	1	1	2		19.3	2	2	1		1	1	1
1	76	2	2	2	2	0	1	1	1	2	2	1	6	2	0	0	1	1	1	1	2018-07-25 01:40	2	2	2	2	3		2	1	1	1		16.6	2	2	2	3	1	2	1
1	41	2	1	1	2	0	2	2	2	1	2		12	2	0	0	1	1	1	1	2018-08-02 01:10	2	2	2	2	3	0	2	1	1	2		9.7	2	2	1		0	1	0
1	50	2	1	2	2	0	2	2	2	2	2		17	1	0	0	1	1	1	1	2018-08-02 22:20	2	2	2	1	2		2	2	2	2		13.1	2	2	2	1	1	2	1
1	57	2	1	1	2	1	2	1	1	2	2		8	1	0	0	1	1	1	1	2018-08-08 05:20	2	2	1	2	1	1	2	1	1	1		16.6	2	2	1		0	1	0
2	64	2	1	2	2	0	2	2	1	2	2		50	1	0	0	1	1	1	1	2018-08-17 00:00	2	2	2	1	2		2	1	1	2		17	2	2	1		1	2	1
1	87	1	1	1	2	0	2	1	2	2	2		12	1	1	0	1	1	1	1	2018-08-22 15:00	2	2	1	2	1		2	1	2	1		22.5	2	2	2	1	1	2	1
2	59	1	2	1	2	0	2	1	2	2	2		47	2	1	0	1	1	1	1	2018-09-04 20:20	2	2	2	2	3		2	1	2	1		20.9	2	2	2	3	1	2	1
1	35	2	1	1	1	0	2	2	2	2	2		23	1	1	0	1	1	1	1	2018-09-05 17:40	2	2	1	2	1		2	2	2	2		26.3	2	2	2	2	1	2	1
1	56	2	1	2	1	0	2	2	2	2	2		15	1	0	0	1	1	1	1	2018-09-07 15:15	2	2	2	1	2		2	2	2	2		15.2	2	2	1		0	1	0
1	73	2	1	2	2	0	1	1	2	2	2		35	1	1	0	1	1	1	1	2018-09-16 10:35	2	2	1	2	1		2	1	1	2		13.6	2	2	1		1	2	1
1	61	1	2	1	2	0	2	2	2	2	2		25	2	1	0	1	1	1	1	2018-09-22 20:10	2	2	1	2	1	0	2	1	1	2		6.6	2	2	1		1	1	1
2	73	1	1	1	2	0	2	1	1	2	2		20	2	0	0	1	1	1	1	2018-09-24 05:20	2	2	2	2	3		2	2	2	2		28.3	1	2	1		1	2	1
2	49	2	1	1	1	0	2	2	2	2	2		30	1	0	0	1	1	1	1	2018-10-01 16:35	2	2	1	2	1		2	1	1	2		13.7	2	2	1		0	1	0
1	78	1	1	1	2	0	2	2	1	2	2		44	1	0	0	1	1	1	1	2018-10-04 22:35	2	2	2	1	2	1	2	1	2	1		7	2	2	1		1	1	1
1	58	2	1	1	2	0	2	2	2	2	2		16	2	1	0	1	1	1	1	2018-10-12 13:50	2	2	2	2	3	0	2	2	2	2		10.4	2	2	1		1	1	1
2	68	2	1	1	2	0	2	2	2	2	1		21	1	0	1	1	1	1	1	2018-10-17 17:15	2	2	2	2	3	0	1	1	1	2		11.1	1	2	2	1	1	2	1
1	92	1	1	2	2	0	2	2	2	2	2		23	1	0	0	1	1	1	1	2018-10-20 14:00	2	2	1	2	1		2	1	2	1		28.7	2	2	1		1	2	1
1	84	2	1	1	2	0	1	1	2	2	2		18	2	0	1	1	2	2	1	2018-10-22 22:15	2	2	2	2	3	0	2	1	1	2		16.8	2	2	1		1	2	1
1	76	1	2	2	2	0	1	2	1	2	2		42	1	0	0	1	1	1	1	2018-11-03 05:05	2	2	1	2	1	1	2	1	1	2		23.4	2	2	2	3	1	2	1
1	82	2	1	1	1	0	2	2	1	2	2		10	1	0	0	1	1	1	1	2018-11-15 19:55	2	2	1	2	1		2	1	1	2		22.5	1	2	1		0	1	0
1	70	2	1	1	2	0	1	1	1	2	2	1	20	1	0	0	1	1	1	1	2018-11-30 03:00	2	2	2	1	2		2	1	1	1		27.8	1	2	2	3	1	2	1
1	84	1	1	1	2	0	2	2	1	2	2		45	1	0	0	1	1	1	1	2018-12-04 10:55	2	2	1	2	1	0	2	2	2	2		4.9	2	2	2	1	1	2	1
2	58	2	1	1	2	1	2	1	1	2	2		15	1	1	0	1	1	1	1	2018-12-08 12:40	2	2	2	2	3	0	2	1	1	1		31.3	2	1	2	3	1	2	1
2	54	1	1	1	2	1	2	1	1	2	1		15	1	0	0	1	1	1	1	2018-12-10 10:50	2	2	1	2	1		2	1	1	2		30.5	1	2	1		1	2	1

1	66	2	2	1	2	0	2	1	2	2	1		52	1	1	0	1	1	1	1	2018-12-11 15:00	2	2	2	2	3	0	2	2	2	2		2.6	1	2	2	1	1	2	1
1	56	1	1	1	2	0	2	1	1	2	2		45	2	0	0	1	1	1	1	2018-12-16 02:50	2	2	2	2	3		2	1	1	2		39.5	2	2	1		1	2	1
1	69	2	1	2	2	0	2	2	1	2	2		39	2	0	0	1	1	1	1	2018-12-18 19:00	2	2	2	2	3		2	1	1	2		31.9	2	2	2	2	1	2	1
1	30	1	1	1	1	0	2	2	2	2	2		14	1	0	0	1	1	1	1	2018-12-22 18:20	2	2	1	2	1	0	2	2	2	2		8.7	2	2	1		0	1	0
2	57	2	1	1	1	0	2	2	2	2	2		18	1	1	0	1	1	1	1	2018-12-31 13:00	2	2	1	2	1	0	2	2	2	2		13.9	2	2	1		0	1	0
1	67	2	1	2	2	0	2	1	2	2	2		33	1	0	0	1	2	2	1	2016-01-27 10:10	2	2	2	2	3		2	2	2	2		35.9	2	2	2	2	1	2	1
1	76	1	1	1	1	0	2	1	1	2	2		16	1	0	0	1	2	2	1	2016-05-18 12:52	2	2	2	2	3		2	2	2	2		27.5	2	1	2	1	1	2	1
1	52	1	2	2	2	0	2	2	2	2	2		70	1	0	0	1	2		1	2016-04-20 16:00	2	2	2	2	3		2	2	2	2		28.4	2	1	2	1	1	2	1
2	62	1	1	1	1	1	1	1	2	2	2		59	1	1	0	1	2	2	1	2016-02-15 22:20	2	2	2	2	3		2	1	2	1		3.3	1	2	2	3	1	2	1
2	86	1	2	1	2	0	2	1	1	2	1		32	1	0	0	1	2	2	1	2016-02-19 16:00	2	2	2	2	3		2	2	2	2		25.6	1	1	2	3	1	2	1
1	68	1	1	1	2	0	2	2	1	2	2		21	1	0	0	1	2	2	1	2016-02-26 23:45	2	2	2	2	3	0	2	1	1	2		7.5	2	2	1		0	1	0
2	69	2	1	1	2	1	2	1	1	2	2		32	1	0	0	1	2	2	1	2016-02-26 17:41	2	2	2	2	3		2	2	2	2		36	1	2	2	3	1	2	1
1	46	2	1	2	1	0	2	2	2	2	2		22	1	0	0	1	2	2	1	2016-03-07 14:00	2	2	2	2	3		2	2	2	2			2	2	2	1	1	2	1
1	69	1	1	1	2	0	1	1	2	2	2		195	1		0	1	2	2	1	2016-03-09 17:40	2	2	2	2	3	0	2	2	2	2		1.7	2	2	1		0	1	0
1	43	1	2	1	2	0	2	2	2	2	2		32	2	0	0	1	2	2	1	2016-03-28 03:20	2	2	2	2	3		2	2	2	2		1.3	1	2	2		1	2	1
1	79	2	1	1	2	0	2	1	2	2	2		29	2	0	0	1	2	2	1	2016-03-30 15:42	2	2	2	2	3		2	1	1	2		2	2	2	2	3	1	2	1
1	49	2	1	1	1	0	2	2	2	2	2		14	1	0	0	1	2	2	1	2016-04-18 17:25	2	2	1	2	1	1	2	2	2	2		1.4	2	2	1		0	1	0
1	77	2	1	1	1	0	1	1	1	2	2	1	28	1	0	0	1	2	2	1	2016-04-26 14:00	2	2	2	2	3	1	2	1	1	2		1.6	2	2	1		0	1	0
2	46	1	1	2	2	0	2	2	2	2	2		24	1	0	0	1	2	2	1	2016-04-27 08:20	2	2	2	2	3	0	2	1	1	1		1.5	2	2	1		0	1	0
2	80	1	1	1	2	0	2	1	2	2	2		63	1	0	0	1	2	2	1	2016-05-02 23:50	2	2	2	2	3		2	2	2	2		2.4	2	2	2	3	1	2	1
2	62	1	2	2	2	1	2	1	2	2	2		22	2	0	0	1	2	2	1	2016-05-03 15:44	2	2	2	2	3		2	1	1	2		3	2	2	2	2	1	2	1
1	53	1	1	1	2	0	2	1	2	2	2		41	1	0	0	1	2	2	1	2016-05-08 11:30	2	2	2	2	3		2	1	1	2		8	2	2	2	3	1	2	1
1	84	1	1	1	2	0	2	1	2	2	2		44	1	0	0	1	2		1	2016-05-11 16:30	2	2	2	2	3		2	1	1	2		9	2	2	2	3	1	2	1
1	60	1	2	2	1	0	2	1	2	2	2		16	1	1	0	1	2	2	1	2016-05-27 15:00	2	2	2	2	3	1	2	1	1	2		4	2	2	1		0	1	0
1	38	1	1	1	1	1	2	1	2	2	1		18	1	0	0	1	2	2	1	2016-05-31 18:10	2	2	2	2	3		2	2	2	2		22.8	2	1	1		0	1	0
1	74	2	1	1	2	0	2	1	2	2	2		12	1	0	0	1	2	2	1	2016-06-13 16:30	2	2	2	2	3		2	1	1	2		19.6	2	2	2	3	1	2	1
1	47	2	1	1	1	0	2	2	2	2	2		16	1	0	0	1	2	2	1	2016-06-24 18:50	2	2	2	2	3	0	2	1	1	2		0.5	1	2	1		0	1	0
2	50	1	2	2	2	0	2	2	2	2	2		9	2	0	0	1	2	2	1	2016-06-27 15:30	2	2	2	2	3	0	2	1	1	2		6.4	2	2	1		0	1	0
1	68	1	1	2	1	0	2	1	2	2	1		38	1	0	0	1	2	2	1	2016-07-04 16:35	2	2	2	2	3		2	2	2	2		2.2	1	1	2	3	1	2	1
1	77	1	2	1	2	0	2	1	2	2	2		25	2	1	0	1	2	2	1	2016-07-08 01:00	2	2	2	2	3		2	1	1	2		21.8	2	2	2	3	1	2	1
1	55	1	1	1	2	0	2	1	1	2	2		46	1	0	0	1	2	2	1	2016-07-16 10:20	2	2	2	2	3		2	2	2	2		49.5	1	1	2	3	1	2	1
1	55	1	1	1	2	0	2	1	1	2	2		24	1	0	0	1	2	2	1	2016-07-31 21:30	2	2	2	2	3		2	2	2	2		12.3	2	2	1		1	2	1
1	69	1	1	1	2	0	1	1	1	2	2	1	45	1	1	0	1	2	2	1	2016-08-05 11:30	2	2	2	2	3		2	2	2	2		40	2	1	2	3	1	2	1
2	64	1	1	2	1	0	2	1	1	1	2		26	1	0	0	1	2	2	1	2016-08-31 16:20	2	2	2	2	3		2	1	1	2		8.1	2	2	1		1	2	1
1	47	1	2	2	2	0	2	2	2	2	2		47	2	1	0	1	2	2	1	2016-08-31 19:15	2	2	2	2	3		1	1	1	2		13.3	2	2	2	3	1	2	1
2	70	1	1	1	2	0	2	1	1	2	2		33	1	0	0	1	2	2	1	2016-09-01 14:40	2	2	2	2	3		2	1	1	2		3.9	1	2	2	3	1	2	1
2	73	2	1	1	2	0	2	1	1	1	2		20	1	0	0	1	2	2	1	2016-09-05 23:00	2	2	2	2	3		2	1	1	2		14.7	2	2	1		1	1	1
1	77	2	1	1	2	0	1	1	2	2	2		17	1	0	0	1	2	2	1	2016-10-03 23:40	2	2	2	2	3		2	2	2	2		0	2	1	2	1	1	2	1
1	81	1	1	1	2	0	1	1	2	2	2		33	1	0	0	1	2	2	1	2016-10-07 11:05	2	2	2	2	3		2	2	2	2		0.1	1	1	2	3	1	2	1
2	46	2	2	1	2	1	2	1	1	2	2		19	2	0	0	1	2	2	1	2016-10-10 11:00	2	2	2	2	3		2	2	2	2		1.3	2	1	2	3	1	2	1
1	23	1	1	1	2	0	2	2	2	2	2		26	2	1	0	1	2	2	1	2016-10-15 04:20	2	2	2	2	3		2	1	1	2		12.7	2	2	1		1	1	1
1	62	2	2	2	2	0	2	2	2	2	2		16	2	1	0	1	2	2	1	2016-10-04 03:40	2	2	2	2	3		1	1	1	2		4.2	2	2	2	3	1	2	1
1	59	2	2	1	1	0	1	1	1	2	2		20	1	0	0	1	2	2	1	2017-01-15 11:20	2	2	1	2	1	0	2	2	2	2		2.6	2	2	1		0	1	0
1	71	1	1	1	2	0	2	1	1	2	2		32	1	0	0	1	2	2	1	2017-02-15 06:30	2	2	2	2	3		2	1	2	1		4.4	2	2	2	3	1	2	1
1	72	1	1	2	1	0	1	2	2	2	2	1	47	1	0	0	1	2	2	1	2017-02-17 20:00	2	2	2	2	3		2	1	1	2		1.5	2	2	2	3	1	2	1
2	72	1	1	2	2	0	2	2	2	2	2		37	1	0	0	1	2	2	1	2017-04-24 10:00	2	2	2	2	3		2	1	1	2		4.2	2	2	1		1	1	1
1	74	2	1	2	2	0	2	1	2	2	2		25	1	0	0	1	2	2	1	2017-05-29 16:50	2	2	2	2	3		2	2	2	2		2.6	2	2	1		0	1	0
1	74	1	1	1	1	0	1	1	2	2	2	1	12	1	0	0	1	2	2	1	2017-06-14 06:00	2	2	2	2	3		2	2	2	2		1.3	2	2	1		0	1	0
1	57	2	1	2	2	0	2	1	2	2	2		70	1	0	0	1	2	2	1	2017-07-02 20:30	2	2	2	2	3		2	2	2	2		0.3	2	1	2	3	1	2	1
1	56	1	1	1	1	0	2	2	1	2	2		190	1	0	0	1	2	2	1	2017-07-07 03:20	2	2	2	2	3		2	2	2	2		1.6	2	1	2	3	1	2	1
1	58	2	1	1	1	0	2	1	2	2	2		20	1	0	0	1	2	2	1	2017-07-07 16:00	2	2	2	2	3	0	2	2	2	2		0.5	2	2	1		0	1	0
2	27	1	2	2	2	0	2	2	2	2	2		25	2	1	0	1	2	2	1	2017-07-23 02:40	2	2	2	2	3		2	2	2	2		2	2	2	1		1	2	1
1	65	1	1	1	2	0	2	2	1	2	2		87	1	1	0	1	2		1	2017-08-06 02:30	2	2	2	2	3		2	2	2	2			2	1	2	3	1	2	1
1	63	2	2	2	1	0	1	2	1	2	2		44	1	0	0	1	2	2	1	2017-08-25 11:00	2	2	2	2	3		2	2	2	2		2.7	2	2	2	3	1	2	1
2	51	1	2	2	2	0	2	2	2	2	2		38	2	1	0	1	2	2	1	2017-08-29 18:00	2	2	2	2	3		2	2	2	2		3.1	2	2	2	3	1	2	1
1	64	1	1	2	2	0	2	2	1	2	2		8	1	0	0	1	2	2	1	2017-09-06 06:30	2	2	2	2	3		2	2	2	2		1.2	2	2	1		1	1	0
1	65	2	1	2	2	0	2	1	2	2	2		15	1	0	0	1	2		1	2017-09-15 20:30	2	2	2	2	3		2	2	2	2			1	1	2	3	1	2	1
1	51	1	1	1	1	0	2	2	2	2	2		7	1	0	0	1	2	2	1	2017-09-29 00:20	2	2	2	2	3		2	2	2	2		0.7	2	2	1		0	1	0
1	74	2	2	2	2	0	2	2	2	2	2		14	1	0	0	1	2	2	1	2017-10-04 20:40	2	2	2	2	3		2	2	2	2			2	2	2	3	1	2	1
1	74	1	2	2	2	0	2	2	2	2	2		28	1	1	0	1	2	2	1	2017-10-20 18:20	2	2	2	2	3		2	2	2	2		2.2	2	2	2	3	1	2	1
1	60	1	2	2	1	0	2	1	2	2	2		19	1	0	0	1	2	2	1	2016-11-23 19:00	2	2	2	2	3		2	2	2	2		1.7	2	1	2	3	1	2	1
1	81	1	1	2	2	0	2	2	2	2	2		23	2	0	0	1	2	2	1	2016-12-01 14:00	2	2	2	2	3		2	2	2	2		0.6	2	1	2	1	1	2	1
1	85	1	1	2	2	0	2	1	1	2	1		10	1	0	0	1	2	2	1	2016-12-06 11:30	2	2	2	2	3		2	1	1	2		0.1	1	1	2	3	1	2	1
1	40	1	2	1	2	0	2	2	2	2	2		70	2	1	0	1	2	2	1	2016-12-26 14:00	2	2	2	2	3		2	2	2	2		1.3	2	2	2	3	1	2	1
1	82	1	1	1	1	0	2	1	1	1	2		12	1	0	0	1	2	2	1	2016-11-23 15:10	2	2	1	2	1	1	2	2	2	2		2	2	2	1		0	1	0
1	53	2	1	1	1	0	1	1	2	2	2	1	18	2	0	0	1	2	2	1	2016-12-07 18:00	2	2	2	2	3		2	2	2	2		2.3	2	2	1		0	1	0
1	70	1	1	2	2	0	2	1	2	2	2		25	1	0	0	1	2	2	1	2017-01-31 23:40	2	2	2	2	3		2	1	1	2		0.8	2	1	1		0	1	0
1	54	1	1	2	1	0	2	1	2	2	2		22	1	0	0	1	2	2	1	2017-02-12 13:40	2	2	1	2	1	0	2	2	2	2		3.2	2	2	1		0	1	0
1	60	1	1	1	1	0	2	2	1	2	2		21	1	1	0	1	2	2	1	2017-02-12 00:50	2	2	2	2	3		2	2	2	2		2	99	2	2	3	1	2	1
2	71	1	1	2	1	0	2	1	1	1	2		18	1	0	0	1	2	2	1	2017-10-04 08:50	2	2	1	2	1	0	2	2	2	2		0.2	2	2	1		0	1	0
1	26	1	2	2	2	0	2	2	2	2	2		55	2	0	0	1	2	2	1	2018-03-27 16:51	2	2	2	2	3		2	2	2	2		2.6	2	2	2	2	1	2	1
2	57	1	1	1	2	0	1	1	2	2	1		43	1	0	0	1	2	2	1	2018-05-15 01:08	2	2	2	2	3		2	1	1	2			1	1	2	3	1	2	1
1	77	1	1	1	2	0	1	1	1	2	2	1	52	1	0	0	1	2	2	1	2017-11-02 14:15	2	2	2	2	3		2	2	2	2		0.4	2	1	2	1	1	2	1
1	74	1	1	1	2	0	1	2	2	2	2		13	1	0	0	1	2	2	1	2017-11-21 09:12	2	2	2	2	3		2	2	2	2		0.8	2	2	1		0	1	0
1	37	1	1	1	1	0	2	2	2	2	2		17	1	0	0	1	2	2	1	2017-11-27 08:20	2	2	2	2	3		2	2	2	2		0.2	2	2	1		0	1	0
1	55	1	1	1	1	0	2	2	2	2	2		46	1	0	0	1	2	2	1	2017-11-27 14:09	2	2	1	2	1	1	2	2	2	2		0.8	2	2	1		0	1	0
2	63	2	1	1	1	0	2	2	2	2	2		25	1	0	0	1	2	2	1	2017-12-03 11:03	2	2	2	2	3		2	1	1	2		0.6	2	2	1		1	1	1
1	62	1	1	1	2	0	2	1	2	2	2		50	1		0	1	2	2	1	2017-12-09 12:07	2	2	2	2	3		1	2	2	2		3.5	1	2	2	3	1	2	1
1	61	2	1	1	1	0	2	1	1	2	1		17	1	0	0	1	2	2	1	2017-12-17 01:54	2	2	2	2	3		2	2	2	2			1	2	1		0	1	0
1	75	2	1	2	1	0	2	2	2	2	2		11	1	0	0	1	2	2	1	2017-12-25 17:24	2	2	1	2	1	0	2	2	2	2		0.6	2	2	1		0	1	0
1	24	2	2	1	2	0	2	2	2	2	2		47	2	0	0	1	2	2	1	2018-01-19 15:12	2	2	2	2	3		2	2	2	2		2.1	2	2	2	2	1	2	1
2	63	1	2	2	2	0	2	1	2	2	2		35	2	1	0	1	2	2	1	2018-01-20 15:52	2	2	2	2	3		2	2	2	2		2.2	1	2	2	3	1	2	1
1	55	1	1	1	1	0	2	1	1	2	2		57	1	0	0	1	2	2	1	2018-01-19 16:48	2	1	2	2	3		2	2	2	2		3	2	2	2	3	1	2	1
1	70	1	2	2	2	0	2	2	1	2	2		38	1	0	0	1	2	2	1	2018-01-31 22:08	2	2	2	2	3		2	2	2	2		2.4	2	2	2	3	1	2	1
1	82	2	1	1	2	0	2	2	1	2	2		24	1	0	0	1	2	2	1	2018-02-09 16:27	2	1	2	2	3		2	2	2	2		0.8	2	2	1		0	1	0
1	78	2	1	1	2	0	2	1	1	2	2		25	1	0	0	1	2		1	2018-02-14 14:03	2	2	2	2	3		2	2	2	2		3.7	1	1	2	3	1	2	1
1	46	1	1	1	1	0	2	2	2	2	2		26	1	0	0	1	2	2	1	2018-03-12 13:37	2	1	2	2	3		2	2	2	2		1.7	2	2	1		0	1	0
1	79	1	2	1	2	0	2	2	2	2	2		19	1	0	0	1	2	2	1	2018-03-17 06:32	2	2	2	2	3		2	2	2	2		1.1	2	2	2	3	1	2	1
2	65	1	1	1	1	0	2	2	1	2	2		8	1	0	0	1	2	2	1	2018-03-21 12:10	2	1	2	2	3		2	2	2	2		1.4	2	2	1		0	1	0
1	62	2	1	1	1	0	2	1	1	2	2		66	1	0	0	1	2	2	1	2018-04-12 19:06	2	2	2	2	3		1	2	2	2		0.8	1	1	2	3	1	2	1
1	57	1	2	2	2	0	2	2	2	2	2		38	1	0	0	1	2	2	1	2018-04-26 13:56	2	2	2	2	3		1	1	1	2		2	2	2	1		1	1	1
1	54	1	1	1	1	0	2	1	2	2	2		30	1	0	0	1	2	2	1	2018-05-06 10:00	2	2	2	2	3		2	2	2	2		0.8	2	2	2	3	1	2	1
1	64	2	1	2	2	0	2	1	1	2	2		53	1	1	0	1	2	2	1	2018-09-30 12:23	2	1	2	2	3		2	1	1	2		4.2	2	1	2	1	1	2	1
1	59	1	1	1	1	0	2	2	2	2	2		18	1	0	0	1	2	2	1	2018-05-15 18:02	2	1	2	2	3		2	1	1	2			2	2	1		0	1	0
1	49	1	1	1	1	0	2	2	2	2	2		32	1	0	0	1	2	2	1	2018-05-17 19:00	2	2	2	2	3		1	1	1	2		1.5	2	2	1		1	1	1
1	70	2	2	2	1	0	2	2	2	2	2		18	1	0	0	1	2	2	1	2018-07-12 15:52	2	1	2	2	3		2	2	2	2			2	2	1		0	1	0
1	72	2	1	1	2	0	2	2	2	1	2		50	2	0	0	1	2	2	1	2018-07-17 14:54	2	2	2	2	3		2	1	1	2		2.7	2	1	2	3	1	2	1
1	80	2	1	2	2	0	2	2	2	2	2		48	1	1	0	1	2	2	1	2018-07-23 15:46	2	2	2	2	3		2	2	2	2			2	1	2	3	1	2	1
1	70	2	1	2	2	0	2	2	2	2	2		97	1	0	0	1	2	2	1	2018-07-27 18:14	2	2	2	2	3		1	1	1	2		3.5	2	1	2	3	1	2	1
1	58	2	1	1	2	0	2	2	2	2	2		10	1	0	0	1	2	2	1	2018-08-17 13:22	2	1	2	2	3		2	2	2	2		1.8	2	2	1		0	1	0
1	30	1	2	1	2	0	2	2	2	2	2		51	2	0	0	1	2	2	1	2018-08-22 14:28	2	2	2	2	3		1	2	2	2		0.8	1	1	2	3	1	2	1
1	55	2	1	1	1	0	2	2	2	2	2		29	1	1	0	1	2	2	1	2018-09-03 04:17	2	1	2	2	3		2	2	2	2		4	1	1	2	3	1	2	1
2	77	1	1	2	2	0	2	1	1	2	1		43	1	1	0	1	2		1	2018-09-15 19:42	2	2	2	2	3		2	2	2	2		8	2	1	2	3	1	2	1
1	54	2	1	1	2	0	2	1	2	2	2		7	2	0	0	1	2	2	1	2018-10-23 19:20	2	2	2	2	3		2	1	1	2		3.7	2	2	2	3	1	2	1
1	67	2	2	2	1	0	1	2	1	2	2		73	1	0	0	1	2	2	1	2018-11-02 07:50	2	1	2	2	3		1	2	2	2		0	2	1	2	1	1	2	1
1	55	2	1	1	2	0	2	2	2	2	2		3	2	0	0	1	2	2	1	2018-11-07 23:49	2	2	2	2	3		2	1	1	2		30	2	2	2	3	1	2	1
2	51	1	2	2	2	0	2	2	2	2	2		7	2	1	0	1	2	2	1	2018-12-01 04:33	2	2	2	2	3	0	2	2	2	2		2.3	2	2	1		1	1	1
2	43	2	1	1	2	1	2	1	1	2	2		2	2	0	0	1	2	2	1	2018-12-18 21:37	2	2	2	2	3		2	2	2	2		1.2	2	2	1		0	1	0
1	82	2	1	1	2	0	2	1	2	2	2		18	1	0	0	1	2	2	1	2018-12-28 16:28	2	2	2	2	3		2	2	2	2		1.5	2	2	1		1	1	1
1	36	2	2	1	2	0	2	2	2	2	2		70	2	1	0	1	1	1	1	2015-11-03 17:50	2	2	2	2	3		2	2	2	2		100	2	2	2	2	1	2	1
1	48	1	2	2	2	0	2	1	2	2	2		58	2	0	0	1	1	1	1	2015-12-02 00:05	2	2	2	2	3		2	2	2	2		0.7	2	1	2	2	1	2	1
1	58	1	1	2	2	0	1	2	2	2	2		31	1	0	0	1	1	1	1	2015-12-21 10:45	2	2	1	2	1		2	2	2	2		100	2	2	1		0	1	0
2	46	1	2	2	2	0	2	2	1	2	2		16	2	0	0	1	1	1	1	2015-12-28 01:35	2	2	2	2	3		2	2	2	2		62.6	2	2	1		1	2	1
2	56	1	1	1	1	0	2	2	2	2	2		19	1	0	0	1	1	1	1	2016-01-14 18:00	2	2	1	2	1		2	2	2	2		0.3	2	2	1		0	1	0
1	77	2	1	1	1	0	2	2	2	2	2		14	1	0	0	1	1	1	1	2016-03-02 18:28	1	2	2	1	2	1	2	1	2	1		41.5	1	1	2	4	1	2	1

1	43	1	2	1	2	0	2	1	2	2	2		40	2	0	0	1	1	1	1	2016-05-07 02:10	2	2	2	2	3	0	2	1	1	2			2	2	1		1	1	1
2	46	2	1	1	1	0	2	1	1	2	2		113	1	1	1	1	1	1	1	2016-05-24 19:12	1	2	1	2	1	1	2	2	2	2		54.7	2	1	2	3	1	2	1
2	23	2	1	1	2	0	2	2	2	2	2		12	2	0	0	1	1	1	1	2016-06-12 23:53	2	2	2	2	3		2	2	2	2		10.9	2	2	2	3	1	2	1
1	26	1	1	1	1	0	2	2	2	2	2		25	1	0	0	1	1	1	1	2016-07-20 08:15	2	2	1	2	1	0	2	2	2	2		17.7	2	2	1		0	1	0
2	65	2	1	1	2	0	2	1	1	2	1		16	1	0	1	1	1	1	1	2016-08-01 08:25	2	2	2	1	2	1	2	2	2	2			2	2	2	4	1	2	1
1	76	2	1	1	2	1	2	2	2	2	1		13	1	0	1	1	1	1	1	2016-11-02 00:45	2	2	1	2	1	0	2	2	2	2		90.3	2	2	1		0	1	0
1	75	2	2	2	2	0	2	1	2	2	2		52	2	0	1	1	1	1	1	2016-11-22 11:40	2	2	2	2	3	0	2	2	2	2		18.5	2	2	1		1	1	1
2	74	2	1	2	2	0	1	1	2	2	2		25	1	1	0	1	1	1	1	2016-11-27 17:51	2	2	1	2	1	1	2	2	2	2		17.5	2	2	1		1	1	1
1	48	1	1	2	1	0	2	2	2	2	2		67	1	0	1	1	1	1	1	2016-12-12 03:40	2	2	2	1	2	1	2	2	2	2		25.8	2	2	2	1	1	2	1
1	58	1	1	2	1	0	2	1	1	2	2		15	1	0	0	1	1	1	1	2017-01-02 04:57	2	2	1	2	1	0	2	2	2	2		20.1	2	2	1		0	1	0
2	54	1	2	1	2	0	2	1	2	2	2		37	2	0	0	1	1	1	1	2017-02-10 22:03	2	2	2	2	3		2	2	2	2		39	2	2	2	2	1	2	1
1	38	1	2	1	2	0	2	2	1	2	2		28	1	0	0	1	1	1	1	2017-03-13 14:32	2	2	1	2	1	1	2	2	2	2		34.9	1	2	2	2	1	2	1
1	44	1	2	1	2	0	2	2	2	2	2		65	2	1	0	1	1	1	1	2017-04-07 02:20	2	2	2	2	3	0	2	2	2	2		29.6	2	2	1		1	2	1
1	69	1	2	2	2	0	2	1	1	2	2		43	1	0	0	1	1	1	1	2017-04-23 10:28	2	2	1	2	1		2	2	2	2			2	1	2	1	1	2	1
1	65	1	1	1	1	0	2	2	2	2	2		56	1	1	0	1	1	1	1	2017-05-14 08:00	1	2	1	2	1	1	2	2	2	2			2	2	2	1	1	2	1
1	61	2	1	1	2	0	2	2	2	2	2		23	1	0	1	1	1	1	1	2017-06-05 11:15	2	2	1	2	1	0	2	2	2	2		14.3	2	2	1		1	2	1
1	67	2	1	1	2	0	2	2	2	2	2		40	2	1	0	1	1	1	1	2017-06-11 09:42	2	2	2	2	3		2	1	2	2		25.9	2	2	2	3	1	2	1
1	55	2	1	2	1	0	2	2	2	2	2		17	1	0	1	1	1	1	1	2017-06-20 13:16	2	2	2	1	2	1	2	2	2	2		23.8	2	2	1		0	1	0
1	47	1	2	1	2	0	2	2	2	2	2		44	2	1	0	1	1,5	1	1	2017-06-21 19:50	2	2	2	2	3		2	2	2	2		2	1	1	2	3	1	2	1
1	41	1	1	1	1	0	2	1	2	2	2		68	1	0	1	1	1	1	1	2017-07-20 11:00	2	2	2	1	2	1	2	2	2	2		1.8	1	2	1		0	1	0
1	60	2	2	2	2	0	2	2	1	2	2		36	1	0	0	1	1	1	1	2017-08-22 18:15	2	2	1	2	1		2	2	2	2		71.2	1	2	2	3	1	2	1
1	81	1	1	1	2	0	2	1	2	1	2		48	2	0	1	1	1	1	1	2017-09-09 02:36	2	2	2	2	3	1	2	2	2	2			2	2	2	4	1	2	1
1	69	1	1	1	2	0	2	1	2	2	2		4	2	0	1	1	1	1	1	2017-09-18 13:30	2	2	2	2	3	1	2	2	2	2		81.4	2	2	1		0	1	0
1	57	1	1	2	1	0	2	1	2	2	2		70	1	0	0	1	1	1	1	2017-09-20 03:33	2	2	1	2	1	1	2	2	2	2		19.1	2	2	2	2	1	2	1
2	19	2	1	2	1	0	2	2	2	2	2		16	1	0	1	1	1	1	1	2017-10-25 17:45	2	2	1	2	1	0	2	2	2	2			2	2	1		0	1	0
2	56	1	2	2	2	0	2	2	2	2	2		28	2	0	0	1	1	1	1	2017-10-26 13:22	2	2	2	2	3	0	2	2	2	2		16.6	2	2	2	2	1	2	1
1	46	2	1	2	1	0	2	2	2	2	2		40	1	0	1	1	1	1	1	2017-11-03 20:50	2	2	1	2	1	1	2	2	2	2			2	2	1		1	1	1
2	45	2	2	1	2	0	2	2	2	2	2		40	2	1	0	1	1	1	1	2017-11-16 16:00	2	2	2	2	3		2	2	2	2			2	2	1		1	2	1
2	62	2	1	2	2	0	2	2	2	2	2		66	1		0	1	1	1	1	2017-11-26 16:42	2	2	2	1	2		2	2	2	2			1	1	2	3	1	2	1
2	20	1	2	1	2		2	2	2	2	2		32	2	1	0	1	1	1	1	2017-11-27 23:42	2	2	2	2	3		2	2	2	2		9.6	2	2	1		1		
1	76	1	1	1	2	0	2	2	1	2	2		36	1	0	1	1	1	1	1	2017-12-10 01:29	2	2	2	2	3	0	2	2	2	2			2	2	2	4	1	2	1
2	85	1	1	2	2	1	1	1	1	2	1	1	70	1	0	1	1	1	1	1	2017-12-22 15:35	2	2	1	2	1	1	2	2	2	2			1	1	2	1	1	2	1
2	76	1	1	1	2	0	2	2	1	2	2		36	1	1	1	1	1	1	1	2017-12-25 18:47	2	2	2	2	3	0	2	2	2	2			1	1	2	3	1	2	1
1	30	1	1	1	1	1	2	2	2	2	2		104	1	1	0	1	1	1	1	2018-01-19 16:43	2	2	1	2	1	1	2	2	2	2			1	1	2	1	1	2	1
1	58	1	1	1	2		2	2	2	2	2		78	2	1	1	1	1	1	2		2	2	2	2	3		2	2	2	2			2	1	2	3	1	2	1
1	59	1	1	1	1	0	2	2	2	2	2		13	1	0	1	1	1	1	1	2018-02-10 01:36	2	2	1	2	1	0	2	2	2	2			2	2	1		0	1	0
1	65	1	1	1	2	1	2	1	1	1	2		62	2	0	1	1	1	1	1	2018-02-19 20:35	2	2	2	2	3	0	2	2	2	2			2	1	2	3	1	2	1
1	59	2	2	2	2		2	2	2	2	2		59	1	0	0	1	1	1	1	2018-02-24 11:00	2	2	2	1	2		2	2	2	2		31	2	1	1		1		
2	82	2	1	1	2	0	2	2	2	2	2		66	1	0	1	1	1	1	1	2018-02-27 21:15	2	2	2	1	2	1	2	2	2	2		5.1	2	1	2	1	1	2	1
2	83	2	1	1	2	0	2	2	2	2	2		1	2	0	1	1	1	1	1	2018-03-03 20:10	2	2	2	2	3		2	1	1	2		13.7	2	2	1		1	1	1
1	64	1	2	1	2	0	1	2	1	2	2		35	2	0	1	1	1	1	1	2018-03-07 06:26	2	2	2	2	3	1	2	1	1	2			2	2	1		1	2	1
1	72	2	1	2	1	0	1	1	2	2	2		31	1	0	1	1	1	1	1	2018-04-03 11:25	2	2	1	2	1	1	2	1	1	2		23.5	2	2	1		1	2	1
2	55	2	1	1	1	0	2	2	2	2	2		22	1	0	1	1	1	1	1	2018-04-09 16:05	2	2	1	2	1	0	2	2	2	2			2	2	1		0	1	0
1	76	2	1	1	2	0	2	2	2	2	2		9	2	0	0	1	1	1	1	2018-04-24 20:19	2	2	2	2	3	0	2	1	1	2			2	2	2	2	1	2	1
1	56	2	1	1	1	0	2	2	2	2	2		12	1	0	1	1	1	1	1	2018-04-25 14:00	2	2	2	1	2	0	2	2	2	2		0.4	2	2	1		0	1	0
1	59	2	1	1	1	0	2	1	2	2	2		20	1	0	1	1	1	1	1	2018-04-30 20:12	1	2	2	1	2	0	2	1	1	2		26.7	2	1	1		0	1	0
1	48	1	2	1	1	0	1	2	1	2	2		50	1	0	0	1	1	1	1	2018-05-17 09:31	2	2	1	2	1	0	2	1	1	2		3	1	1	2	1	1	2	1
1	59	2	1	1	1	0	1	2	1	2	2	1	9	1		1	1	1	1	1	2018-05-20 15:15	2	2	2	2	3	1	2	2	2	2		10.8	2	2	1		0	1	0
1	38	1	1	2	1	0	2	2	2	2	2		60	1	0	0	1	1	1	1	2018-05-27 13:40	2	2	2	1	2	1	2	2	2	2			2	2	1		0	1	0
1	45	2	1	1	1	0	2	2	2	2	2		21	1	0	1	1	1	1	1	2018-05-29 13:31	2	2	2	1	2	1	2	2	2	2			2	2	1		0	1	0
2	54	2	1	1	1	0	2	2	2	2	2		16	1	0	1	1	1	1	1	2018-06-19 17:12	2	2	1	2	1	1	2	2	2	2			2	2	1		0	1	0
1	47	2	1	1	1	0	2	2	2	2	2		11	1	1	0	1	1	1	1	2018-07-05 12:35	2	2	2	1	2	0	2	2	2	2			2	2	1		0	1	0
1	66	2	1	1	2	0	2	1	2	2	2		11	1	0	1	1	1	1	1	2019-07-12 19:15	2	2	1	2	1	0	2	2	2	2			2	2	1		1	2	1
1	57	1	1	1	2	0	1	2	2	2	2		8	2	0	0	1	1	1	1	2018-07-19 15:58	2	2	2	2	3		2	2	2	2			2	2	1		0	1	0
2	58	1	2	2	1	1	2	1	1	2	2		15	1	0	0	1	1	1	1	2018-07-20 05:50	2	2	2	1	2	1	2	2	2	2			1	1	2	1	1	2	1
1	69	1	1	1	1	0	2	1	2	2	2		10	1	0	1	1	1	1	1	2018-08-03 20:45	2	2	2	1	2	1	2	2	2	2			2	2	1		0	1	0
1	59	1	2	1	1	0	1	2	2	2	2		14	1	0	1	1	1	1	1	2018-08-07 16:40	2	2	1	2	1		2	2	2	2			2	2	1		0	1	0
1	77	2	1	1	2	0	2	2	1	2	2		32	2	0	0	1	1	1	1	2018-08-09 20:20	2	2	2	2	3	0	2	1	1	1			2	1	2	3	1	2	1
1	59	1	1	1	2	0	2	2	2	2	2		42	2	0	1	1	1	1	1	2018-09-15 20:27	2	2	2	2	3		1	2	2	2			2	2	2	1	1	2	1
1	48	2	1	2	2	0	2	2	2	2	2		17	2	0	1	1	1	1	1	2018-09-21 09:09	2	2	2	2	3	0	1	1	1	2			2	2	1		0	1	0
1	51	2	2	2	2	0	2	2	2	2	2		38	1		0	1	1	1	1	2018-09-26 17:09	2	2	1	2	1	0	2	1	1	2			1	2	2	3	1	2	1
1	67	2	1	1	1	0	2	2	2	2	2		9	1	0	1	1	1	1	1	2018-10-04 19:05	2	2	2	1	2	0	2	2	2	2			2	2	1		0	1	0
2	63	2	1	1	2	0	2	1	2	2	2		12	1	0	1	1	1	1	1	2018-10-21 09:10	2	2	2	2	3	0	2	1	2	1			2	2	2	3	1	2	1
1	83	2	1	2	1	0	1	1	1	2	2		11	1	0	1	1	1	1	1	2018-11-03 17:40	2	2	2	1	2	1	2	2	2	2			2	2	1		0	1	0
1	59	1	2	2	2		2	1	1	2	1		50	2	0	1	1	1	1	1	2018-11-20 01:10	2	2	2	2	3	1	2	2	2	2			1	1	2	3	1	2	1
1	78	1	1	2	1	0	1	2	2	2	2		20	1	0	0	1	1	1	1	2018-11-26 16:50	1	2	1	2	1	1	2	2	2	2			1	1	2	1	1	2	1
1	56	2	1	1	1		2	1	2	1	2		16	1	0	1	1	1	1	1	2018-12-10 06:30	2	2	2	2	3	0	2	2	2	2			2	2	1		1		
1	80	2	2	1	2	0	2	2	2	2	2		35	1	0	0	1	1	1	1	2018-12-17 16:25	2	2	1	2	1	1	2	2	2	2			2	1	2	3	1	2	1
1	48	1	2	2	2		2	2	2	2	2		14	2		1	1	1	1	1	2018-12-26 10:20	2	2	2	2	3		2	2	2	2			2	2	1		1		
2	51	2	1	1	2	0	2	2	2	2	2		83	1	1	0	1	1	1	1	2018-10-17 12:10	1	2	2	2	3	0	2	2	2	2			2	2	1		0	1	0
1	82	2	1	2	2	0	2	1	1	1	2		8	2	1	0	1	1	1	1	2016-04-27 21:10	2	2	2	2	3	0	2	1	2	1		28	2	2	1	1	1	1	1
1	35	2	2	1	2	0	2	2	2	2	2		36	2	1	0	1	1,5,6	1	1	2016-06-27 20:30	2	2	2	2	3	0	2	1	1	2		12.9	2	2	1		1	2	1
1	88	1	1	1	2	0	2	1	1	2	2		24	1	0	1	1	1	1	1	2016-10-25 15:33	2	2	2	2	3		2	1	1	2		29.3	2	2	2	4	1	2	1
1	68	2	2	2	2	0	2	2	2	2	2		18	2	0	0	1	1	1,6	1	2016-12-22 17:15	2	2	2	2	3	0	2	1	1	2		59.4	2	2	1		0	1	0
1	60	2	1	1	1	0	2	1	2	2	2		9	1	0	0	1	2	2,5	1	2017-05-01 13:25	2	2	2	1	2	0	2	1	1	2		22.6	2	2	1		0	1	0
1	28	2	1	1	2	0	2	2	2	2	2		39	1	0	0	1	2,5	2,5	1	2017-05-14 17:30	2	2	2	2	3	0	2	1	1	2		20.8	2	2	1		0	1	0
1	83	2	1	1	1		2	1	2	2	2		11	1	0	0	1	1,5,6	1	1	2017-05-25 03:30	2	2	2	2	3	0	2	2	2	2		2.8	2	2	1		1	1	
2	48	2	2	2	2	0	1	1	2	2	2		2	1	0	0	1	1,5	1	1	2017-07-16 15:20	2	2	1	2	1	1	2	1	1	2		31.2	2	1	1		0	1	0
1	54	2	1	1	1	0	2	1	2	2	2		47	1	0	0	1	2	2	1	2017-09-19 11:00	2	2	2	1	2	1	2	1	2	1		28.3	2	2	1		0	1	0
1	50	2	2	2	1		2	1	2	2	2		17	1	0	0	1	1	1	1	2017-09-24 05:41	2	1	2	1	2	1	2	1	2	2		13.1	2	2	1				
1	76	1	2	2	2	0	2	1	2	1	2		49	1	0	0	1	1,6	1	1	2017-11-13 14:40	2	2	2	2	3	0	2	1	1	2		32.2	2	1	2	4	1	2	1
1	30	2	2	1	1		2	1	2	2	2		32	1	1	0	1	1,2,6	1	1	2017-12-01 03:40	2	2	2	2	3	0	2	1	1	2		28.2	2	2	1		1		
1	64	1	2	2	2	0	2	2	2	2	2		28	2	0	0	2	4,5	4	1	2016-05-29 17:35	2	2	2	2	3		2	2	2	2		13.7	2	2	2	2	1	2	1
1	65	2	1	1	1	0	2	2	1	2	2		32	1	0	1	3	5	5	1	2016-07-20 17:30	2	2	1	2	1	0	2	1	1	2		5.1	2	2	1		0	1	0
1	48	2	2	2	2	0	2	1	2	2	2		40	1	0	0	1	2,6	2	1	2015-12-03 22:20	1	2	2	1	2	1	1	2	2	2		25.7	2	2	2	3	1	2	1
1	58	1	2	2	2	0	2	2	1	2	2		24	2	0	0	1	2	2	1	2015-12-25 20:20	2	2	2	2	3		2	2	2	2		23.5	2	2	1		0	1	0
1	48	2	2	1	2	0	2	2	2	2	2		46	2	0	0	1	2,6	2	1	2016-01-25 18:05	2	2	2	2	3	0	2	2	2	2		1	2	2	2	4	1	2	1
1	45	2	2	1	2	0	2	2	2	2	2		47	1	0	1	1	2,5,6	2	1	2016-02-06 17:20	2	2	2	2	3		2	2	2	2		2.9	2	2	2	3	1	2	1
1	61	1	1	1	1	0	2	2	2	2	2		18	1	0	0	1	2,6	2	1	2016-02-06 07:50	2	2	1	2	1	0	2	2	2	2		11.9	2	2	1		0	1	0
1	71	2	1	1	1	0	2	1	1	2	2		13	1	0	1	1	2,6	2	1	2016-02-14 23:00	2	2	2	1	2	1	2	2	2	2		45.2	2	2	1		0	1	0
1	18	2	1	1	1	0	2	2	2	2	2		12	2	0	1	3	6,8	6	1	2016-05-11 17:00	2	2	2	2	3		2	2	2	2		14	2	2	1		0	1	0
2	73	1	2	1	2	0	2	1	1	2	2		60	1	0	1	1	2,6	2	1	2016-05-20 17:50	2	2	2	2	3	0	2	2	2	2		2.1	2	1	2	1	1	2	1
2	57	2	1	1	1	0	2	2	2	2	2		12	1	0	1	1	2,5	2	1	2016-08-20 01:10	2	2	2	1	2		2	1	1	2		10.3	2	2	1	1	1	1	1
1	54	1	2	1	1	0	2	2	2	2	2		28	1	0	0	1	2,8	2	1	2016-10-03 10:09	2	2	2	1	2		2	1	1	2		18.7	2	2	1	4	0	1	0
1	53	2	1	2	2	0	1	1	2	2	2		27	1	0	0	1	2,8	2	1	2016-10-14 18:31	2	2	2	2	3	0	2	2	2	2		5.2	2	2	1		0	1	0
2	52	1	1	1	1	0	2	2	2	2	2		48	1	1	0	1	2	2	1	2016-10-23 04:53	2	2	2	2	3		2	2	2	2		33.8	2	2	2	3	1	2	1
1	55	1	2	2	2	0	2	1	2	2	2		36	1	0	0	1	2	2	1	2016-11-07 03:55	2	2	2	2	3	1	2	2	2	2		2	2	2	2	3	1	2	1
1	61	2	1	1	2	0	2	2	1	2	2		13	1	0	0	1	2,6,7,8	2	1	2016-11-09 06:53	2	2	1	2	1	0	2	2	2	2		8.5	2	2	1		0	1	0
2	44	1	2	1	2	0	2	2	2	2	2		6	1	0	0	1	2,6	2	1	2016-11-16 03:00	2	2	2	2	3	0	2	2	2	2			2	2	1		0	1	0
1	52	2	1	1	2	0	2	1	1	2	2		30	2	0	0	1	2	2	1	2016-12-28 17:50	2	2	2	2	3	1	2	2	2	2		35.3	1	2	1		1	2	1
2	87	1	2	1	2	0	2	1	2	2	1		50	1	1	0	1	2	2	1	2017-01-06 06:00	2	2	2	2	3		2	1	1	2		13.5	2	2	1		0	1	0
2	48	2	1	1	2	1	2	1	1	2	1		37	2	0	0	1	2,5	2,5	1	2017-03-10 00:25	2	2	2	2	3		2	2	2	2		4.8	2	1	2	1	1	2	1
1	72	2	1	1	2	0	2	2	2	2	2		13	2	0	0	1	2,5	2,5	1	2017-03-23 18:11	2	2	2	2	3	0	2	1	1	2		14.3	2	2	1		1	1	1
1	61	2	1	1	2	0	2	2	1	2	2		10	1	0	0	1	2,5	2,6	1	2017-03-26 16:15	2	2	2	1	2	1	2	2	2	2		9.4	2	2	1		1	1	1
1	75	2	2	1	2	0	2	2	1	2	2		43	1	0	0	1	1	2	1	2017-05-07 05:49	2	2	2	2	3		2	2	2	2		1.4	99	2	2	1	1	2	1
1	37	1	2	2	2	0	2	2	2	2	2		41	2	0	0	1	2	2	1	2017-05-25 08:08	2	2	2	2	3		2	2	2	2		0.1	2	1	2	1	1	2	1
1	63	1	1	1	1	0	2	1	2	2	2		65	1	0	0	1	2	2	1	2017-06-09 13:27	2	2	2	2	3	0	2	1	2	1			2	1	2	1	1	2	1
1	25	1	1	1	2	0	2	2	2	2	2		24	2	1	0	1	1	1	1	2017-07-25 17:15	2	2	2	2	3		2	1	2	1		0.3	2	1	2	1	1	2	1
2	39	1	2	1	2	0	2	2	2	2	2		14	2	0	0	1	2	2	1	2018-08-05 18:00	2	2	2	2	3		2	1	2	1		0.1	2	1	2	4	1	2	1
1	54	2	1	1	2	0	2	2	2	2	2		22	1	0	0	1	2	2	1	2018-10-16 02:02	2	2	2	2	3	0	2	1	2	1		27.4	2	2	1		1	1	1
1	24	1	2	2	2	0	2	2	2	2	2		10	2	1	0	1	2	2	1	2018-11-16 00:56	2	2	2	2	3		2	1	2	1		53.5	2	2	2	3	1	2	1
2	37	2	1	1	2	0	2	2	2	2	2		21	2	1	1	1	1,5,8	1	1	2016-05-16 13:00	2	2	2	2	3		2	1	1	2		12	2	2	2	2	1	2	1
1	49	1	1	1	1	0	1	2	2	2	2	1	20	1	1	1	1	1	1	1	2016-07-17 03:40	1	2	2	1	2	1	2	1	1	2		13.9	2	2	1	4	0	1	0
1	69	2	2	2	2		2	2	2	2	2		16	2		1	1	1	1	1	2016-06-14 09:05	2	2	2	2	3	0	2	2	2	2		6.9	2	2	1		0		
2	74	2	1	2	2		1	1	1	2	2	1	28	1	0	0	1	1	1	1	2016-06-26 18:05	2	2	1	2	1		2	1	2	2		10.7	2	2	1		1		

1	27	2	1	2	2		2	2	2	2	2		69	2	0	1	1	1	1	1	2016-07-25 01:36	2	2	2	2	3		2	2	2	2		28.2	1	2	1		1		
1	52	2	1	1	1		2	2	2	2	2		26	1	0	1	4	5,8		1	2016-07-25 13:05	2	2	1	2	1	1	2	2	2	2		8.2	2	2	1		1		
2	52	1	2	1	2	0	2	2	2	2	2		18	2	0	0	1	1,6,8	1	1	2016-09-16 01:50	2	2	2	2	3	0	2	1	1	2		17.7	2	2	1		0	1	0
1	43	2	1	1	1		2	2	2	2	2		29	1	0	1	1	1,5,6,8	1,5,8	1	2016-09-19 09:50	2	2	1	2	1	1	2	2	2	2			2	2	1		0		
1	28	2	2	1	1	0	2	2	2	2	2		16	1	0	1	1	1,5,6	1	1	2016-03-02 07:55	2	2	1	2	1	1	2	1	2	2		19.9	2	2	1		0	1	0
2	73	1	1	1	2	0	1	1	2	2	2	1	15	2	0	1	1	1,5,6	1,6	1	2016-03-19 15:05	2	2	2	2	3	0	2	1	1	1		31.3	1	2	2	4	1	2	1
2	60	1	2	2	2	0	2	2	1	2	2		46	1	0	0	1	1	1	1	2016-04-06 20:40	2	2	2	2	3	0	2	1	2	2		14.7	2	2	1		1	2	1
2	80	2	1	1	2	0	1	1	1	2	1		22	2	0	1	1	1,5	1	1	2016-04-15 02:00	2	2	2	2	3	1	2	2	2	2		22.6	1	2	2	2	1	2	1
2	43	1	2	1	2	0	2	2	2	2	2		43	2	1	1	1	1	1	1	2016-07-01 17:46	2	2	2	2	3	1	1	2	2	2		39.1	1	2	1		1	2	1
2	29	1	2	1	2	0	2	2	2	2	2		33	2	0	1	1	1	1	1	2016-07-24 02:05	2	2	2	2	3	1	2	1	1	2		27.7	2	2	1		1	2	1
2	72	1	2	2	1	0	2	1	1	2	2		68	1	0	1	1	1		1	2016-08-31 01:25	2	2	2	2	3		2	2	2	2		0	1	1	2	1	1	2	1
2	48	1	2	2	2	0	2	2	2	2	2		38	1	1	1	1	1	1	1	2016-10-28 23:35	2	2	2	2	3	1	2	2	2	2		20.8	2	2	2	2	1	2	1
1	50	2	1	1	1	0	2	1	2	2	2		16	1	0	1	1	1	1	1	2016-11-13 19:20	2	2	2	1	2	1	2	2	2	2		20.1	2	2	1		0	1	0
1	46	2	2	2	2	0	2	2	2	2	2		23	1	0	1	1	1,5	1	1	2017-01-20 05:24	2	2	2	2	3	0	2	2	2	2		23.7	2	2	1		0	1	0
1	63	2	2	2	2	0	2	2	2	2	2		9	1	0	0	1	1	1	1	2017-01-24 01:45	2	2	2	2	3	0	2	2	2	2		12.7	2	2	1		0	1	0
2	68	1	2	1	2	0	2	1	1	1	1		60	1	0	1	1	1	1	1	2017-01-28 13:20	2	2	2	2	3	0	2	2	2	2		15.3	1	2	2	1	1	2	1
1	60	2	2	2	2	0	2	2	2	2	2		38	1	1	1	3	5	5	1	2017-02-04 10:00	2	2	1	2	1	0	2	2	2	2		14	2	2	1		1	2	1
1	82	1	1	2	2	0	2	2	2	1	2		36	1	0	0	1	1	1	1	2017-02-06 00:00	2	2	2	2	3	1	2	2	2	2		15	1	2	1		1	2	1
2	53	2	1	1	1	0	2	1	2	2	2		27	1	0	1	1	1,5	1	1	2017-03-30 21:44	2	2	1	2	1	0	2	2	2	2		10.8	2	2	1		0	1	0
1	39	2	2	1	1	0	2	2	2	2	2		14	1	1	1	1	1,5	1	1	2017-04-20 17:00	2	2	1	2	1	0	2	2	2	2		13.1	2	2	1		0	1	0
1	74	1	1	2	2	0	1	2	2	1	1	1	38	1	0	1	1	1	1	2		2	2	2	2	3		2	2	2	2		21.1	2	1	2	1	1	2	1
1	25	1	1	1	2	0	2	2	2	2	2		160	1	0	1	1	1,5	1	1	2017-05-13 07:35	1	2	2	2	3	0	2	2	2	2			1	2	2	1	1	2	1
1	42	2	1	1	2	0	2	2	2	2	2		12	2	0	1	1	1,6	1	1	2017-05-24 20:15	2	2	2	2	3	0	2	2	2	2		10.5	2	2	1		1	1	1
2	29	2	2	2	2	0	2	2	2	2	2		16	2	0	1	1	1,5	1,5	1	2017-05-26 19:20	2	2	2	2	3	1	2	2	2	2		5.5	2	2	1		1	1	1
1	45	1	1	1	1	0	2	1	2	2	2		42	1	0	1	1	1	1	1	2017-06-02 05:35	2	2	1	2	1	0	2	2	2	2		28.8	2	2	1		1	2	1
1	64	1	2	2	2	0	1	1	1	2	1	1	23	1	0	1	1	1	1	1	2017-06-05 00:15	2	2	2	2	3	1	2	2	2	2		5.4	1	2	1		1	2	1
1	64	2	1	1	1	0	2	2	2	2	2		20	1	0	1	1	1	1	1	2017-04-22 19:00	2	2	2	1	2	0	2	2	2	2		22.4	2	2	1		0	1	0
1	52	1	1	1	1	0	2	2	2	2	2		57	1	0	1	1	1	1	1	2017-06-10 12:19	2	2	2	1	2	1	2	2	2	2		0.2	2	2	1		1	2	1
1	44	1	2	2	1		2	2	1	2	1		33	1	0	1	1	1,5	1	1	2017-06-19 23:19	2	2	2	2	3	1	2	2	2	2		17.6	1	2	1		1		
1	45	1	1	2	2	0	2	2	2	2	2		28	2	0	1	1	1	1	1	2017-06-28 01:20	2	2	2	2	3	1	2	2	2	2		4.1	1	1	2	3	1	2	1
1	39	1	2	1	2	0	2	2	2	2	2		32	2	0	1	1	1	1	1	2017-07-02 13:00	2	2	2	2	3		2	2	2	2		19.3	2	2	2	2	1	2	1
2	74	1	2	2	2	0	2	2	2	2	2		36	1	0	1	1	1	1	1	2017-07-29 16:00	2	2	2	2	3		2	1	2	1		9.9	1	2	2	3	1	2	1
1	84	1	2	2	2	0	2	1	2	2	2		27	2	0	1	1	1	1	1	2017-08-08 23:45	2	2	2	2	3	0	2	2	2	2		0.9	2	2	2	2	1	2	1
2	82	2	1	1	2	0	2	1	1	1	2		35	1	0	1	1	1	1	1	2017-08-15 01:40	2	2	2	2	3	1	2	1	2	1		15.6	1	2	1		1	2	1
2	66	1	2	2	2	0	2	2	2	2	2		54	2	0	0	1	1	1	2		2	2	2	2	3		2	2	2	2		0.1	2	2	2	3	1	2	1
1	77	1	1	1	2	0	1	1	1	2	1	1	21	2	0	1	1	1	1	1	2017-09-11 04:05	2	2	1	2	1	1	2	2	2	2		3.1	1	2	1		0	1	0
2	82	1	2	2	2	0	1	1	1	2	2	1	33	1	0	1	1	1,5	1	1	2017-09-19 08:15	2	2	2	2	3	1	2	2	2	2		25.7	1	2	2	2	1	2	1
1	62	2	1	1	2	0	2	1	1	2	1		29	2	0	1	1	1	1	1	2018-04-30 01:00	2	2	1	2	1	1	2	2	2	2		8.3	1	2	1		1	2	1
1	62	2	2	1	2	0	2	1	2	2	2		12	2	0	1	1	1	1	1	2018-05-02 22:40	2	2	2	2	3	0	2	2	2	2		16.1	1	2	2	4	1	2	1
2	87	2	1	1	2	0	1	1	1	2	2	1	11	1	0	1	1	1	1	1	2018-05-06 01:05	2	2	2	1	2	1	2	2	2	2		3.4	2	2	1		0	1	0
1	60	1	2	1	2	0	2	2	2	2	2		53	2	0	0	1	1	1	1	2018-05-11 00:10	2	2	2	2	3		2	2	2	2		33.4	2	2	2	4	1	2	1
2	26	1	2	1	2	0	2	2	2	2	2		37	2	0	1	1	1	1	1	2018-05-22 18:45	2	2	2	2	3	1	2	2	2	2		7.2	2	2	2	2	1	2	1
1	75	2	2	1	2	0	2	2	2	1	2		44	1	0	1	1	1	1	2		2	2	2	2	3	0	2	2	2	2		0	2	2	2	3	1	2	1
1	62	2	1	1	2	1	2	2	2	2	2		13	1	0	1	1	1	1	1	2018-06-24 23:05	2	2	2	1	2	0	2	2	2	2		8.9	2	2	1		0	1	0
1	77	2	1	1	2		2	2	2	2	2		27	2	0	0	1	1,5	1,5	2		2	2	2	2	3		2	2	2	2		7.9	2	2	1		1		
2	26	1	2	1	2	0	2	2	2	2	2		35	2	0	1	1	1	1	2		2	2	2	2	3		2	2	2	2		0	2	2	1		1	1	1
1	81	2	2	1	2	0	2	1	1	1	2		41	2	1	1	1	1,5	1	2		2	2	2	2	3		2	2	2	2		0.2	2	2	1		1	2	1
1	90	1	1	1	2	0	1	1	1	2	1	1	37	1	0	1	1	1	1	2		2	2	2	2	3		2	2	2	2		16.3	1	2	2	3	1	2	1
1	65	2	1	1	1	0	1	2	2	2	2		24	1	1	1	1	1,5	1,5	1	2018-12-31 21:10	2	2	2	2	3	1	2	2	2	2		0.9	2	2	1		0	1	0
1	64	1	1	1	2	0	2	2	1	2	2		66	2	0	1	1	1	1	1	2016-02-07 10:30	2	2	2	2	3	0	1	2	2	2			2	1	2	4	1	2	1
1	46	1	1	2	2	0	2	2	2	2	1		62	2	0	1	1	2	2	1	2016-02-12 02:50	2	2	2	2	3	0	2	2	2	2		6.4	1	2	2	3	1	2	1
2	90	1	1	1	2	0	2	1	2	2	2		40	1	0	1	1	2		1	2016-02-13 06:05	2	2	2	2	3		2	2	2	2			2	1	2	3	1	2	1
1	72	1	1	2	2		2	1	1	2	2		32	2		0	2	4	4	1	2016-02-04 21:22	2	2	2	2	3	0	2	1	1	1			2	2	1		1		
1	59	1	1	1	2	0	2	1	1	1	1		21	2	0	0	1	2	2	1	2016-02-15 17:30	2	2	2	2	3	0	1	1	2	1		26.1	1	2	1		1	1	0
1	67	1	1	1	2	0	2	2	1	2	2		32	1	0	0	2	4	4	1	2016-03-12 18:09	2	2	2	2	3	0	1	1	1	2		38.8	2	2	2	1	1	2	1
1	64	2	2	2	2	0	2	2	2	2	2		60	2	0	0	2	4	4	1	2016-03-23 15:40	2	2	2	2	3		2	1	1	2		11.5	2	2	1		1	1	1
1	73	1	1	2	2	0	2	2	2	2	2		20	2	0	0	1	2	2	1	2016-03-10 16:15	2	2	2	2	3	0	2	1	2	1		1.3	2	1	2	3	1	2	1
1	84	1	1	1	2		1	1	1	2	2	1	48	1	0	0	3	2,4	4	1	2016-02-19 03:20	2	2	2	2	3	1	2	2	2	2		11.7	2	2	1		1		
1	78	2	1	1	2	0	2	2	2	2	2		41	2	1	0	1	2	2	1	2016-01-15 15:20	2	2	2	2	3	1	2	2	2	2		0.3	2	2	1		1	1	1
1	76	2	1	2	2	0	2	1	2	2	2		47	2	0	1	2	4	4	1	2016-04-06 20:35	2	2	2	2	3		2	2	2	2		10.1	2	2	2	3	1	2	1
1	57	2	1	1	2	0	2	2	2	2	2		16	2	1	1	1	2	2	1	2016-04-21 22:30	2	2	2	2	3	0	1	1	1	2		5.5	1	2	2	3	1	2	1
1	60	2	1	1	2	0	2	2	1	2	2		33	2	0	0	1	2	2	1	2016-03-10 23:55	2	2	2	2	3	0	2	1	2	1		11.4	2	2	2	3	1	2	1
2	58	2	1	1	2	0	2	1	1	2	2		12	1	0	0	2	4	4	1	2016-01-04 15:42	2	2	2	2	3	0	2	1	1	2		0.3	2	1	2	2	1	2	1
2	48	1	1	1	2	0	2	2	2	2	2		9	2	0	0	2	4	4	1	2016-04-30 15:00	2	2	2	2	3	0	2	1	1	2		9.5	2	2	1		0	1	0
1	69	1	1	1	1	0	2	2	2	2	2		30	1	0	0	1	1	1	1	2016-04-29 03:35	2	2	2	2	3	1	1	1	2	1		19.6	1	2	2	3	1	2	1
1	71	1	1	1	2	0	2	2	2	2	2		36	1	0	0	1	2	2	1	2016-05-08 21:40	2	2	2	2	3	0	2	1	2	1		7.9	2	2	2	3	1	2	1
1	49	2	1	1	1	0	2	2	2	2	2		26	1	0	0	2	4	4	1	2016-05-07 15:15	2	1	2	1	2	1	2	2	2	2		3.6	2	2	1		0	1	0
1	54	2	1	1	2	0	2	2	2	2	2		75	1	0	0	2	4	4	1	2016-05-26 17:56	2	1	2	1	2		2	1	2	1		23.7	1	1	2	1	1	2	1
2	41	2	2	1	2	0	2	1	2	2	2		32	2	0	0	2	4	4	1	2016-08-04 22:30	2	2	2	2	3	0	1	1	1	2		0.3	2	2	2	2	1	2	1
1	55	2	1	2	2	0	2	2	2	2	2		40	2	0	0	1	1	1	1	2016-08-15 22:30	2	2	2	2	3	0	1	2	2	2			2	1	2	4	1	2	1
2	80	1	1	1	2	0	2	1	1	2	2		17	1	0	0	2	4	4	1	2016-07-23 22:45	2	2	2	2	3	1	2	1	1	2		11.2	2	1	2	3	1	2	1
1	78	2	1	1	2		2	1	2	2	2		31	2	0	0	1	2	2	1	2016-05-01 16:05	2	2	2	2	3		2	1	2	1		7.7	2	2	1		1		
2	44	1	1	1	2	0	2	2	2	2	2		22	2	0	0	2	4	4	1	2016-09-01 17:59	2	2	2	2	3	0	2	1	1	2		17.1	2	2	2	2	1	2	1
2	18	1	1	1	1	0	2	2	2	2	2		24	1	0	0	2	4	4	1	2016-09-03 20:25	2	2	2	2	3	0	2	1	1	2		14.7	2	2	1		0	1	0
1	53	1	1	1	2	0	2	2	2	2	2		30	2	0	0	2	4	4	1	2016-10-14 03:40	2	2	2	2	3	0	2	1	1	2		26.9	2	2	2	2	1	2	1
1	46	1	1	1	2	0	2	2	2	2	2		30	2	0	0	1	2	2	1	2016-10-12 17:50	2	2	2	2	3	1	2	1	2	1		31.9	2	2	2	4	1	2	1
1	29	1	1	1	2	0	2	2	2	2	2		44	2	0	0	2	4	4	1	2016-10-26 02:40	2	2	2	2	3		2	1	1	2		3.9	99	2	1		1	1	1
2	42	2	1	2	2		2	2	2	2	2		42	1	0	0	2	4	4	1	2016-10-19 09:15	2	2	2	2	3	1	2	1	1	2		15.4	2	2	1		1		
2	30	2	1	1	2	0	2	2	2	2	2		40	2	0	0	1	2,9	2,9	1	2016-11-07 05:45	1	2	2	2	3	1	2	2	2	2		0.3	1	1	1		1	1	1
1	55	1	1	1	2	0	2	1	2	2	2		41	1	0	0	2	4	4	1	2016-12-19 06:55	2	2	2	2	3		2	1	1	1		13.9	2	2	2	2	1	2	1
1	79	1	1	2	2	0	1	2	2	1	1		36	1	0	0	1	2	2	1	2016-10-31 10:57	2	2	2	2	3	0	2	1	1	2		17.4	2	2	2	1	1	2	1
1	62	2	1	1	2	0	2	2	2	2	2		14	2	0	0	1	2	2	1	2017-01-31 21:43	2	2	2	2	3	0	2	1	2	1		3.8	1	2	2	3	1	2	1
2	78	2	1	1	2	1	2	1	2	2	2		56	2	0	0	2	4	4	1	2016-09-10 16:14	2	2	2	2	3	0	2	2	2	2		21.5	2	2	1		1	1	1
2	34	1	1	1	2	0	2	2	2	2	2		20	2	1	0	2	4	4	1	2016-07-10 08:03	2	2	2	2	3	1	1	2	2	2		4.8	2	2	1		1	1	1
1	81	2	1	1	2	0	2	1	1	2	2		30	1	0	0	2	4	4	1	2016-04-28 22:40	2	2	2	1	2	0	2	1	2	1		30	2	2	2	3	1	2	1
1	52	2	1	1	1	0	2	2	2	2	2		27	1	0	0	2	4	4	1	2017-01-26 01:00	2	2	1	2	1	0	2	1	1	2		20	2	2	2	3	1	2	1
1	28	2	1	2	2	0	2	2	2	2	2		19	2	1	0	2	4	4	1	2016-12-28 16:39	2	2	2	2	3	0	2	1	1	2		14.3	2	2	1		1	2	1
2	47	1	2	2	2	0	2	2	2	2	2		34	2	0	0	2	4	4	1	2017-01-05 19:40	2	2	2	2	3	0	2	2	2	2		15.4	2	2	1		1	2	1
1	69	2	1	2	2	0	2	1	2	2	2		35	1	1	0	2	4	4	1	2017-02-25 17:10	2	2	2	2	3	0	2	2	2	2		20.2	2	2	1		1	1	1
1	39	2	1	2	1	0	2	1	2	2	2		41	1	0	0	2	4	4	1	2017-03-09 23:12	2	2	2	1	2	1	2	1	1	2		7.1	2	2	1		0	1	0
1	36	1	1	2	2	0	2	2	1	2	2		34	2	1	0	1	2	2	1	2017-03-08 18:30	2	2	2	2	3	0	2	1	1	2		8.6	2	2	1		0	1	0
1	20	2	1	1	1	0	2	2	2	2	2		41	1	0	0	1	1	1	1	2017-03-15 18:45	1	2	2	2	3	1	2	1	2	1		32.9	1	2	2	2	1	2	1
1	51	1	1	1	1	0	2	2	1	2	2		43	1	0	0	2	4	4	1	2017-06-05 16:34	2	1	1	2	1	1	2	1	1	1		32.6	2	2	1	1	0	1	0
1	58	2	1	1	2	0	2	2	2	2	2		19	2	0	0	2	4	4	1	2017-02-08 03:50	2	2	2	2	3	0	2	1	1	2		13.6	2	2	1		0	1	0
1	76	2	1	1	2	0	1	1	2	2	2		20	2	0	0	1	2	2	1	2017-03-22 02:30	2	2	2	2	3	1	2	2	2	2		7.2	1	1	2	3	1	2	1
2	56	1	1	2	2	0	2	1	2	1	2		27	2	1	0	2	4	4	1	2017-03-15 19:50	2	2	2	2	3	1	2	1	2	1		31.7	2	2	2	2	1	2	1
1	63	2	1	1	1	0	1	1	1	2	2		26	1	1	0	2	4	4	1	2017-07-14 16:37	2	2	1	2	1	1	2	1	1	1		29.9	2	2	2	1	1	2	1
2	50	1	1	1	2	0	2	2	2	2	2		33	2	0	0	1	1	1	1	2017-04-15 06:00	2	2	2	2	3	0	1	1	1	2		20.8	2	2	2	2	1	2	1
2	47	2	1	1	2	0	2	1	1	2	1		21	1	0	0	2	4	4	1	2017-09-25 12:55	2	2	2	2	3	1	1	1	1	1		14.5	1	2	2	4	1	2	1
2	25	1	2	1	2	0	2	2	2	2	2		10	2	0	0	2	4	4	1	2017-10-12 10:10	2	2	2	2	3		2	1	1	1		15.1	99	2	1		0	1	0
1	70	1	1	1	2	0	2	2	2	2	2		30	2	0	0	2	4	4	1	2017-11-29 11:30	2	2	2	2	3		2	1	1	2		10	2	2	2	3	1	2	1
1	49	2	1	1	2		2	2	2	2	2		67	1	1	0	1	1	1	1	2017-12-17 15:15	2	2	1	2	1	0	2	1	1	1		40.9	2	2	1		1		
1	75	2	1	1	2	0	2	1	2	2	2		25	2	0	0	2	4	4	1	2017-10-11 18:00	2	2	2	2	3	1	2	1	1	2		2.3	2	2	1		1	1	1
1	39	1	1	2	1	0	2	2	2	2	2		15	1	0	0	1	1	1	1	2017-12-24 21:50	2	2	2	1	2	0	2	1	1	1		28.7	2	1	1		0	1	0
1	28	1	2	2	2	1	2	2	2	2	2		28	2	0	0	2	4	4	1	2017-12-26 19:09	2	2	2	2	3	0	2	2	2	2		7	2	2	2	2	1	2	1
1	57	1	2	2	1	0	2	2	2	2	2		16	2	0	0	2	4	4	1	2017-12-29 00:40	2	2	2	2	3	0	2	1	1	2		7.6	2	2	1		0	1	0
1	62	2	1	1	1	0	2	1	2	2	2		13	1	0	0	2	4	4	1	2018-03-03 02:25	2	2	2	1	2	1	2	2	2	2		0.2	2	2	1		0	1	0
1	28	2	1	2	2	0	2	2	2	2	2		29	2	0	0	2	4	4	1	2018-03-26 23:40	2	2	2	2	3	0	2	1	1	2		10.3	2	2	1		1	1	1
1	50	2	1	2	1	0	2	2	2	2	2		49	1	0	0	2	4	4	1	2018-03-15 18:25	2	2	1	2	1	0	2	2	2	2		7.5	2	2	1		0	1	0
1	75	2	1	1	1	0	2	2	2	2	2		15	1	0	0	2	4	4	1	2018-03-14 16:28	2	1	1	2	1	0	2	2	2	2		6.8	2	2	1		0	1	0
1	66	2	1	1	2	0	2	1	1	2	1		20	1	0	0	2	4	4	1	2018-07-26 00:30	2	2	2	2	3	1	2	1	1	2		22.5	2	1	2	3	1	1	1
2	22	1	1	1	2	0	2	2	2	2	2		5	2	0	0	1	1	1	1	2018-06-01 17:30	2	2	2	2	3	0	2	1	1	2		1	2	2	1		0	1	0
1	46	2	1	1	2	0	2	2	2	2	2		17	1	1	1	1	1	1	1	2018-11-02 22:32	1	2	2	1	2	0	2	1	1	2		12.6	2	2	1		0	1	0
2	44	2	1	1	1	1	2	2	2	2	2		40	1	0	0	1	1	1	1	2018-09-19 17:50	2	2	2	1	2	0	2	1	1	2		8.9	2	1	1		0	1	0
1	64	2	1	1	1	0	2	2	2	2	2		43	1	1	1	3	9	9	1	2017-12-13 23:10	1	2	2	1	2	1	2	1	1	1		1.2	1	2	2	4	1	2	1

1	71	1	1	2	2	0	2	2	2	2	2		51	1	1	0	1	1	1	1	2018-11-09 13:10	2	2	2	2	3	0	2	1	1	1		14.3	2	1	2	3	1	2	1
1	51	1	1	1	2	0	2	2	2	2	2		31	1	1	1	1	1	1	1	2018-09-29 00:05	2	2	2	2	3	0	2	2	2	2			2	1	2	3	1	2	1
1	50	2	1	1	2	0	2	2	2	2	2		43	2	0	0	1	1	1	1	2018-12-15 12:45	2	2	2	2	3	0	2	1	2	1		4.3	2	2	2	4	1	2	1
1	65	1	1	1	1	0	2	2	2	2	2		46	1	0	0	1	1	1	1	2017-11-14 17:45	2	2	2	1	2	0	2	1	1	1		5	2	2	1		0	1	0
1	83	2	2	1	2		2	1	2	2	2		29	1	0	0	1	1	1	1	2018-04-08 22:53	2	2	2	2	3		2	1	1	1		17	2	2	1		1		
1	53	1	1	1	1	0	2	2	2	2	2		45	1	0	0	1	1	1	1	2018-09-24 13:45	2	2	2	2	3		2	1	1	2		9.2	2	2	1		0	1	0
1	41	1	2	1	1	0	2	2	2	2	2		27	1	0	0	1	1	1	1	2018-07-16 05:45	2	2	2	1	2	0	2	1	1	2		5.9	2	1	1		0	1	0
2	56	2	1	2	1	0	2	2	2	2	2		26	1	0	0	2	4	4	1	2018-03-30 20:33	2	2	1	2	1	0	2	2	2	2		4.8	2	2	1		0	1	0
2	49	2	2	2	2	0	2	2	2	2	2		30	2	0	1	2	4	5	1	2018-09-02 00:20	1	2	2	2	3		2	1	2	1		26.3	2	2	1		0	1	0
1	73	2	1	1	1	0	2	2	2	2	2		26	1	0	0	1	1	1	1	2018-08-19 13:45	2	2	2	1	2	1	2	1	1	2		22.6	2	2	1		0	1	0
2	39	2	1	2	1		2	2	1	2	2		25	1	0	1	2	4,9	4,9	1	2017-10-06 17:59	1	2	2	2	3		1	1	1	1		12.2	2	2	1		1		
1	49	2	1	1	1	0	2	1	2	2	2		26	1	0	0	2	4	4	1	2017-12-18 20:45	2	2	2	1	2	0	2	1	1	1		4.6	2	2	1		0	1	0
1	69	1	1	1	2	0	2	2	2	2	2		30	1	0	0	2	4	4	1	2018-06-23 15:00	2	2	2	2	3		2	1	1	2		0.3	2	1	2	1	1	2	1
2	27	1	2	1	2		2	2	2	2	2		24	2	0	0	1	1	1	1	2018-12-24 20:10	2	2	2	2	3		2	1	2	1		29.6	99	2	1		1		
1	46	1	1	2	1	0	2	2	2	2	2		21	1	0	0	2	4	4	1	2018-12-18 17:52	2	2	2	1	2	0	2	1	1	2		9	2	2	1		0	1	0
