# Supplementary material for: The relationship between body mass index and neurologic outcomes in survivors of out-of-hospital cardiac arrest treated with targeted temperature management
Source: PLoS One. 2022 Mar 29;17(3):e0265656. doi: 10.1371/journal.pone.0265656 (PMC8963585; doi:10.1371/journal.pone.0265656)
Supplement: Attachment — Submitted filename: Response to reviewers.docx [file pone.0265656.s002.docx]

Dear Dr. Kim,

Thank you for submitting your manuscript to PLOS ONE. After careful consideration, we feel that it has merit but does not fully meet PLOS ONE’s publication criteria as it currently stands. Therefore, we invite you to submit a revised version of the manuscript that addresses the points raised during the review process.

==============================

The authors concluded that BMI is not associated with neurologic outcomes in out of hospital cardiac arrest treated with TTM.

Can the authors report what were the variables used in multivariables analysis?
==============================

Please submit your revised manuscript by Mar 05 2022 11:59PM. If you will need more time than this to complete your revisions, please reply to this message or contact the journal office at [plosone@plos.org](mailto:plosone@plos.org). When you're ready to submit your revision, log on to <https://www.editorialmanager.com/pone/> and select the 'Submissions Needing Revision' folder to locate your manuscript file.

Please include the following items when submitting your revised manuscript:

- A rebuttal letter that responds to each point raised by the academic editor and reviewer(s). You should upload this letter as a separate file labeled 'Response to Reviewers'.
- A marked-up copy of your manuscript that highlights changes made to the original version. You should upload this as a separate file labeled 'Revised Manuscript with Track Changes'.
- An unmarked version of your revised paper without tracked changes. You should upload this as a separate file labeled 'Manuscript'.

If you would like to make changes to your financial disclosure, please include your updated statement in your cover letter. Guidelines for resubmitting your figure files are available below the reviewer comments at the end of this letter.

If applicable, we recommend that you deposit your laboratory protocols in [protocols.io](http://protocols.io/) to enhance the reproducibility of your results. Protocols.io assigns your protocol its own identifier (DOI) so that it can be cited independently in the future. For instructions see: <https://journals.plos.org/plosone/s/submission-guidelines#loc-laboratory-protocols>. Additionally, PLOS ONE offers an option for publishing peer-reviewed Lab Protocol articles, which describe protocols hosted on [protocols.io](http://protocols.io/). Read more information on sharing protocols at <https://plos.org/protocols?utm_medium=editorial-email&utm_source=authorletters&utm_campaign=protocols>.

We look forward to receiving your revised manuscript.

Kind regards,

Jignesh K. Patel
Academic Editor
PLOS ONE

Comments from the editors and reviewers:

Editors’ comments:

The authors concluded that BMI is not associated with neurologic outcomes in out of hospital cardiac arrest treated with TTM.

1) Can the authors report what were the variables used in multivariables analysis?

- Thank you for your comments. I agree with your comment and I added variables in mnauscripts.

However, in the multivariate logistic regression analysis, we found that BMI was not associated with poor neurologic outcome after adjusting for sex, age, history of hypertension, diabetes mellitus, lung disease, renal disease, arrest location, initial arrest rhythm, witnessed collapse, bystander CPR, arrest etiology, anoxic time, shock after ROSC.

2) Please include the full name of the IRB or ethics committee who approved or waived your study.

🡪 Thank you for your comments. I added IRB in Manuscripts.

The study included an informed consent form approved by all participating hospitals, including the institutional review board (IRB) of Seoul St. Mary’s Hospital (XC150IMI0081K)

Reviewer #1: A very interesting paper that helps argue against the thinking that higher BMI is always correlated with worse outcomes. I appreciated the discussion section that tried to provide reasons behind the observed findings in the study.

1) There seems to be a formatting issue with the manuscript and I was unable to view the "Obese" column for Table 1 and multivariate analysis in Table 2.

- Thank you for your comments. I changed Tables 1 and Table 2 that can view “Obeses” columns.

**Table 1. Baseline Characteristics of Patients Stratified According to Body Mass Index Classification**

|  | **Total** | **Underweight** | | | **Normal** | | **Overweight** | | **Obese** | | **p-value** |
| --- | --- | --- | --- | --- | --- | --- | --- | --- | --- | --- | --- |
|  | **(n=1315)** | **(n=112)** | | | **(n=798)** | | **(n=332)** | | **(n=73)** | |  |
| Male sex | 933 (71.0%) | 65 (58.0%) | | | 579 (72.6%) | | 249 (75.0%) | | 40 (54.8%) | | < 0.001 |
| Age, years,  median (IQR) | 53.0  (48.0-70.0) | 60.0  (47.5-72.0) | | | 59.0  (48.0-70.0) | | 57.0  (47.0-69.0) | | 54.0  (44.0-63.0) | | 0.042 |
| Comorbidities |  |  | | |  | |  | |  | |  |
| CAD | 194 (14.8%) | 7 (6.3%) | | | 120 (15.0%) | | 56 (16.9%) | | 11 (15.1%) | | 0.053 |
| Hypertension | 473 (36.0%) | 19 (17.0%) | | | 281 (35.2%) | | 134 (40.4%) | | 39 (53.4%) | | < 0.001 |
| Diabetes mellitus | 318 (24.2%) | 15 (13.4%) | | | 192 (24.1%) | | 84 (25.3%) | | 27 (37.0%) | | 0.003 |
| CVA | 73 (5.6%) | 8 (7.1%) | | | 47 (5.9%) | | 15 (4.5%) | | 3 (4.1%) | | 0.641 |
| Lung disease | 84 (6.4%) | 22 (19.6%) | | | 41 (5.1%) | | 17 (5.1%) | | 4 (5.5%) | | < 0.001 |
| Renal disease | 100 (7.6%) | 6 (5.4%) | | | 57 (7.1%) | | 28 (8.4%) | | 9 (12.3%) | | 0.297 |
| BMI, kg/m²,  median (IQR) | 23.36  (20.96-25.71) | 17.30  (16.26-17.96) | | | 22.48  (20.76-23.76) | | 26.49  (25.71-27.72) | | 32.67  (31.06-35.23) | | < 0.001 |
| Arrest location |  |  | | |  | |  | |  | | 0.366 |
| Home/residence | 674 (51.3%) | 64 (57.1%) | | | 410 (51.4%) | | 160 (48.2%) | | 40 (54.8%) | |  |
| Other | 641 (48.7%) | 48 (42.9%) | | | 388 (48.6%) | | 172 (51.8%) | | 33 (45.2%) | |  |
| Initial arrest rhythm |  |  | | |  | |  | |  | | 0.012 |
| Shockable | 443 (33.7%) | 26 (23.2%) | | | 270 (33.8%) | | 128 (38.6%) | | 19 (26.0%) | |  |
| Non-shockable | 872 (66.3%) | 86 (76.8%) | | | 528 (66.2%) | | 204 (61.4%) | | 54 (74.0%) | |  |
| Witnessed collapse | 910 (69.2%) | 74 (66.1%) | | | 534 (66.9%) | | 247 (74.4%) | | 55 (75.3%) | | 0.047 |
| Bystander CPR | 810 (61.6%) | 66 (58.9%) | | | 486 (60.9%) | | 210 (63.3%) | | 48 (65.8%) | | 0.701 |
| Arrest etiology |  |  | | |  | |  | |  | | 0.001 |
| Cardiac etiology | 812 (61.7%) | 52 (46.4%) | | | 492 (61.7%) | | 226 (68.1%) | | 42 (57.5%) | |  |
| Other etiology | 503 (38.3%) | 60 (53.6%) | | | 308 (38.3%) | | 106 (31.9%) | | 31 (42.5%) | |  |
| Anoxic time, minutes, median (IQR) | 3.0  (17.0-43.0) | 24.0  (13.0-38.0) | | | 28.0  (17.0-40.0) | | 28.0  (17.0-43.0) | | 32.0  (15.0-45.5) | | 0.047 |
| TTM methods |  |  | | |  | |  | |  | | 0.756 |
| Surface cooling | 1178 (89.6%) | 99 (88.4%) | | | 717 (89.8%) | | 296 (89.2%) | | 66 (90.4%) | |  |
| Intravascular cooling | 122 (9.3%) | 13 (11.6%) | | | 71 (8.9%) | | 31 (9.3%) | | 7 (9.6%) | |  |
| Other | 15 (1.1%) | 0 (0.0%) | | | 10 (1.3%) | | 5 (1.5%) | | 0 (0.0%) | |  |
| TTM target temperature |  |  | | |  | |  | |  | | **0.275** |
| 33℃ | 1035 (78.7%) | 85 (75.9%) | | | 618 (77.4%) | | 272 (81.9%) | | 60 (82.2%) | |  |
| 36℃ | 280 (21.3%) | 27 (24.1%) | | | 180 (22.6%) | | 60 (18.1%) | | 13 (17.8%) | |  |
| Induction time, hours, median (IQR) | 2.33 (1.08-4.25) | 1.42  (1.00-3.50) | | | 2.25  (1.07-4.19) | | 3.00  (1.83-4.92) | | 3.75  (1.58-5.88) | | < 0.001 |
| Rewarming time, hours, median (IQR) | 13.67  (9.27-16.00) | 13.00  (8.63-17.80) | | | 13.5  (9.00-16.00) | | 14.00  (10.00-16.00) | | 14.00  (8.50-16.00) | | 0.226 |
| ROSC to TTM, hours, median (IQR) | 3.42  (2.17-4.87) | 3.62  (2.18-7.21) | | | 3.45  (2.17-5.06) | | 3.33  (2.20-4.73) | | 4.00  (2.83-5.13) | | 0.466 |
| ROSC to target, hours, median (IQR) | 6.20  (4.28-9.00) | 5.42  (3.89-7.38) | | | 6.22  (4.26-9.00) | | 6.83  (4.88-9.72) | | 7.13  (4.93-10.10) | | < 0.001 |
| Shock after ROSC | 638 (48.5%) | 41 (36.6%) | | | 396 (49.8%) | | 164 (49.5%) | | 37 (50.7%) | | 0.067 |
| Reperfusion |  |  | | |  | |  | |  | | < 0.001 |
| None | 831 (63.2%) | 91 (81.3%) | | | 495 (62.0%) | | 204 (61.4%) | | 41 (56.2%) | |  |
| CAG only | 285 (21.7%) | 13 (11.6%) | | | 191 (23.9%) | | 62 (18.7%) | | 19 (26.0%) | |  |
| CAG with PCI | 199 (15.1%) | 8 (7.1%) | | | 112 (14.0%) | | 66 (19.9%) | | 13 (17.8%) | |  |
| tPA | 40 (3.0%) | 3 (2.7%) | | | 24 (3.0%) | | 12 (3.6%) | | 1 (1.4%) | | 0.774 |
| ECMO | 59 (4.5%) | 2 (1.8%) | | | 35 (4.4%) | | 18 (5.4%) | | 4 (5.5%) | | 0.428 |
| Adverse events |  |  | | |  | |  | |  | |  |
| Seizure | 316 (24.0%) | 39 (34.8%) | | | 194 (24.3%) | | 66 (19.9%) | | 17 (23.3%) | | 0.016 |
| Bleeding | 64 (4.9%) | 5 (4.5%) | | | 40 (5.0%) | | 17 (5.1%) | | 2 (2.7%) | | 0.96 |
| Pneumonia | 512 (38.9%) | 55 (49.1%) | | | 304 (38.1%) | | 127 (38.3%) | | 26 (35.6%) | | 0.138 |
| Sepsis | 153 (11.6%) | 15 (13.4%) | | | 89 (11.2%) | | 39 (11.7%) | | 10 (13.7%) | | 0.844 |
| Rearrest | 248 (18.9%) | 15 (13.4%) | | | 140 (17.5%) | | 74 (22.3%) | | 19 (26.0%) | | 0.044 |
| Six-month neurologic outcome |  |  | | |  | |  | |  | | 0.111 |
| Good outcome | 410 (31.2%) | 25 (22.3%) | | | 262 (32.8%) | | 104 (31.3%) | | 19 (26.0%) | |  |
| Poor outcome | 905 (68.8%) | 87 (77.7%) | | | 536 (67.2%) | | 228 (68.7%) | | 54 (74.0%) | |  |
| Six-month mortality | 541 (41.1%) | 39 (34.8%) | | | 342 (42.9%) | | 135 (40.8%) | | 25 (34.2%) | | 0.234 |
|  | | |  |  | |  | |  | |  |  |

**Table 2. Univariate and Multivariate Logistic Regression Analysis for Poor Neurologic Outcome at 6 Months**

| **Variables** | **Univariate analysis** | | | **Multivariate analysis** | | |
| --- | --- | --- | --- | --- | --- | --- |
|  | Unadjusted OR | 95% CI for the OR | p-value | Adjusted OR | 95% CI for the OR | p-value |
| Sex (Male/Female) | 0.602 | 0.459-0.790 | < 0.001 | 0.778 | 0.535-1.131 | 0.189 |
| Age (per years) | 1.032 | 1.024-1.039 | < 0.001 | 1.044 | 1.031-1.057 | < 0.001 |
| Comorbidities |  |  |  |  |  |  |
| Coronary heart disease | 1.263 | 0.917-1.739 | 0.154 |  |  |  |
| Hypertension | 1.545 | 1.202-1.987 | 0.001 | 1.037 | 0.705-1.524 | 0.853 |
| Diabetes mellitus | 2.109 | 1.557-2.857 | < 0.001 | 1.299 | 0.838-2.013 | 0.241 |
| CVA | 1.409 | 0.817-2.431 | 0.218 |  |  |  |
| Lung disease | 4.026 | 1.996-8.121 | < 0.001 | 0.971 | 0.425-2.218 | 0.945 |
| Renal disease | 2.334 | 1.367-3.987 | 0.002 | 1.242 | 0.625-2.466 | 0.536 |
| Arrest location (Home/Others) | 1.674 | 1.323-2.119 | < 0.001 | 1.092 | 0.784-1.520 | 0.603 |
| Initial arrest rhythm (Shockable/Non-shockable) | 0.089 | 0.068-0.117 | < 0.001 | 0.183 | 0.126-0.264 | < 0.001 |
| Witnessed collapse | 0.32 | 0.238-0.431 | < 0.001 | 0.687 | 0.460-1.026 | 0.066 |
| Bystander CPR | 0.677 | 0.530-0.866 | 0.002 | 1.099 | 0.780-1.550 | 0.589 |
| Cardiac arrest etiology | 0.135 | 0.097-0.187 | < 0.001 | 0.232 | 0.148-0.364 | < 0.001 |
| Time fromaAnoxic time (per minutes) | 1.061 | 1.051-1.071 | < 0.001 | 1.064 | 1.052-1.076 | < 0.001 |
| TTM target temperature (33℃/36℃) | 0.965 | 0.726-1.282 | 0.805 |  |  |  |
| ROSC to TTM start time (per hours) | 0.98 | 0.940-1.021 | 0.332 |  |  |  |
| ROSC to target temperature time (per hours) | 1 | 0.999-1.001 | 0.513 |  |  |  |
| Shock after ROSC (Yes/No) | 2.819 | 2.209-3.597 | < 0.001 | 1.888 | 1.362-2.618 | < 0.001 |
| BMI, kg/m² |  |  |  |  |  |  |
| Normal (15.5-24.9) | Reference | - | - |  |  |  |
| Underweight (< 18.5) | 1.701 | 1.065-2.718 | 0.026 | 1.668 | 0.903-3.080 | 0.102 |
| Overweight (25.0-29.9) | 1.072 | 0.814-1.411 | 0.622 | 1.444 | 0.981-2.125 | 0.062 |
| Obese (> 30.0) | 1.389 | 0.807-2.392 | 0.236 | 1.421 | 0.684-2.951 | 0.347 |

*Abbreviations: OR* odds ratio, *CI* confidence interval, *CVA* Cerebrovascular accident, *CPR* cardiopulmonary resuscitation, *TTM* targeted temperature management, ROSC return of spontaneous circulation, *BMI* body mass index
